# Geographic Distribution and Genetic Diversity of Rice Stripe Mosaic Virus in Southern China

**DOI:** 10.3389/fmicb.2018.03068

**Published:** 2018-12-10

**Authors:** Xin Yang, Biao Chen, Tong Zhang, Zhanbiao Li, Chenhui Xu, Guohui Zhou

**Affiliations:** Guangdong Province Key Laboratory of Microbial Signals and Disease Control, College of Agriculture, South China Agricultural University, Guangzhou, China

**Keywords:** rice stripe mosaic virus, *Cytorhabdovirus*, virus genetic diversity, rice viral disease, leafhopper-transmitted virus

## Abstract

Rice stripe mosaic virus (RSMV) transmitted by the leafhopper *Recilia dorsalis* is a tentative new species in the genus *Cytorhabdovirus* identified recently in South China. To explore its geographic distribution and genetic diversity, field investigation and viral whole-genome sequencing were conducted in this study. The results indicated that RSMV was present in the rice samples collected across southern China. Twelve representative samples from different geographical regions were selected for viral whole-genome sequencing and the viral genome variation was analyzed in combination with a previously reported RSMV isolate. Identity analysis showed that the genome sequences of 13 RSMV isolates were highly conserved with nucleotide identities over 99.4%. There was a strong negative selection pressure during the evolution of RSMV with more transitions (72.08%) than transversions (27.92%) found between the RSMV isolates. Among the seven genes encoded by RSMV, the P gene was the most variable, followed by N, M, L, and G; the P3 and P6 amino acid sequences were not found to be mutated and no mutations were found in the non-coding region. A phylogenetic tree based on the RSMV whole-genome nucleotide sequences revealed that all RSMV isolates clustered in two groups based on geographical origin. Notably, the L proteins of the Guangxi and Hainan isolates had five and one specific amino acid sites, respectively, suggesting that the L gene has undergone environmental adaptive variation during the dispersal of RSMV.

## Introduction

Rice (*Oryza sativa*) is a major staple crop worldwide with more than 90% of rice production coming from Asia (Bheemanahalli et al., 2016). Several viruses has been reported infecting rice which result in yield losses (Uehara-Ichiki et al., 2013) with rice stripe mosaic virus (RSMV) representing an emergent pathogen since it was first detected in southern China in 2015 (Yang et al., 2017a). Infected rice plants show slight dwarfing, yellow stripes, mosaic and twisted tips on leaves, increased tillering, unfilled grains, and yield losses (Yang et al., 2017a). RSMV is a tentative new species of the genus *Cytorhabdovirus* of the family *Rhabdoviridae* (Yang et al., 2017a) which is transmitted by the leafhopper *Recilia dorsalis* (Yang et al., 2017b). It replicates in the cytoplasm of infected cells and has a negative-sense single-strand RNA genome of about 12.7 kb which encodes seven proteins: N, P, P3, M, G, P6, and L (Yang et al., 2017a). Although its host range, genomic information, vector, and transmission characteristics have been studied, the geographic distribution and genetic diversity of RSMV remain unclear.

In this study, the distribution of RSMV in southern China was determined analyzing 459 rice samples collected from southern China during 2017 and 2018. The genetic diversity of 12 RSMV was explored by determining the whole-genome sequence of RSMV isolates from different geographical areas. These results provided basic information for further analyses of the genetic variability of RSMV which will be useful for developing strategies towards the management of this virus.

## Materials and Methods

### Disease Investigation and RSMV Detection

Between May 2017 and May 2018, field surveys were performed in three provinces (Guangdong, Guangxi, and Hainan) of southern China. Rice samples showing leaf mosaic or stripe symptoms were sampled (Table 1). RT-PCR detection of RSMV was carried out as previously described (Yang et al., 2017a) and some positive PCR products were verified by directly sequencing.

**Table 1:** Rice sample collection and RSMV detection in southern China during 2017 and 2018.

Location		Sampling time	No. of samples tested	No. of RSMV positive samples
Provinces	Countries			
Guangdong (GD)	Taiping (TP)	2017/05	37	37
	Luoping		10	10
	Songgui (SG)		22	18
	Taiping	2017/06	24	24
	Luoping		24	24
	Xingning	2017/07	10	0
	Meizhou		10	0
	Taiping	2017/09	19	13
	Luoping		63	59
	Luojing (LJ)		7	7
	Songgui		26	15
	Zhaoqing		7	0
	Boluo	2017/10	3	0
	Leizhou	2018/05	10	0
	Zhanjiang		12	0
	Maomin		11	0
	Shaoguang		15	0
Guangxi (GX)	Wuzhou (WZ)	2017/06	13	8
	Hezhou (HZ)		25	16
	Laibin		6	1
	Nanning		8	0
	Shanglin		10	0
	Guiling		10	0
	Yuling		11	9
	Wuzhou	2018/05	14	13
	Beiliu		4	4
	Qinzhou		8	4
Hainan (HN)	Lingshui (LS)	2017/05	8	1
	Timeng (TM)		6	1
	Dingan		5	0
	Tunchang		4	0
	Danzhou		7	0
	Sanya		5	0
	Haikou		5	0

### RSMV Genomic Sequencing

Ten pairs of specific primers (Table 2) were designed according to the reported RSMV Guangdong isolate (GenBank Accession No. KX525586.2) using the PrimerSelect program in DNASTAR 7.1 (Lasergene, United States) so as to amplify the complete genome sequence that including the 3′-terminal and 5′-terminal sequences. Total RNAs from RSMV-infected samples were used as a template and a one-step RNA PCR kit (TaKaRa, Dalian, China) was used according to manufacturer’s instructions. The RT-PCR program was 50°C for 30 min, 94°C for 2 min; 35 cycles of 94°C for 30 s, 55°C for 30 s, and 72°C for 1 min; and 72°C for 10 min. The PCR products were analyzed by electrophoresis in 1.2% agarose gel (stained with 5 μl/100 ml GoldView), purified using the AxyPrePTM DNA gel Extraction Kit (AxyGEN), and sequenced directly in both directions with three replicates (Shanghai Biotech, Shanghai, China). Sequence assembly and analysis was performed using the SeqMan program in DNASTAR 7.1 (Lasergene), and the whole genome sequence of each isolate was obtained (Supplementary Table S7) and submitted to the Genbank. The accession numbers of each sequenced RSMV isolate are listed in Table 3.

**Table 2:** Primer sequences used for RSMV genome amplification.

Name	Sequence (5′-3′)	Reference position
Start-F	AAGGAAGTTGCGTTGCGAAC	1–20
N-R	TTAAGCCTTGGTCTGGAAGATG	1544–1565
N-F1	TACGGATAATACTGGCAGAAGC	1435–1456
P-R1	AGGCACAAGATCACAGACGAT	2854–2874
P-F1	TCAGTTACTACCTGTGTGGCA	2801–2821
G-R	TCTCAGTCATCACCTTGCTAC	4073–4093
M-F1	AACTTCAGTGTCCAGCCTAC	4001–4020
G-R1	GTCGTGCTCCTTAGACCTCTT	3456–3476
G-F1	TACACCATCTCCAAGCCTCA	5303–5322
L-R1	ATCCAGCCTAGTGATTTCATCC	6417–6438
L-F1	ACCTTGATGACGGTGGTCTAT	6282–6302
L-R	TCCGTCTTTCATAGCCTTCAG	7658–7678
L-FA	TGGTTGGAATTGAGACGTAAGG	7598–7619
L-RA	GTTGATTCACTGGCAATTGCG	8977–8997
L-FB	TAGTGACCATACCTCGAAGCT	8871–8891
L-RB	TCTTCCTGATTACGCTCACGA	10464–10484
L-FC	AAGGAGGCACAGTGGGACTT	10406–10425
L-RC	TCTGCACATCAGCTTTGTAGTG	11441–11462
L-FD	AGACAGGAATGTAGTGAGCTG	11359–11379
END-R	AAGGAAGTTGTGTGTTGCGAACA	12752–12774

*The primers were designed on reference genome of RSMV LD isolated (GenBank Acc. No. KX525586.2).*

**Table 3:** Whole-genome nucleotide identities (%) of RSMV isolates from southern China.

Provence origin	RSMV isolates	Access. No.	RSMV isolates												
			LD	TP1	TP2	LJ1	LJ2	SG1	SG2	WZ9	WZ12	HZ5	HZ7	LS	TM
GD	LD	KX525586.2	–	99.6	99.5	99.4	99.5	99.4	99.4	99.4	99.4	99.4	99.7	99.5	99.5
	TP1	MH720464		–	99.7	99.6	99.6	99.6	99.6	99.6	99.5	99.5	99.5	99.6	99.6
	TP2	MH720465			–	99.5	99.6	99.6	99.6	99.5	99.5	99.5	99.5	99.6	99.6
	LJ1	MH720466				–	99.5	99.5	99.5	99.4	99.4	99.4	99.3	99.4	99.4
	LJ2	MH720467					–	99.5	99.5	99.5	99.5	99.5	99.4	99.5	99.5
	SG1	MH720468						–	99.6	99.4	99.4	99.4	99.3	99.4	99.4
	SG2	MH720469							–	99.4	99.4	99.4	99.3	99.4	99.5
GX	WZ9	MH720470								–	100	100	99.7	99.5	99.5
	WZ12	MH720471									–	100	99.7	99.5	99.5
	HZ5	MH720472										–	99.7	99.5	99.5
	HZ7	MH720473											–	99.4	99.4
HN	LS	MH720474												–	99.9
	TM	MH720475													–

### Genomic Sequence Analysis

Multiple sequences analysis was carried out by MAFFT 7.149 software (Katoh and Standley, 2013) using the 12 RSMV isolates sequenced in this study and the previously obtained RSMV (Guangdong Luoding isolate). Molecular diversity among those RSMV isolates was determined using MEGA X (Kumar et al., 2018). Phylogenetic tree was constructed using MEGA X (Kumar et al., 2018) using the Maximum likelihood (ML) method. The genetic distance and base substitution type were calculated by the ML method (Maximum Composite Likelihood model) and ML statistical method with MEGA X, respectively. The extent and distribution of genetic variation among RSMV isolates was estimated by the average number of nucleotide differences per site (π) using DnaSP 5.0 (Librado and Rozas, 2009). Recombination site analysis in RSMV sequences was done using Recombination Detection Program (RDP) 4.97 (Martin et al., 2015). The ratio of non-synonymous and synonymous (d_n_/d_s_) was calculated to estimate the selection pressure using the Datamonkey sever (Weaver et al., 2018) with single likelihood ancestor counting (SLAC), fixed effects likelihood (FEL) and Fast, Unconstrained Bayesian AppRoximation) (FUBAR) methods.

## Results

### RSMV Distribution in Southern China

**FIGURE 1:**
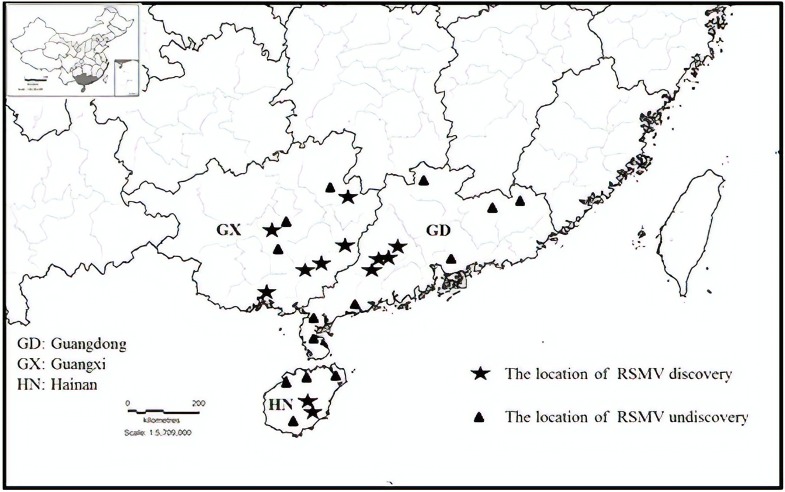
Geographic distribution of rice stripe mosaic virus (RSMV) in southern China during 2017 and 2018.

From May 2017 to May 2018, 459 suspected RSMV-infected rice samples were tested in three provinces (Guangdong, Guangxi, and Hainan) of southern China (Table 1). Results showed that 264 (57.5%) samples were infected with RSMV. Currently, RSMV mainly occurs in southwestern Guangdong, with a disease incidence of typically 5–10% but sometimes greater than 40% in some fields. In certain fields of Wuzhou and Hezhou in Guangxi, which is adjacent to Guangdong, the disease incidence was 1%–5%; RSMV-infected rice samples were also occasionally observed in central parts of Guangxi and Hainan (Figure 1).

### Genomic Sequence Homology of RSMV Isolates

Twelve samples with different geographical origins were sequenced and compared with the sequence of the reference isolate. The 13 sequenced genomes were 12774 bp in length and contained seven ORFs. Nucleotide (nt) sequence identities ranged from 99.4 to 100% among the genome sequences of the 13 isolates (Table 3) indicating high sequence conservation between isolates. Furthermore, the nt identities (99.4–99.7%) among Guangdong isolates were lower than the nt identities (≥ 99.7%) among Guangxi and Hainan isolates. This indicates that the genetic diversity of Guangdong isolates was greater than that of Guangxi and Hainan isolates and suggests that RSMV may have originated in Guangdong. Comparison of the untranslated regions between the genes revealed high variability, with the N-P intergenic region having the highest nucleotide variability (96.1–98.1%) (Supplementary Table S1). Although sequence variability was observed in the untranslated regions between all ORFs, the conserved intergenic region motif (3′-AUUCUUUUUGCUCUGG-5′) was conserved in all isolates tested.

### Nucleotide and Amino Acid Variation Among RSMV Isolates

For each gene, the highest variability was observed in the P gene (nt and amino acid (aa) identities ranged from 98.9 to 100% and 98.9 to 100%, respectively), followed by the N, M, L, and G genes (Supplementary Tables S2–S6). No variability was observed in the P3 and P6 genes (data not shown).

Analysis of the nucleotide variation of the 13 RSMV genome sequences revealed that transitions from A↔G (37.22%) and C↔U (34.86%) occurred more frequently than transversions from A↔U (4.34%), A↔C (9.93%), U↔G (10.29%), and C↔G (3.35%) (Table 4). The above results indicated that the RSMV genome has a mutational bias for A↔G and C↔U transitions.

**Table 4:** Types of nucleotide mutation and base substitution among RSMV isolates.

RSMV isolates	No. of nucleotide mutation		No. of transition		No. of transversion			
	No-synonymous	synonymous	A↔G	C↔U	A↔U	A↔C	U↔G	C↔G
LD	–	–	–	–	–	–	–	–
TP1	7	42	21	19	4	4	7	2
TP2	9	39	18	19	5	6	7	2
SG1	10	58	29	29	3	5	7	3
SG2	8	58	29	28	2	7	6	2
LJ1	7	58	27	25	5	12	5	2
LJ2	5	52	25	24	3	5	6	3
WZ9	7	54	28	23	2	8	8	3
WZ12	7	55	28	24	2	8	8	3
HZ5	7	55	28	24	2	8	8	3
HZ7	7	23	17	26	1	3	4	0
LS	2	26	25	20	3	7	9	2
TM	2	25	25	20	3	7	8	2

**FIGURE 2:**
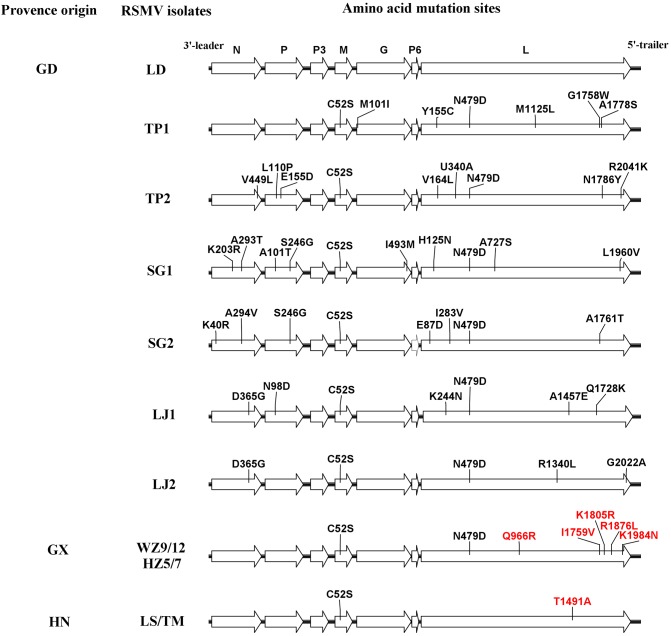
Diagrammatic representation of amino acid mutations among rice stripe mosaic virus (RSMV) isolates. The black and red lines indicate amino acid mutations at random sites among Guangdong RSMV isolates and specific sites among three RSMV isolates with different geographic origins, respectively.

To estimate amino acid mutations (non-synonymous and synonymous) in all RSMV isolates, mutation analyses were performed for each protein encoded by RSMV with the reported isolate used as a reference sequence. The results showed that the majority of mutations were synonymous (Table 4). Furthermore, comparisons of multiple amino acid sequences showed that there were seven amino acid mutations in the N protein that mainly occurred in the Guangdong isolates; in the SG2 isolates, positions 40 and 294 were amino acids R and V, respectively, while in the other isolates they were K and A, respectively; in the SG1 isolate, positions 203 and 293 were R and T, respectively, while in other isolates they were K and A, respectively; in LJ1 and LJ2 isolates there was a G at position 365, while other isolates had a D; and in the TP2 isolate there was an L at position 449, while other isolates had a V. There were six amino acid mutations in the P protein, mostly occurring in the Guangdong isolates; the LJ1 isolate had the amino acid D at position 98, while other isolates had an N; in the SG1 isolate there was a T and G at positions 101 and 246, respectively, while in other isolates they were A and S, respectively; the SG2 isolate had a G at position 246, while other isolates had an S; and the TP2 isolate had a P and D at positions 110 and 155, respectively, while the other isolates had an L and E, respectively. There was one amino acid mutation in the M protein in the LD isolate that had a C at position 52 while the other isolates had an S. There were two amino acid mutations in the G protein, mainly occurring in the Guangdong isolates; the TP1 isolate had an I at position 101, while the other isolates had an M; and the SG1 isolate had an M at position 493, while the other isolates had an I. There were 32 amino acid mutations in the L protein, including six amino acid mutation sites in the Guangxi isolates, namely amino acids D, R, V, R, L, and N at positions 479, 966, 1759, 1805, 1876, and 1984, respectively; the latter five amino acid sites were specific mutation sites that were only found in all Guangxi isolates. There was one specific amino acid mutation site in the Hainan isolate, namely amino acid A at position 1491. The remaining 25 amino acid mutations occurred among Guangdong isolates and no amino acid mutations were found in the P3 and P6 proteins (Figure 2).

### Analysis of Genetic Variation, Recombination and Selection Pressure

**FIGURE 3:**
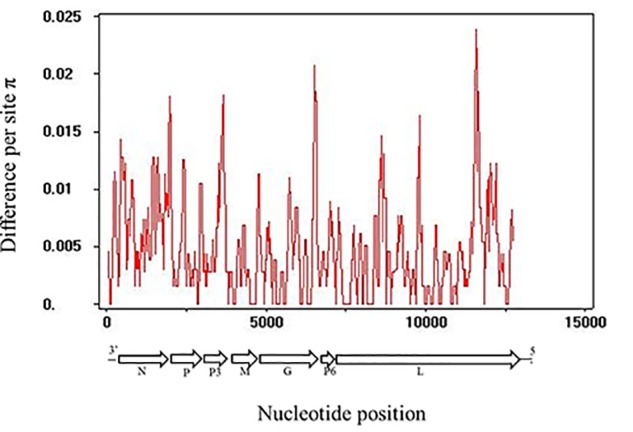
Distribution of genetic variation along RSMV whole genome sequence estimated by nucleotide diversity (π). A 100-nt wide sliding window was used with a 25-nt step size.

The whole genomic RSMV nucleotide diversity (π) was analyzed. The variation rates were mostly below 1.0% with a highest peak at the ending of L gene (Figure 3). There was no evidence of recombination in the seven genes of RSMV (data not shown). The mean values of the d_n_/d_s_ ratios were calculated for the seven genes based on the SLAC method (Table 5). This result showed that the d_n_/d_s_ ratio in all genes was significantly < 1, implying that all RSMV genes are under negative or purifying selection.

**Table 5:** Selection pressure analysis of each gene in RSMV using the SLAC, FEL and FUBAR methods available in the Datamonkey sever.

Gene	SLAC		FEL		FUBAR		d_n_/d_S_
	PS	NS	PS	NS	PS	NS	
N	0	0	0	2	0	27	0.093
P	0	0	0	2	0	22	0.0702
P3	0	0	0	4	0	7	0.154
M	1	0	0	1	0	3	0.0824
G	0	0	0	4	0	19	0.0406
P6	0	0	0	0	0	0	0.005
L	0	3	0	20	0	73	0.124

*PS, positively selected sites; NS, negatively selected sites; d_n_/d_s_, average ratio between non-synonymous and synonymous substitutions for each pair of comparisons, where d_n_/d_s_ > 1 gives an indication of positive selection pressure on a gen. For the detection of positive selection, the GTR model was used with a statistical significance level (P < 0.05 for SLAC and FEL, or posterior probability > 0.9 for FUBAR).*

### Phylogenetic Analysis

**FIGURE 4:**
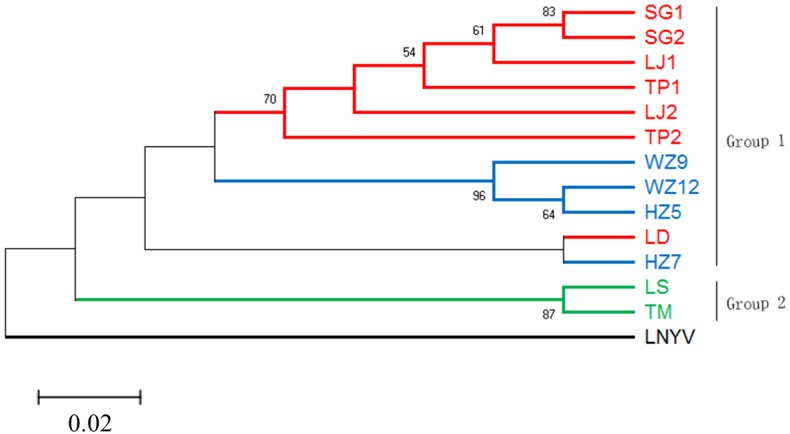
Phylogenetic tree based on the alignment of the RSMV complete genome nucleotide sequence with Lettuce necrotic yellow virus (LNYV, GenBank Acc. No. NC007642) as the outgroup. The tree was constructed using the Maximum-likelihood method with the General Time Reversible model. Different geographic origin shown in different color, and only bootstrap values greater than 50% are shown as percentage of 500 replications. The scare bar indicates the number of changes per site.

To reveal the relationship of the sequenced RSMV isolates, a phylogenetic tree was constructed using the nt complete genome sequences. The results showed that the RSMV isolates are divided into two groups based on their geographical origin; group 1 included Guangdong and Guangxi isolates, group 2 was formed by the Hainan isolates. Interestingly, the Guangxi and Hainan isolates showed little genetic distance, whereas higher divergence was observed in the Guangdong isolates, suggesting the genetic diversity among Guangdong isolates was greater than among the Guangxi and Hainan isolates (Figure 4).

## DISCUSSION

A newly discovered rice virus, RSMV, commonly occurs in the southwestern region of Guangdong (Yang et al., 2017a). Our results showed that RSMV is present in three provinces of southern China and commonly occurs in Wuzhou and Hezhou, adjacent to the Guangdong Province. This indicates that RSMV is widely distributed in southern China and it is likely to spread to other Chinese rice regions or even to regions in Vietnam adjacent to Chinese rice-growing areas. In our investigation, all rice samples showed mosaic or stripe symptoms; most of these samples (264) were infected with RSMV but the remaining samples (195) were RSMV negative. These RSMV-negative samples may have been infected with other viruses that cause mosaic or stripe symptoms, such as rice stripe virus, or may be the result of environmental factors causing the rice plants to produce virus-like symptoms.

Analysis of the genetic diversity of RSMV populations with different geographical origins can provide relevant information for understanding its genetic relationships, epidemiology, and dispersal. In our study, all RSMV isolates showed low genetic variability (less than 1% at the nt level, Table 3; and the variation rates were mostly below 1.0%, Figure 3). However, lettuce necrotic yellow virus (LNYV) was found to form two subgroups and differed significantly between them (about 20% at the nt and 4% at the aa level) based on the analysis of the N gene (Higgins et al., 2016), whereas the N gene sequence of Taro vein chlorosis virus isolates differed significantly between them (19.3% at the nt and 6.3% at the aa level) (Revill et al., 2005). This may indicate some evidence that RSMV is a “new” pathogen that emerged and dispersed recently. Additionally, the d_N_/d_S_ rate was significantly < 1 in all genes (Table 5), indicating that all genes were under negative selection pressure which was similar to that reported for coffee ringspot virus (CoRSV; Ramalho et al., 2016), orchid fleck virus (Kondo et al., 2017) and alfalfa dwarf virus (ADV; Samarfard et al., 2018). These results suggest that the insect vector may be involved in limiting RSMV genetic diversity. As is shown by the significantly lower mutation rate of aphid-transmitted cucumber mosaic virus (CMV) populations when compared with mechanically inoculated CMV populations, viral vectors can result in strong genetic bottlenecks in viral genetic diversity; this, in turn, promotes viruses that are better adjusted to plant-vector systems (Ali et al., 2006).

Most studies of the genetic diversity of plant rhabdoviruses are confined to the analysis of one or more genes, i.e., LNYV was found to form two subgroups based on the analysis of the N gene (Callaghan and Dietzgen, 2005); while CoRSV showed a strong geospatial relationship among isolates based on analysis of the N gene (Ramalho et al., 2016). Klerks et al. (2004) described the genetic diversity of strawberry crinkle virus based on the putative polymerase coding region. Pappi et al. (2016) analyzed the genetic diversity of eggplant mottled dwarf virus between seed and asexually propagated plants by sequencing 51.5% of the genome. Recently, Samarfard et al. (2018) analyzed the population diversity of ADV based on the N gene. In our study, the phylogenetic tree showed that the RSMV isolates divided into two groups (Figure 4), with isolates from different geographical sources located in different branches. However, the Guangdong Luoding isolate was most closely related to Guangxi isolates; geographically, Luoding is adjacent to Guangxi. Therefore, to understand whether the RSMV is related to their geographical origin, more RSMV isolates sequences should be added in a future study.

The base substitution types described here for RSMV are biased for transitions over transversions (Table 4), consistent with other plant viruses (Ge et al., 2007; Duffy and Holmes, 2009; Rao et al., 2017). Studies have shown that viral population genetic diversity resulting from base substitution is controlled by host-virus interactions (Schneider and Roossinck, 2001), so this bias might be caused by host preferences for viral genomic base types and base substitutions produced by the replication of the virus itself. Additionally, there are several specific deaminating enzymes in virus-infected plant cells that may affect base deamination when the viral genome is in a single-stranded state, thereby resulting in high transitions rates (van der Walt et al., 2008; Duffy and Holmes, 2009).

In plant rhabdoviruses, the P3 gene between the P and M genes encodes a viral movement protein that facilitates cell-to-cell transport (Jackson et al., 2005; Ammar et al., 2009; Mann and Dietzgen, 2014; Mann et al., 2016). The P6 gene product between the G and L genes shows RNA silencing suppressor activity (Guo et al., 2013), has been shown to be associated with virions, and may have a structural role (Huang et al., 2003). Our results showed that no amino acid mutations were observed in the P3 and P6 proteins among all RSMV isolates; this is most likely because the two genes are critical to the viral life cycle. Compared with Guangxi and Hainan isolates, Guangdong isolates show greater divergence, indicating that Guangdong isolates have a higher mutation rate. This may indicate that RSMV comes from Guangdong, although further studies are needed to confirm this hypothesis (Table 3). Notably, there are multiple specific amino acid sites in the L protein of Guangxi and Hainan isolates (Figure 2). The L protein of the negative-sense RNA virus has six conserved regions (CR): CR I, II, and IV are required for the RdRp-containing ring domain composition; CR III is involved in RNA polymerization; CR V is required for addition of the cap; and CR VI is involved in the cap methylation (Rahmeh et al., 2010; Ogino and Banerjee, 2011). The mutations among Guangxi isolates were mainly concentrated in the CR VI region and their biological functions should be examined in further in-depth studies. A previous report showed that a point mutation in CR II of the L protein encoded by the vesicular stomatitis virus induced sensitivity to high temperatures (Galloway and Wertz, 2009). Another study determined that interchange of the L polymerase protein between two strains of viral hemorrhagic septicemia virus altered temperature sensitivities *in vitro* (Kim et al., 2015). Therefore, we speculate that RSMV has adapted to environmental changes by changing the amino acids of the L protein during its dispersal; this hypothesis requires further study in the future.

Overall, we have reported the first study on the geographical distribution and occurrence of RSMV, as well as on the genetic variation of virus isolates with different geographical origins. Our results not only provide valuable molecular diversity and spatial distribution data for RSMV but also provide basic data for studying the evolutionary origin of negative-sense strand viruses.

## Author Contributions

GZ conceived and designed the experiments. XY and BC performed the experiments and wrote the draft. TZ analyzed the data and revised the manuscript. ZL and CX conducted field investigation and sample collection. All authors read and approved the final manuscript.

## Conflict of Interest Statement

The authors declare that the research was conducted in the absence of any commercial or financial relationships that could be construed as a potential conflict of interest.

## References

[B1] Ali A. Li H. Schneider W. L. Sherman D. J. Gray S. Smith D. (2006). Analysis of genetic bottlenecks during horizontal transmission of *Cucumber mosaic* virus. *J. Virol.* 80 8345–8350. 10.1128/JVI.00568-06 16912285 PMC1563891

[B2] Ammar E. D. Tsai C. W. Whitfield A. E. Redinbaugh M. G. Hogenhout S. A. (2009). Cellular and molecular aspects of rhabdovirus interactions with insect and plant hosts. *Annu. Rev. Entomol.* 54 447–468. 10.1146/annurev.ento.54.110807.090454 18793103

[B3] Bheemanahalli R. Sathishraj R. Tack J. Nalley L. L. Muthurajan R. Jagadish K. S. V. (2016). Temperature thresholds for spikelet sterility and associated warming impacts for sub-tropical rice. *Agr. Forest Meteorol.* 221 122–130. 10.1016/j.agrformet.2016.02.003

[B4] Callaghan B. Dietzgen R. G. (2005). Nucleocapsid gene variability reveals two subgroups of *Lettuce necrotic* yellows virus. *Arch. Virol.* 150 1661–1667. 10.1007/s00705-005-0528-7 15824884

[B5] Duffy S. Holmes E. C. (2009). Validation of high rates of nucleotide substitution in geminiviruses: phylogenetic evidence from East African cassava mosaic viruses. *J. Gen. Virol.* 90 1539–1547. 10.1099/vir.0.009266-0 19264617 PMC4091138

[B6] Galloway S. E. Wertz G. W. (2009). A temperature sensitive VSV identifies L protein residues that affect transcription but not replication. *Virology* 388 286–293. 10.1016/j.virol.2009.03.015 19395055 PMC2692432

[B7] Ge L. Zhang J. Zhou X. Li H. (2007). Genetic structure and population variability of tomato yellow leaf curl China virus. *J. Virol.* 81 5902–5907. 10.1128/JVI.02431-06 17376922 PMC1900275

[B8] Guo H. Y. Song X. G. Xie C. M. Huo Y. Zhang F. J. Chen X. Y. (2013). Rice yellow stunt rhabdovirus protein 6 suppresses systemic RNA silencing by blocking RDR6-mediated secondary siRNA synthesis. *Mol. Plant Microbe Interact.* 26 927–936. 10.1094/MPMI-02-13-0040-R 23634838

[B9] Higgins C. M. Chang W. L. Khan S. Tang J. Elliott C. Dietzgen R. G. (2016). Diversity and evolutionary history of *Lettuce necrotic* yellows virus in Australia and New Zealand. *Arch. Virol.* 161 269–277. 10.1007/s00705-015-2626-5 26526146

[B10] Huang Y. W. Zhao H. Luo Z. L. Chen X. Y. Fang R. X. (2003). Novel structure of the genome of Rice yellow stunt virus: identification of the gene 6-encoded virion protein. *J. Gen. Virol.* 84 2259–2264. 10.1099/vir.0.19195-0 12867659

[B11] Jackson A. O. Dietzgen R. G. Goodin M. M. Bragg J. N. Deng M. (2005). Biology of plant rhabdoviruses. *Annu. Rev. Phytopathol.* 43 623–660. 10.1146/annurev.phyto.43.011205.141136 16078897

[B12] Katoh K. Standley D. M. (2013). MAFFT multiple sequence alignment software version 7: improvements in performance and usability. *Mol. Biol. Evol.* 30 772–780. 10.1093/molbev/mst010 23329690 PMC3603318

[B13] Kim S. H. Yusuff S. Vakharia V. N. Evensen O. (2015). Interchange of L polymerase protein between two strains of viral hemorrhagic septicemia virus (VHSV) genotype IV alters temperature sensitivities in vitro. *Virus Res.* 195 203–206. 10.1016/j.virusres.2014.10.013 25456404

[B14] Klerks M. M. Lindner J. L. Vaskova D. Spak J. Thompson J. R. (2004). Detection and tentative grouping of *Strawberry crinkle* virus isolates. *Eur. J. Plant Pathol.* 110 45–52. 10.1023/B:EJPP.0000010134.06283.38

[B15] Kondo H. Hirota K. Maruyama K. Andika I. B. Suzuki N. (2017). A possible occurrence of genome reassortment among bipartite rhabdoviruses. *Virology* 508 18–25. 10.1016/j.virol.2017.04.027 28478311

[B16] Kumar S. Stecher G. Li M. Knyaz C. Tamura K. (2018). MEGA X: molecular evolutionary genetics analysis across computing platforms. *Mol. Biol. Evol.* 35 1547–1549. 10.1093/molbey/msy096 29722887 PMC5967553

[B17] Librado P. Rozas J. (2009). DnaSP v5: a software for comprehensive analysis of DNA polymorphism data. *Bioinformatics* 25 1451–1452. 10.1093/bioinformatics/btp187 19346325

[B18] Mann K. S. Bejerman N. Johnson K. N. Dietzgen R. G. (2016). Cytorhabdovirus P3 genes encode 30K-like cell-to-cell movement proteins. *Virology* 489 20–33. 10.1016/j.virol.2016.01.014 26700068

[B19] Mann K. S. Dietzgen R. G. (2014). Plant rhabdoviruses: new insights and research needs in the interplay of negative-strand RNA viruses with plant and insect hosts. *Arch. Virol.* 159 1889–1900. 10.1007/s00705-014-2029-z 24610553

[B20] Martin D. P. Murrell B. Golden M. Khoosal A. Muhire B. (2015). RDP4: detection and analysis of recombination patterns in virus genomes. *Virus Evol.* 1:vev003. 10.1093/ve/vev003 27774277 PMC5014473

[B21] Ogino T. Banerjee A. K. (2011). An unconventional pathway of mRNA cap formation by vesiculoviruses. *Virus Res.* 162 100–109. 10.1016/j.virusres.2011.09.012 21945214 PMC3221763

[B22] Pappi P. G. Maliogka V. I. Amoutzias G. D. Katis N. I. (2016). Genetic variation of eggplant mottled dwarf virus from annual and perennial plant hosts. *Arch. Virol.* 161 631–639. 10.1007/s00705-015-2705-7 26660163

[B23] Rahmeh A. A. Schenk A. D. Danek E. I. Kranzusch P. J. Liang B. Walz T. (2010). Molecular architecture of the vesicular stomatitis virus RNA polymerase. *Proc. Natl. Acad. Sci. U.S.A.* 107 20075–20080. 10.1073/pnas.1013559107 21041632 PMC2993402

[B24] Ramalho T. O. Figueira A. R. Wang R. Jones O. Harris L. E. Goodin M. M. (2016). Detection and survey of coffee ringspot virus in Brazil. *Arch. Virol.* 161 335–343. 10.1007/s00705-015-2663-0 26553342

[B25] Rao L. X. Guo Y. Zhang L. L. Zhou X. P. Hong J. Wu J. X. (2017). Genetic variation and population structure of Cucumber green mottle mosaic virus. *Arch. Virol.* 162 1159–1168. 10.1007/s00705-016-3207-y 28054163

[B26] Revill P. Trinh X. Dale J. Harding R. (2005). Taro vein chlorosis virus: characterization and variability of a new nucleorhabdovirus. *J. Gen. Virol.* 86 491–499. 10.1099/vir.0.80591-0 15659770

[B27] Samarfard S. Bejerman N. E. Dietzgen R. G. (2018). Distribution and genetic variability of alfalfa dwarf virus, a cytorhabdovirus associated with alfalfa dwarf disease in Argentina. *Virus Genes* 54 612–615. 10.1007/s11262-018-1563-2 29730762

[B28] Schneider W. L. Roossinck M. J. (2001). Genetic diversity in RNA virus quasispecies is controlled by host-virus interactions. *J. Virol.* 75 6566–6571. 10.1128/JVI.75.14.6566-6571.2001 11413324 PMC114380

[B29] Uehara-Ichiki T. Shiba T. Matsukura K. Ueno T. Hirae M. Sasaya T. (2013). Detection and diagnosis of rice-infecting viruses. *Front. Microbiol.* 4:289. 10.3389/fmicb.2013.00289 24130554 PMC3793123

[B30] van der Walt E. Martin D. P. Varsani A. Polston J. E. Rybicki E. P. (2008). Experimental observations of rapid *Maize streak* virus evolution reveal a strand-specific nucleotide substitution bias. *Virol. J.* 5:104. 10.1186/1743-422X-5-104 18816368 PMC2572610

[B31] Weaver S. Shank S. D. Spielman S. J. Li M. Muse S. V. Kosakovsky Pond S. L. (2018). Datamonkey 2.0: a modern web application for characterizing selective and other evolutionary processes. *Mol. Biol. Evol.* 35 773–777. 10.1093/molbev/msx335 29301006 PMC5850112

[B32] Yang X. Huang J. Liu C. Chen B. Zhang T. Zhou G. (2017a). Rice stripe mosaic virus, a novel cytorhabdovirus infecting rice via leafhopper transmission. *Front. Microbiol.* 7:2140. 10.3389/fmicb.2016.02140 28101087 PMC5210121

[B33] Yang X. Zhang T. Chen B. Zhou G. (2017b). Transmission biology of rice stripe mosaic virus by an efficient insect vector *Recilia dorsalis* (Hemiptera: Cicadellidae). *Front. Microbiol.* 8:2457. 10.3389/fmicb.2017.02457 29312171 PMC5732235

