# Supplementary material for: Geographic Distribution and Genetic Diversity of Rice Stripe Mosaic Virus in Southern China
Source: Front Microbiol. 2018 Dec 10;9:3068. doi: 10.3389/fmicb.2018.03068 (PMC6295562; doi:10.3389/fmicb.2018.03068)
Supplement: Supplementary file 1 [file Table_1.DOCX]

**Table S1. Nucleotide identities (%) of the untranslated regions between each gene of RSMV isolates from southern China**

| Provence origin | RSMV isolates | 3‘-N | N-P | P-P3 | P3-M | M-G | G-P6 | P6-L | L-5' |
| --- | --- | --- | --- | --- | --- | --- | --- | --- | --- |
|  |  |  |  |  |  |  |  |  |  |
| GD | LD |  |  |  |  |  |  |  |  |
|  | TP1 | 100 | 97.1 | 99.5 | 97.4 | 100 | 100 | 98.6 | 100 |
|  | TP2 | 100 | 98.1 | 99.5 | 97.9 | 100 | 100 | 98.6 | 99.7 |
|  | LJ1 | 98.9 | 98.1 | 99 | 97.4 | 98.3 | 100 | 98.6 | 100 |
|  | LJ2 | 98.9 | 98.1 | 99.5 | 97.4 | 100 | 100 | 98.6 | 99.7 |
|  | SG1 | 98.9 | 98.1 | 99 | 97.9 | 100 | 100 | 98.6 | 100 |
|  | SG2 | 98.9 | 98.1 | 99 | 97.9 | 100 | 100 | 98.6 | 100 |
| GX | WZ9 | 100 | 98.1 | 99 | 96.8 | 99.2 | 100 | 98.6 | 100 |
|  | WZ12 | 100 | 98.1 | 99 | 96.8 | 99.2 | 100 | 98.6 | 100 |
|  | HZ5 | 100 | 98.1 | 99 | 96.8 | 99.2 | 100 | 98.6 | 100 |
|  | HZ7 | 100 | 98.1 | 99 | 96.8 | 99.2 | 100 | 98.6 | 100 |
| HN | LS | 100 | 96.1 | 99.5 | 97.4 | 100 | 100 | 97.2 | 99.3 |
|  | TM | 100 | 96.1 | 99.5 | 97.4 | 100 | 100 | 97.2 | 99.3 |
