# Supplementary material for: Geographic Distribution and Genetic Diversity of Rice Stripe Mosaic Virus in Southern China
Source: Front Microbiol. 2018 Dec 10;9:3068. doi: 10.3389/fmicb.2018.03068 (PMC6295562; doi:10.3389/fmicb.2018.03068)
Supplement: Supplementary file 3 [file Table_3.DOCX]

**Table S3. Nucleotide (top right) and amino acid (bottom left) identities (%) of RSMV P genes among isolates from southern China**

| Provence origin | RSMV isolates | RSMV isolates | | | | | | | | | | | | |
| --- | --- | --- | --- | --- | --- | --- | --- | --- | --- | --- | --- | --- | --- | --- |
|  |  | LD | TP1 | TP2 | LJ1 | LJ2 | SG1 | SG2 | WZ9 | WZ12 | HZ5 | HZ7 | LS | TM |
| GD | LD |  | 99.7 | 99.4 | 99.4 | 99.2 | 99.1 | 99.1 | 99.6 | 99.6 | 99.6 | 99.6 | 99.5 | 99.5 |
|  | TP1 | 100 |  | 99.6 | 99.6 | 99.5 | 99.4 | 99.4 | 99.8 | 99.8 | 99.8 | 99.8 | 99.7 | 99.7 |
|  | TP2 | 99.5 | 99.5 |  | 99.3 | 99.1 | 99 | 99 | 99.5 | 99.5 | 99.5 | 99.5 | 99.4 | 99.4 |
|  | LJ1 | 99.7 | 99.7 | 99.2 |  | 99.1 | 99 | 99 | 99.5 | 99.5 | 99.5 | 99.5 | 99.4 | 99.4 |
|  | LJ2 | 100 | 100 | 99.5 | 99.7 |  | 98.9 | 98.9 | 99.3 | 99.3 | 99.3 | 99.3 | 99.2 | 99.2 |
|  | SG1 | 99.5 | 99.5 | 98.9 | 99.2 | 99.5 |  | 99.1 | 99.2 | 99.2 | 99.2 | 99.2 | 99.1 | 99.1 |
|  | SG2 | 99.7 | 99.7 | 99.2 | 99.5 | 99.7 | 99.7 |  | 99.2 | 99.2 | 99.2 | 99.2 | 99.1 | 99.1 |
| GX | WZ9 | 100 | 100 | 99.5 | 99.7 | 100 | 99.5 | 99.7 |  | 100 | 100 | 100 | 99.6 | 99.6 |
|  | WZ12 | 100 | 100 | 99.5 | 99.7 | 100 | 99.5 | 99.7 | 100 |  | 100 | 100 | 99.6 | 99.6 |
|  | HZ5 | 100 | 100 | 99.5 | 99.7 | 100 | 99.5 | 99.7 | 100 | 100 |  | 99.7 | 99.6 | 99.6 |
|  | HZ7 | 100 | 100 | 99.5 | 99.7 | 100 | 99.5 | 99.7 | 100 | 100 | 100 |  | 99.6 | 99.6 |
| HN | LS | 100 | 100 | 99.5 | 99.7 | 100 | 99.5 | 99.7 | 100 | 100 | 100 | 100 |  | 100 |
|  | TM | 100 | 100 | 99.5 | 99.7 | 100 | 99.5 | 99.7 | 100 | 100 | 100 | 100 | 100 |  |
