# Supplementary material for: Geographic Distribution and Genetic Diversity of Rice Stripe Mosaic Virus in Southern China
Source: Front Microbiol. 2018 Dec 10;9:3068. doi: 10.3389/fmicb.2018.03068 (PMC6295562; doi:10.3389/fmicb.2018.03068)
Supplement: Supplementary file 7 [file Table_7.DOCX]

**S1 | 12 RSMV sequences described in this study.**

>Seq1 Rice stripe mosaic virus TP isolate 1, complete genome

AAGGAAGTTGCGTTGCGAACGCAACATTAAAATGTTGACATAACCCACCTTATCATAACAAAAGAAAAACGAGACCGCCAAGTCTTAAAATGGCAACCGACAAGTCTTTTGAGGAGAAGCTGAGCCTGGTTCCTGAGAACACTAAGCTATATTCAATATCTCCTGAGGCATATTCAGATGATAAGTTTGATAAGGCCAATTGCTACAAGCTAGAGAAGAGGTCTGAATATGAGCTAACTAGGCTCTACAAAGGACTAGTCCGGGACTTAGGGAATTCTTCCCCCTCCACTTATGCAGTCGAGAGATTGCTAGTCTTGGCAAGCCATCTGTATGAGACCAAGAAAGGGTCTTCTAACTTCTTCTTGACCGATTACCTGCCCAAGACAACATCAACAGCAAATCTTGATGCAGGCTTTTTGGCAAAGCTGAAAGAGACCCCTAAGGCCTCTGACCCCGATGTTTCTGATGTGACAGAAGTCAAGACAGCTAAGGCTACTCTAGACTCTGCTACAACTGATGCGGATACTAAAAAGGCCGCTTATGAGGCTATCGGAGATGAGGATTCAAAGAAGGCTGAGAAGGCTACAGCAAACACTGCCTGGATTGCTGCCCAAGAGGCCCAGAAGAAGGCTCAGAGTGCCTATGATAAGGCAGTTTCGAATGCGAAGAAGGCCTCAAGGAAAACTACCTCTGGAAAAAGCTTGTTCGGGGATGCAGGGGAGACAGTCACAGATGAGTCTAAGGTAGTGGAAGAGGTTGGAGAGGGGAAGAAGAAGTTTGGACCCTTCTTAGCTGCTTACTTGATGAGGCTACTGACCAAAATAGCATCCAATGTCACAGAGTCATGGGAGCATATGAAAGGAATGTATAAGAACTTTTATGGTTATGATGCTCCTTCAGACCTGAACTGTCCCGAAGCAGGATTCCTTGAGCAACTGAAGTCTGAGCTTAACAAGGATAGAAGGGCGGCTACCTCATGGGTCAAAATAGTAGCAGAGGCAGATAATAAGTTGGATCAATCTACAGCCGAGGCTGGGATTCTCCGCTATGTGGCTGTCCTCCCTCTTGCATATTCTGGGATGCATGCGATGAAGTTGTTCATGGACGTCAAGATGCTGACCAAACTCACCAGCAACTACTTGATTGGTGCCATGAGGAGCCCTCTGACCAAGGATGCATTAGATGCAATCATGGATATCTTGATCTCGTTTGAGTCCACTACAAAGACTAAGAAGTCGGAGAAGTTCCGGTTTGCTAGGATTGTGAGTACTCAATTCTTTCAATCCCTCCAGACCAAGAATTGCAAAGAGCTGGTCTATCTGATGGTTCAGATAATTGCTGAGTACAGGAAAGCAGAGGGGGTCAGGGATCCCATGAACATTGCTGGATTAGATGACATCTCTAGCAGGAACAAGAAGAAGTTGAATAAAGCAGTACGGATAATACTGGCAGAAGCTCCAAAGGCATCCGCAGGTGAGTATTCGTCAGCCATGAAGAAGGCTTTCCTGGATGACGAAGAGGATGATACAGCCAAGACCAGGTCCATCTTCCAGACCAAGGCTTAAGGAACATCATGTTTGGATAGAATATGACTCCTCTAAATAACTATGCAGCCTACTGGCGAGTCTTTGTGTGTTATATAAGAAAAACGAGACCGTCAGGTCAATAATGAGTGTGCCAGAGGATACTCCCTTCAGATCGTACTCCAGCATCTTTGACGACTCAGACTTTGTCCAACCACAGCCCATGTCCTTCAAAGCGACCAAGGAGTCAGAGAGCCTTCCTGAGACAGAGAAAGAAGATATGTCGACTGAATACCTCTCTGAACCACTGAGGACCAAGTCAGGTAAGAAGAACAGACGGAGGAAGGGAAAGGACCTAAAATCTCTCTTCACTCAAGAAGCTGGACTCCCGGCTCCAGAAGCAGACAGTGTCCTCCCCGAATCGTCTCCGTATGAGAATGATAACGCACAGTTGGAGCTACCCAAGCCCATTCTAAAGACATCAGACGCTCCGGTCTTCCTAAGAGAGAAGGATCTTAGTAAAGAATTTGCTGCAGCCTGCAAGACTAATGGGATTCTCCCAAGGGATGAATGGAAGTCATCTGTAGCAGCCAAGTATCATGCAGAGGAAGGGAAAATGACCAAACGAGACATCTCGTTAATCGTATTTGGAATGGAGCTTTACAAGAGATACAATGTGGAATCTGAGGTATCAACTTTGTTCACTTCATTGGTGACTGAGTTGCAGGGGATAAAGGTTGCTGCTAAGGAGTTGAATGATACCCGGGAGGTCCTCACTAAGATTCCAGGAGAAATTGTGTCTGCTGTCAAGGCAGGGGTAAAGGAGGGGACCGAGATGGGGATGGATTATATAGAGACTAGAACTAAAGTGGCCCCCAAGAGTGCTCCTAAAGTGGACATCTCCAAGCCGATGAGTAGTAAGATGATGGAGCAGCAGGATGAGAGTTCTGATGAGTCCTCAGATAATGAGAGTGAGGAGAGTGAGGAAGAATCCTTTGAGACAAAGGCTGCAATCTTCTTAGCTTTGATAAAGGTTCCAGAGGAAGAGAGAGACAACGCAATAGTCCTTATGGCCTTGAGGGCAGTGATATCTGACAGTGAGCTGAATCAGGCAATTAGGAATGACAGAATCTCCTCCTCAGTAGCAGATATGTACCATCAAAAGATATCTGACAAGGCTAGGGAATTGATGGGAAAGGGAAAGACCAACAAGAGGGCCAAGCAGCCTAAATCCTCTAAGTATGCATCGGATTACTATGATGATGCACTGTGAGTAATCAGTTACTACCTGTGTGGCAATGTCGGAACTGTACTTACCTATATATTGATAATCGTCTGTGATCTTGTGCCTTTAATTGCTGCAGTATTATAGCTTAAAATAATCTAGTTACCAATGCTCTATTTTCGTACTTAAGTGCCATGTTGCCTGCCTTTACCAAAGTAATCTAGTGTGCTCTATTTAAGAAAAACGAGACCTTCATCATGAAGATCATCTGCAGTACTGGGATCTTCAATGAAGAGAAAGGCTTCCCCCTCCCCAACCTCCTCAACAGTCCCTTGATGAAGCAGGAGATCATGACGGTCAAGTACTTGAGGTTTCAGTATATCCCTATTATGACCAGCAACCCCTCGAGTTCATTGACTATTGACATTAAAGATACTCGACTGGTCAATTGGGATAATAGGTCGATTTTCCAGGTCAAAATATTCGGGGATGTTCAAAGCTCATTTATTGTATCGGGTCTACAGCCTTATTCAGCTAGAGACCGTTGCCCTTATCTCCTCTCCCTTTCAGTAAACGCAGGGAAGGTTGTTCCAGGGACCAAATACGGCATCTTAAAATCTTATGCTGTGTACACATCTAAAGACTCAGGGATAATTTCGTCACAGATCTCTGTAAAACTTGAAAGGTCCCCACGTGACTACTTCTTGAAGAGGTCTAAGGAGCATGACAAAAAAGATCTTGACAGTGATGTCTCATTCAAGATGTGTCGCCATGTCAAGTTTGCTACTTGAAGCTTGGAGAGGATGGGCAGCTGAATGTTGTCGGGGTGATGTTTTCCTTATGAATATCTTCAATAATTTCCCTGGAGGGAGAAACAGTTGATCTCTGGGAGGACGCAAGGGCTCACTACTAGCATGTGCTTGCCAGTTTGAGGTTTTATGCCTATCATGGTTTAGCCTAAATAAGAAAAACGAGACCATCATGGCCGTTCCGTGGACTGAGGTTAAAGACTCCAAGTACTTGGCAACTAAGATGTCTGTTACCCTGATCATGGAGATGAATGAAGATACACCTCTAAAGTATCCTTCTTACAACGCATTCGAGAGCATCTTTAAAAGGCTAGCAGAACCAGAAAGCGCAGCACCCCAAGTGGCCGCATGGTTTACTTGGTTCCTAAGAGAGGCAAAGGACATCTACTATCTGGAAGTGAGTAACAAGGAAACTGCCCAATACGGACCAACTAAAGTCTATAAACTTCAGTGTCCAGCCTACCTCTTATCCCGAGTGACAGGAGGATCACAGCTTGATTATACCTCTCTTATAGGTAGCAAGGTGATGACTGAGAAAGATCGGGGGATCCCAGTCAGAACACTCTATATTACTGGAGGGGGTACATCATTCAGAGTCATCAACGAGGAGACAGCCAACCAGTTCATCATAAATGATAATGCTGTCCGCCTACCAGGAGAGTGCAAGGTGGATGGAGGAAGTATAATCTGGAGTTAGAATTTCAAGAAAACTAAACAATAATGAACAAGTGGAACAATCTCGTGTAGTGTATTAGGTGTCCTCTTTAACTAAAGTTAAATAAGAAAAACGAGACCCGCAAGGATCTTAAGATAACAAGATGATGAGGATTTCGGTCTTTCTCTTGATACTCTGCTGGCTCCCTGTCAGCCTGACCTTCTTCGACAAATCACACATTCCTATAACCACATGTGATAAGAATCTAATGAGTCCTATCCCTTGGAGAACTTACTGCATAGAGGAGTGTGGAATCCGGAATGTAATAGGTGATAAGCTGGATCTGTTTATCTACAATAGGTCTGACAGTGGGAAAGTACAGCTGGCTGACTGCAGGAAGTACAAGATCAGACAGACCTTCACCAAGACTTGGACATTCTCGACATTCAAAGGGGCGATAGAGACAGAGGAGCTAATGCCTAATTATGCAGAGTGTGAATCCACCTGGAGAGATCTGTGCAACTCTGGACCGTGTAGTACCACAACTCCGGTGATCCCTGAGGAATACCATTGGGCTTCTGACACCACGAAGGAGGTCATCTATGTATCTATAGATGCATACCAGAAAACTGTTGCATTCCAGGATCCTAGTGGTGATATCCAGCTCCTAGTCCATGGGGTGATCATAGATGGGAGCCAGTCTGGTTATGTTCAACCCAGCAAAGATCTCATCACTATGTGGGATAAGGTTGAGTTACAAGATGAATGCCCTTGGTCAACGGGAAATTCTCTTTCGTGTTCTACGTCTGATGAGGGAATTTCATACTACTGTGCTGGGAAAGGGCTAGTACTGACCAACATCAGTACGGTGACTGATACCAGATGTGACAACAACCCGCACCTAATGACATCAGGGCACCATGTGATTTTTAGAGTGAAGAAGGCATCAGACCCGAATGCGACTCTCAGCAGGACAGCTCAAATAGTGCTGGACAGGGGGTCAGAAGAGGCCGAGATTGTAGATAGTGTTAATAAGGCGTTGCTGGATAGAGATTCCATCAGGTGTGCAAGCTCATGTCTCGCCTTTGATTACACCATCTCCAAGCCTCAGATGTTTGGCAACCAATTGGCGCTACCTTATAAGGGGTCTTTTCTCCCTTGCAACATACTGCCTAATTGTCGGGTTGTCTTCCCAGTCAAGTATTGCAGCTCTCCTCCGATGATTCTGGTAGAATGTACCGGCACTATGACATGGTGGAATATCACTGGAGATTACACGATCAGACCCACCTATTGCCACATGAACCAGTCGGCGACCAAGATTAAGACATCTATATCCTTTATGACAACAAATGGGAGAGTCTTAGTGAATGAGTCTGGCGCTTATCCTGTCTCCCGTGAAATAGGAAATACGTTCCAGGTCGGACATGTCATAGAGCCTAGCTCCATGATAGAGGTGACTGATCCACTTAATGTTAGGATAGATGACACCTTAGTCACACCAGAGTCCCATACTATATCTAATATCACTTCAGTCGGGGACTCACTTTTGGATACAATGGTTGAGACCGTGAAAGGTATCGGTCGCTTCATATCCCATGAGGTCAGGATAGTGGTTTTTGGTGTTCTGACTCTTTTTATATTATATCTGTCGTTTAAATATTTGTTCGCTAAGAAGAAGAGCAGAGTGCCGCACCCTAAAGTTGTCTATACAAAACCCACATCTGAAGGACCAGTGATCTATGACACCGAATATACTATAGAAAGTGACTAATAAAAAACAGAGACCAACATGGAGTTCAATTGGCCTTGGGGACAGAACAGTGAAACGGAAATCACCAAGAATCTCCGCTTTGAGGACATTAAGGTGATGGCCATAATAATACTAGTCTGGGTGAAGTGTCTTCTCATCTACCATTTCAAGAGGAAAATAAGGCGACTAAGATCTCTATTGATAAAAGGATCCTCACAATGGGTACTGCATGATGCCTAACTCATTAGGAAGATCTGAGATGAGTTTTTATCCTGCCTTATCCTTCTAATTAAGAAAAACGAGACCGCCATCATGGACCTTGATGACGGTGGTCTATGGAGACGTGCTAGGGGTCTGGGGGATTATCACCTGAGGTCTGCTCTGGTGACCCCCTCCTTAGAGCGTTTCCGCAGTCGAAAGGGAAGGCACCGTGAACAACTGTGCTTTGATAGGATGAAATCACTAGGCTGGATGTTGAGGTGGGTAGATCAAGGGAAACTGCTTGGATATCTAATGGTAGAAGCCAACAAATCTTTACCAAAATCCATTGCAAACCAGGAACTCCTAGTTGAGACTCTAAAGTTAGAATACGGATGTCTTAGACAGATAATCATGACGGATGGAGACCTCCATGATCAGGTGATCTCTTATCTAGACAGGAAATCTATCTCTACCCATTACACCCATGGTAGGGAGGTCTTTCAGGAAGCCTTGATAGTTGTCATGGCACTTTCTTCAGGGAGAGAACCACCAGATCATGTCAATAACTTGGGTTGTGAAATGCTGAATGAAGAACTTGAAGTGCCCGTGGTCAGAACCTATGGCGTAATCTTCTATCTCTTTGGGGACTTGATCTACGTAAAGTATCCGGAGGAAGAGGGCATGATCACACTGGACATGTTCAGAAACCTGACAGATAAATTCTCTGAGAGGGAGAATATTATGATTGCAACTCAACTAGGAACTGAGATCCTTCAAGAGATATACCCTTCTGAGACAGTGCTCAAAACAGTCTTTTCTCTTTGGGACAAAGGTCTATTGAAAGAGGGAAATGACTTTTACACAGTGGTAAAGACATTCGAAGCAATCATAAATGGGATGTTGATCAAGAACAATGACGGAACATACTATGATCCTTCAGCATACCTCCGAGAAACTATCATGGGGCTGCCAGTACGACTGAGGGATTATGCACAGACGTTAGTGAGTTACTTAGACTCTCTCCCCTTTAATCCACACCACCTATCTCAGATTGGGGGGTTGTTTCGGTTGTGGGGACACCCCATAGTAGATCCTAACGCTGGGGTCAGAAAGGTAAGGCTTCTGGGAACAGCTGATAAGATGAACCTGACACATATCCCCACCCTAGCAGAGCGGAAATTCAAGGAGATCTTCTACCTGTCGTATTATGAAAAGCATAGAGTCTATCCTAATCATAACTTGAATGGAGAAATTGAAGGAAGCTATTTGCTCTCTCAGCTTGCACAAATGGCTCTGGTTAATCCCAAGCATGCAAATTACTCACTAGTAGACTGGGATTCAGTCAACACACTAGAGACCTTTCCCTTTCCTAAATCATTCAACCTGTCTCTTATTATAGCGGATAAAGCTGTAAGTCCGAACAGAGAAGAATGGTTGGAATTGAGACGTAAGGGAGGAACTCAGATGGATCCGCACATTAGAAGAGGGCCTCTGAAGGCTATGAAAGACGGAGTAATCGATTGTGAGAAACTATTGAGGAAGATAGACCGTAACCCGTCCGGTTTAGCTAAGAAACATAGGATCATTGGACTGTACCCAAAGGAGAGAGAAGAAAATATGGTTCCCAGGATGTTTGCATTAATGTCCTTTGACATGAGAGCTTTCTCAGTGGTCTCTGAATCCATGATAGCAGATCACATCATTCCTCATATAGAAGGTGTCACAATGACGAAGAGCATGTTGGCCCTCCAGAAAGAGATGATAATTTCAACTAAAAGCCAGGCCTCCTCAACCCAGTCAGACAGTATAACCTTCTGTCTTAATATTGACTTTGAAAAGTGGAACCTTAACTTCCGAAGATGGATGACGGAGGGGGTATTCAGAGAAATGGGGCGATTGTTCGGACTTCCAGAGATATTCAATCGGACTTATGACATTTTCAAGAAATCCATCATTTATCTAGCAGATGGAAGCTTCGACCTCTTGCTGACAGATGAGTTGGAAATAGAACCAGGGACCAATCCAGATTGCGCATACACCGGTCATGTTGGGGGGTTTGAGGGATTGAGGCAGAAAGGATGGACAGTCTTCACAGCGGTCTTAATCTCTTCCATCTGCGATGAAATGGGGATCAAGACTCATCTAATGGGGCAAGGTGATAACCAGGTTTTGATGCTGACCATCTACTCAAGAGCTGCTAGAGAGACGGGGGATTTGAAATCAGCACCAGCAGTACTAGAGATCACAAATACATTGGAGACATTCAAGAGCAGACTAGTCTCACTCTTTGCTAATCTTGGATTGCCTATAAAACCACTAGAGACCTGGGTCTCAGAGGAATTGTTTGCCTATGGCAAGACTCCAATATACCGAGCAGTCCCCCTTGCAATGAGTCTCAAGCGAATCTCTAGAGTTTTTGCGTTTTCCAATGAGGATCTAATGACCTTGTCTAATGCGTTAGGTGCTATCTCTGCGAATGCACAAGCTGCATCCATGTGCGATGTCCATCCGATGGTGTCATATGCCATAGCAAAGTGGCAACACCTATGCTGTGCGATAATCTTCTCCAACTATCATCCGCTATGCGGGTGTGCTCCACATGTCTCAGGAGAGGAATGGGCAATAAAGCTACGACTTCCGTCAGGAAAGAAGATTCAAGAGACATCTGATGAGGAGATAGACGAAAGAGATCTGATGAAACTAATAGTGACCATACCTCGAAGCTTAGGAGGTTATAATACCCTGACTCTGTATGAGATGATCATGAGAGGATTTTCTGACCCAGTCTCTAGGGACATGTGCTGGCTATTCGCAATTGCCAGTGAATCAACAGGAAAACTTCGAGGTTATCTGATAAACTGGATTAAACCAATCGTGTCTCCGGAAGTAAATGCTCAGCATCTCATTCAGGATCCCACGGCACTCAATTTGCTAGTACCCCCTAATGCTACATCAGTTATTAAGAGGATGATAGACAAATCACTCGAAGCACTCCCAAAGAGATCTCAGTTCGCAGTATGGTTCTCTGAGATCCTAGAGATCTCAGGGGACAAGGAAATCTCAAAGTTAGCAGAGGCCTTGACCAGGACAGATTCCCTGAACCCCCGTTTCCTTCATGACATTCTGGGGGCCACTCTTTATGGATACTGCACGGCTATAACTAGTAAAGTTGACAAGACAGTGACCTTATCTCGGATGGCACTAGCGTCTAAGGATGTTGTTGGAGCTCTAATCAAGGGGGAGATGAGACTTTACTCCTATTTCGGATGGAGAACCTTACAATCGAGAGGCTTACCTCTGACCACCAGATGTCCCAATAAGTGGGTGCGGATAATTAGGGATATCTCATGGCAAAAACAAATTAAGGCTGTTAGCGTTCCGTATCCTACTCATTTCTTATCAGAGGATATTTCTGAGACTGATAGGCCCGATTCCTGGATTGAGTGCTATATTGATGATGCCCCGACATCTGACAGAAGTTGCTTGATCTATTCGACAGGAAAAGCTCTGCCGTACTTGGGTAGTGTCACTAGAGAAAAACTGACAACTCGAGGTGCAAAGGCGGCCTATGGAACTGAACCTCTTGTATTGAGGCCAATTAATCTAGTCAGAACTATAGGTTGGTTCATTGAAGAGGATAGCAATTTTGCTGAATTGATCAAGATGCTACTGGGAGCCGTCACAGATCTTCCCATAGAGGAGGTACTCTATATCCCAGAAATGGTGAGTGGCTCTATGGCACATAGATACCTAGATATGTCAACACAGCATGGATCTTTGTGGATGCCTCTTTATGGTCCTGCCACTTTTCTCCATATGAGCACTAATACTTTTGTGCAGTACCTGAAGGGGACAGAAAATGTCACTTTGCACTTTCAGTGTGTCATGGGCTTAATTCAGTATGCTATAGTCAACAAAGCTCTAGGGGAATGTCCCACAAAGAGAATGACGAGATTCTTCCGATCATGCCCTGACTGTATAGTTCCCATTGATGATACGTTGGAGGACTTGCCTGAGGTCCCTTCGCTAGATCTCATCCCTGAGAGGACCACAAACCCTTATCTCTACCTCAAGAAGGAGAAGATTGAACTGAATGTTAGGCATCGACTTGCAGTGATAAATGAGATCCGGGTAATAGCTAGGGATGAGATAGAAGAGACTCCCCTGTTGGCTAACAATACTCTGGAGGACGTGATGAGTTTGAGGGCAGCTCAGAGAATATTCTACACAGCCAAAGGAAAGGAGGCACAGTGGGACTTGCAGACTGCTGATCGAGAGGGGTACTTAAAACTGGATTTCGTGAGCGTAATCAGGAAGATTATAGGACACCTCTTTGTAATGGAATCAGAGACGTTAAAAGCCGGGTCAGACTACCCTACTTTCCAGTTACAGCAGAGAAGGATCATGCGGCGCGTTAGGCAGTCAGATACATCTAACTTTGTCCACTTAGGAGGGTTCTTCTGCTGGCAAGAGAGCATTGCGAGGATACAGAAACTGAAGTGGTCAGTGATGCCTGCCACATTCCCTATCACTGCAGAATCAGTGTCGCTGGCAGCTAAGATGAGTCTAATAGGGGCAATGACTAGCGGGCTAACACCTAAGAGATGTGATGGTGTTCTGGCCGAAAACTTAATCCCGGATGTCACAAGGCAAGCAAAGAACATCATATGCCTGGACAAAGTGTTCCAAAGCAGATGTGATTACTGCTATACTGCAGCAATGACAAATAGGTGGTCAGACTCGATAAACTCCGACAGCATATTCAATATGAGATGTGAGAGAGGCCATACCATTCTGTCCCCGAGAATGTTGCATCAATTGAGGCGAACTATCTTGCCTGAAGGAGCCCTATACACTTTGGCTGTCAGAGTGGTTCACCAAAGCCCTCCTGAAGCATCACAACCTCTTCAGATCATCCCATGCAGGCGAGAGAGATATCAAATCTTGAGTGAAGCTGACATGCCTAGACAAACTGGCTACTCGGGGGTCTATAGAAGGCCTACCAATCAAATCTGCATGTATACAGAGGTTGAATTGGCCAAGAAGTACAGGTTGCCAACTAATTCATTATACAGGATCCTTGACTTACACGACTGCTTCTTCAGTCAGAAACTCATGGACACAAGGGGCAACATTTTAGTGGTCGGAGACGGGTATGGATACAGCTCCTTGCTAACCAAGTGCTTGAACCCAGACAGGAATGTAGTGAGCTGGACCTATATTGAACCATCTGAGGCACTTCCACATAGCCTGAGAATCTCTAAGCCTCCAATGCACTACAAAGCTGATGTGCAGATAGATTCTAGCCCTTCAATTGACAGAATCTCCGACATACATAACTCCTCATACCCGGATGAGTTCGCTAAAGTGGTAACCAAGAATTGGATTACAGCACTGATCAGTGACATTGAAACTGTGTATGTCTCTGGAGAGAAGAGTGTGTCGTCTCTGATCAACTTGGCTTGGAATAACCAGATCCAATTAGGAGCACTCAAGTTGGAATTGATGACAGATCCCTTGGAGAAAGTCGTAGAATATGCCCACAATGCTTACCAAAGATGGGAATTATTCACTCTACCAGGCGCCAATCTCGGAGGAGGGGTACTATACATAGGTTTCTACGGTAGAAGAGAGAAGTTGACTGGGTACATCATTCCCCATTCAGGAGTTGAGACCTTGATGGATAGGTTGGCAACTGAGGTAGATGAGGATAGAGGAAGATTGAGAGCAGAGGACCGTGATCGGTGGGAGCAGTTGGATACAATGGAAAGCAAAATACATCTGCAGCAATACCATAAGATGAGGCTCGATCTATGGTTCGGATCTGCTTATCTGAGTGGTTATCTCTCAGAGGATATGACTGAGTTCTTTTACTCGGTAAAAACATCCTACAGACCACCTGCAATCTCATGGGAGAAAGGGAACCAGGCCAGATACCTCTATGGCAGCAGGGAAGAGATCTTATTCATGAGCATGATCACAGTGGCACTATCATCCTATCTAGAGGACCAGGATGTCTTAAGAGAGTTCCTTTCTTCCTCAGGGTGGAAACTCCAGTGGAAGAAGGATAAGAAGAATCCGAGGCATTGGTCCCCGTATTTGGAGCGGAGTGACTCTCAACTAGTCTGGAAAAAGAACAAAGCCATGATTTTCAAACTGGTATCAGTTCTGAGAGGATCAAAGCCGAGAGAAGAATATAAGATGGGAAAAGCATCTGACTCCATAGTTTTCAGATACATTCCGAGGAAGGTAAGGGAAGGAGAAGTTCCCCTGTGCTTCCCGATCTCTAAGGTGGCCTCAATGTCGGTATAAGAAAAACGAGACCTTCATCATGGATGATTTAATGGTCTATAGCCTTTGGGCTCTGCTGCAATGGGGTTTGCCTGCGGATACAGCCAGATACATAATCTCACTGAGGATGGAAGAAGACTACTGGGAAGAGATGAATGAACATGAGCAGTGGGACGGGTACGAGTCTTGGGATGATACTGATTGGCAGTATCCGAGTCCTTGAATAAGTCAGTATGTGACAATTTAGAAAAAACGATTACCTTACTGTTGACTTTAAAGAACACTGTTATGTTGTGTTCGCAACACACAACTTCCTT

>Seq2 Rice stripe mosaic virus TP isolate 2, complete genome

AAGGAAGTTGCGTTGCGAACGCAACATTAAAATGTTGACATAACCCACCTTATCATAACAAAAGAAAAACGAGACCGCCAAGTCTTAAAATGGCAACCGACAAGTCTTTTGAGGAGAAGCTGAGCCTGGTTCCTGAGAACACTAAGCTATATTCAATATCTCCTGAGGCATATTCAGATGATAAGTTTGATAAGGCCAATTGCTACAAGCTAGAGAAGAGGTCTGAATATGAGCTAACTAGGCTCTACAAAGGACTAGTCCGGGACTTAGGGAATTCTTCCCCCTCCACTTATGCAGTTGAGAGATTGCTAGTCTTGGCAAGCCATCTGTATGAGACCAAGAAAGGGTCTTCTAACTTCTTCTTGACCGATTACCTGCCCAAGACAACATCAACAGCAAATCTTGATGCAGGCTTTTTGGCAAAGCTGAAAGAGACCCCTAAGGCCTCTGACCCCGATGTTTCCGATGTGACAGAAGTCAAGACAGCTAAGGCTACTCTAGACTCTGCTACAACTGATGCGGATACTAAAAAGGCCGCTTATGAGGCTATCGGAGATGAGGATTCAAAGAAGGCTGAGAAGGCTACAGCAAACACTGCCTGGATTGCTGCCCAAGAGGCCCAGAAGAAGGCTCAGAGTGCCTATGATAAGGCAGTTTCGAATGCGAAGAAGGCCTCAAGGAAAACTACCTCTGGAAAAAGCTTGTTCGGGGATGCAGGGGAGACTGTCACAGATGAGTCTAAGGTAGTGGAAGAGGTTGGCGAGGGGAAGAAGAAGTTTGGACCCTTCTTAGCTGCTTACTTGATGAGGCTACTGACCAAAATAGCATCCAATGTCACAGAGTCATGGGAGCATATGAAAGGAATGTATAAGAACTTTTATGGTTATGATGCTCCTTCAGACCTGAACTGTCCCGAGGCAGGATTCCTTGAGCAACTGAAGTCTGAGCTTAACAAGGATAGAAGGGCGGCTACCTCATGGGTCAAAATAGTAGCAGAGGCAGATAATAAGTTGGATCAATCTACAGCCGAGGCTGGGATTCTCCGCTATGTGGCTGTCCTCCCTCTTGCATATTCTGGGATGCATGCGATGAAGTTGTTCATGGACGTCAAGATGCTGACCAAACTCACCAGCAACTACTTGATTGGTGCCATGAGGAGCCCTCTGACCAAGGATGCATTAGATGCAATCATGGATATCTTGATCTCGTTTGAGTCCACTACAAAGACTAAGAAGTCGGAGAAGTTCCGGTTTGCTAGGATTGTGAGTACTCAATTCTTTCAATCCCTCCAGACCAAGAATTGCAAAGAGCTGGTCTATCTGATGGTTCAGATAATTGCTGAGTACAGGAAAGCAGAGGGGGTCAGGGATCCCATGAACATTGCTGGATTAGATGACATCTCTAGCAGGAACAAGAAGAAGTTGAATAAAGCATTACGGATAATACTGGCAGAAGCTCCAAAGGCATCCGCAGGTGAGTATTCGTCAGCCATGAAGAAGGCTTTCCTGGATGACGAAGAGGATGATACAGCCAAGACCAGGTCCATCTTCCAGACCAAGGCTTAAGGAACATCATGTTTGGATAGAATATGACTCCTCTAAATAACTATGCAGCCTACTGGCGAGCCTTTGTGTGTTATATAAGAAAAACGAGACCGTCAGGTCAATAATGAGTGTGCCAGAGGATACTCCCTTCAGATCGTACTCCAGCATCTTTGACGACTCAGACTTTGTCCAACCACAGCCCATGTCCTTCAAAGCGACCAAGGAGTCAGAGAGCCTTCCTGAGACAGAGAAAGAAGATATGTCGACTGAATACCTCTCTGAACCACTGAGGACCAAGTCAGGTAAGAAGAACAGACGGAGGAAGGGAAAGGACCTAAAATCTCTCTTCACTCAAGAAGCTGGACTCCCGGCTCCAGAAGCAGACAGTGTCCTCCCCGAATCGTCTCCGTATGAGAATGATAACGCACAGTTGGAGCTACCCAAGCCCATTCCAAAGACATCAGACGCTCCGGTCTTCCTAAGAGAGAAGGATCTTAGTAAAGAATTTGCTGCAGCCTGCAAGACTAATGGGATTCTCCCAAGGGATGAATGGAAGTCATCTGTAGCAGCCAAGTATCATGCAGAGGACGGGAAAATGACCAAACGAGACATCTCGTTAATCGTATTTGGAATGGAGCTTTACAAGAGATACAATGTGGAATCTGAGGTATCAACTTTGTTCACTTCATTGGTGACTGAGTTGCAGGGGATAAAGGTTGCTGCTAAGGAGTTGAATGATACCCGGGAGGTCCTCACTAAGATTCCAGGAGAAATTGTGTCTGCTGTCAAGGCAGGGGTAAAGGAGGGGACCGAGATGGGGATGGATTATATAGAGACTAGAACCAAAGTGGCCCCCAAGAGTGCTCCTAAAGTGGACATCTCCAAGCCGATGAGTAGTAAGATGATGGAGCAGCAGGATGAGAGTTCTGATGAGTCCTCAGATAATGAGAGTGAGGAGAGTGAGGAAGAATCCTTTGAGACAAAGGCTGCAATCTTCTTGGCTTTGATAAAGGTTCCAGAGGAAGAGAGAGACAACGCAATAGTCCTTATGGCCTTGAGGGCAGTGATATCTGACAGTGAGCTGAATCAGGCAATTAGGAATGACAGAATCTCCTCCTCAGTAGCAGATATGTACCATCAAAAGATATCTGACAAGGCTAGGGAATTGATGGGAAAGGGAAAGACCAACAAGAGGGCCAAGCAGCCTAAATCCTCTAAGTATGCATCGGATTACTATGATGATGCACTGTGAGTAATCAGTTACTACCTGTGTGGCAATGTCGGAACTGTACTTACCTATATATTGATAATCGTCTGTGATCTTGTGCCTTTAATTGCTGCAGTATTATAGCTTAAAATAATCTAGTTACCAATGCTCTATTTTCGTACTTAAGTGCCATGTTGCCTGCCTTTACCAAAGTAATCTAGTGTGCTCTATTTAAGAAAAACGAGACCTTCATCATGAAGATCATCTGCAGTACTGGGATCTTCAATGAAGAGAAAGGCTTCCCCCTCCCCAACCTCCTCAACAGTCCCTTGATGAAGCAGGAGATCATGACGGTCAAGTACTTGAGGTTTCAGTATATCCCTATTATGACCAGCAACCCCTCGAGTTCATTGACTATTGACATTAAAGATACTCGACTGGTCAATTGGGATAATAGGTCGATTTTCCAGGTCAAAATATTCGGGGATGTTCAAAGCTCATTTATTGTATCGGGTCTACAGCCTTATTCAGCTAGAGACCGTTGCCCTTATCTCCTCTCCCTTTCAGTAAACGCAGGGAAGGTTGTTCCAGGGACCAAATACGGCATCTTAAAATCTTATGCTGTGTACACATCTAAAGACTCAGGGATAATTTCGTCACAGATCTCTGTAAAACTTGAAAGGTCCCCACGTGACTACTTCTTGAAGAGGTCTAAGGAGCATGACAAAAAAGATCTTGACAGTGATGTCTCATTCAAGATGTGTCGCCATGTCAAGTTTGCTACTTGAAGCTTGGAGAGGATGGGCAGCTGAATGTTGTCGGGGTGATGTTTTCCTTATGAATATCTTCAATAATTTCCCTGGAGGGAGAAACAGTTGATCTCTGGGAGGACGCAAGGGCTCACTACTAGCATGTGCTTGCCAGGTTGAGGTTTTATGCCTATCATGGTTTAGCCTAAATAAGAAAAACGAGACCATCATGGCCGTTCCGTGGACTGAGGTTAAAGACTCCAAGTACTTGGCAACTAAGATGTCTGTTACCCTGATCATGGAGATGAATGAAGATACACCTCTAAAGTATCCTTCTTACAACGCATTCGAGAGCATCTTTAAAAGGCTAGCAGAACCAGAAAGCGCAGCACCCCAAGTGGCCGCATGGTTTACTTGGTTCCTAAGAGAGGCAAAGGACATCTACTATCTGGAAGTGAGTAACAAGGAAACTGCCCAATACGGACCAACTAAAGTCTATAAACTTCAGTGTCCAGCCTACCTCTTATCCCGAGTGACAGGAGGATCACAGCTTGATTATACCTCTCTTATAGGTAGCAAGGTGATGACTGAGAAAGATCGGGGGATCCCAGTCAGAACACTCTATATTACTGGAGGGGGTACATCATTCAGAGTCATCAACGAGGAGACAGCCAACCAGTTCATCATAAATGATAATGCTGTCCGCCTACCAGGAGAGTGCAAGGTGGATGGAGGAAGTATAATCTGGAGTTAGAATTTCAAGAAAACTAAACAATAATGAACAAGTGGAACAATCTCGTGTAGTGTATTAGGTGTCCTCTTTAACTAAAGTTAAATAAGAAAAACGAGACCCGCAAGGATCTTAAGATAACAAGATGATGAGGATTTCGGTCTTTCTCTTGATGCTCTGCTGGCTCCCTGTCAGCCTGACCTTCTTCGACAAATCACACATTCCTATAACCACATGTGATAAGAATCTAATGAGTCCTATCCCTTGGAGAACTTACTGCATAGAGGAGTGTGGAATCCGGAATGTAATAGGTGATAAGCTGGATCTGTTTATCTACAATAGGTCTGACAGTGGGAAAGTACAGCTGGCTGACTGCAGGAAGTACAAGATCAGACAGACCTTCACCAAGACTTGGACATTCTCGACATTCAAAGGGGCGATAGAGACAGAGGAGCTAATGCCTAATTATGCAGAGTGTGAATCCACCTGGAGAGATCTGTGCAACTCTGGACCGTGTAGTACCACAACTCCGGTGATCCCTGAGGAATACCATTGGGCTTCTGACACCACGAAGGAGGTCATCTATGTATCTATAGATGCATACCAGAAAACTGTTGCATTCCAGGATCCTAGTGGTGATATCCAGCTCCTAGTCCATGGGGTGATCATAGATGGGAGCCAGTCTGGTTATGTTCAACCCAGCAAAGATCTCATCACTATGTGGGATAAGGTTGAGTTACAAGATGAATGCCCTTGGTCAACGGGAAATTCTCTTTCGTGTTCTACGTCTGATGAGGGAATTTCATACTACTGTGCTGGGAAAGGGCTAGTACTGACCAACATCAGTACGGTGACTGATACCAGATGTGACAACAACCCGCACCTAATGACATCAGGGCACCATGTGATTTTTAGAGTGAAGAAGGCATCAGACCCGAATGCGACTCTCAGCAGGACAGCTCAAATAGTGCTGGACAGGGGGTCAGAAGAGGCCGAGATTGTAGATAGTGTTAATAAGGCGTTGCTGGATAGAGATTCCATCAGGTGTGCAAGCTCATGTCTCGCCTTTGATTACACCATCTCCAAGCCTCAGATGTTTGGCAACCAATTGGCGCTACCTTATAAGGGGTCTTTTCTCCCTTGCAACATACTGCCTAATTGTCGGGTTGTCTTCCCAGTCAAGTATTGCAGCTCTCCTCCGATGATTCTGGTAGAATGTACCGGCACTATGACATGGTGGAATATCACTGGAGATTACACGATCAGACCCACCTATTGCCACATGAACCAGTCGGCGACCAAGATTAAGACATCTATATCCTTTATGACAACAAATGGGAGAGTCTTAGTGAATGAGTCTGGCGCTTATCCTGTCTCCCGTGAAATAGGAAATACGTTCCAGGTCGGACATGTCATAGAGCCTAGCTCCATGATAGAGGTGACTGATCCACTTAATGTTAGGATAGATGACACCTTAGTCACACCAGAGTCCCATACTATATCTAATATCACTTCAGTCGGGGACTCACTTTTGGATACAATGGTTGAGACCGTGAAAGGTATCGGTCGCTTCATATCCCATGAGGTCAGGATAGTGGTTTTTGGTGTTCTGACTCTTTTTATATTATATCTGTCGTTTAAATATTTGTTCGCTAAGAAGAAGAGCAGAGTGCCGCACCCTAAAGTTGTCTATACAAAACCCACATCTGAAGGACCAGTGATCTATGACACCGAATATACTATAGAAAGTGACTAATAAAAAACAGAGACCAACATGGAGTTCAATTGGCCTTGGGGACAGAACAGTGAAACGGAAATCACCAAGAATCTCCGCTTTGAGGACATTAAGGTGATGGCCATAATAATACTAGTCTGGGTGAAGTGTCTTCTCATCTACCATTTCAAGAGGAAAATAAGGCGACTAAGATCTCTATTGATAAAAGGATCCTCACAATGGGTACTGCATGATGCCTAACTCATTAGGAAGATCTGAGATGAGTTTTTATCCTGCCTTATCCTTCTAATTAAGAAAAACGAGACCGCCATCATGGACCTTGATGACGGTGGTCTATGGAGACGTGCTAGGGGTCTGGGGGATTATCACCTGAGGTCTGCTCTGGTGACCCCCTCCTTAGAGCGTTTCCGCAGTCGAAAGGGAAGGCACCGTGAACAACTGTGCTTTGATAGGATGAAATCACTAGGCTGGATGTTGAGGTGGGTAGATCAAGGGAAACTGCTTGGATATTTAATGGTAGAAGCCAACAAATCTTTACCAAAATCCATTGCAAACCAGGAACTCTTAGTTGAGACTCTGAAGTTAGAATACGGATGTCTTAGACAGATAATCATGACGGATGGAGACCTCCATGATCAGGTGATCTCTTATCTAGACAGGAAGTCTATCTCTACCCATTACACCCATGGTAGGGAGGTCTTTCAGGAAGCCTTGATAGTTGTCATGGCACTTTCTTCAGGGAGAGAACCACCAGATCATGTCAATAACTTGGGTTATGAAATGCTGAATGAAGAACTTGAATTGCCCGTGGTCAGAACCTATGGCGTAATCTTCTATCTCTTTGGGGACTTGATCTACGTAAAGTATCCGGAGGAAGAGGGCATGATCACACTGGACATGTTCAGAAACCTGACAGATAAATTCTCTGAGAGGGAGAATATTATGATTGCAACTCAACTAGGAACTGAGATCCTTCAAGAGATATACCCTTCTGAGACAGTGCTCAAAACAGTCTTTTCTCTTTGGGACAAAGGTCTATTGAAAGAGGGAAATGACTTTTACACAGTGGTAAAGACATTCGAAGCAATCATAAATGGGATGTTGATCAAGAACAATGACGGAACATACTATGATCCTTCAGCATACCTCCGAGAAACTATCATGGGGCTGCCAGTACGACTGAGGGATTATGCACAGACGTTAGTGAGTTACTTAGACTCTCTCCCCTTTAATCCACACCACCTATCTCAGATTGGGGGGTTGTTTCGGTTGTGGGGACACCCCATAGTAGATCCTAACGCTGGGGTCAGAAAGGTAAGGCTTCTGGGAGCAGCTGATAAGATGAACCTGACACATATCCCCACCCTAGCAGAGCGGAAATTCAAGGAGATCTTCTACCTGTCGTATTATGAAAAGCATAGAGTCTATCCTAATCATAACTTGAATGGAGAAATTGAAGGAAGCTATTTGCTCTCTCAGCTTGCACAAATGGCTCTGGTTAATCCCAAGCATGCAAATTACTCACTAGTAGACTGGGATTCAGTCAACACACTAGAGACCTTTCCCTTTCCTAAATCATTCAACCTGTCTCTTATTATAGCGGATAAAGCTGTAAGTCCGAACAGAGAAGAATGGTTGGAATTGAGACGTAAGGGAGGAACTCAGATGGATCCGCACATTAGAAGAGGGCCTCTGAAGGCTATGAAAGACGGAGTAATCGATTGTGAGAAACTATTGAGGAAGATAGACCGTAACCCGTCCGGTTTAGCTAAGAAACATAGGATCATTGGACTGTACCCAAAGGAGAGAGAAGAAAATATGGTTCCCAGGATGTTTGCATTAATGTCCTTTGACATGAGAGCTTTCTCAGTGGTCTCTGAATCCATGATAGCAGATCACATCATTCCTCATATAGAAGGTGTCACAATGACGAAGAGCATGTTGGCCCTCCAGAAAGAGATGATAATTTCAACTAAAAGCCAGGCCTCCTCAACCCAGTCAGACAGTATAACCTTCTGTCTTAATATTGACTTTGAAAAGTGGAACCTTAACTTCCGAAGATGGATGACGGAGGGGGTATTCAGAGAAATGGGGCGATTGTTCGGACTTCCAGAGATATTCAATCGGACTTATGACATTTTCAAGAAATCCATCATTTATCTAGCAGATGGAAGCTTCGACCTCTTGCTGACAGATGAGTTGGAAATAGAACCAGGGACCAATCCAGATTGCGCATACACCGGTCATGTTGGGGGGTTTGAGGGATTGAGGCAGAAAGGATGGACAGTCTTCACAGCGGTCTTAATCTCTTCCATCTGCGATGAAATGGGGATCAAGACTCATCTAATGGGGCAAGGTGATAACCAGGTTTTGATGCTGACCATCTACTCAAGAGCTGCTAGAGAGACGGGGGATTTGAAATCAGCACCAGCAGTACTAGAGATAACAAATACATTGGAGACATTCAAGAGCAGACTAGTCTCACTCTTTGCTAATCTTGGATTGCCTATAAAACCACTAGAGACCTGGGTCTCAGAGGAATTGTTTGCCTATGGCAAGACTCCAATATACCGAGCAGTCCCCCTTGCAATGAGTCTCAAGCGAATCTCTAGAGTTTTTGCGTTTTCCAATGAGGATCTAATGACCTTGTCTAATGCGTTAGGTGCTATCTCTGCGAATGCACAAGCTGCATCCATGTGCGATGTCCATCCGATGGTGTCATATGCCATAGCAAAGTGGCAACACCTATGCTGTGCGATAATCTTCTCCAACTATCATCCGCTATGCGGGTGTGCTCCACATGTCTCAGGAGAGGAATGGGCAATAAAGCTACGACTTCCGTCAGGAAAGAAGATTCAAGAGACATCAGATGAGGAGATAGACGAAAGAGATCTGATGAAACTAATAGTGACCATACCTCGAAGCTTAGGAGGTTATAATACCCTGACTCTGTATGAGATGATCATGAGAGGATTTTCTGACCCAGTCTCTAGGGACATGTGCTGGCTATTCGCAATTGCCAGTGAATCAACAGGAAAACTTCGAGGTTATCTGATAAACTGGATTAAACCAATCGTGTCTCCGGAAGTAAATGCTCAGCATCTCATTCAGGATCCCACGGCACTCAATTTGCTAGTACCCCCTAATGCTACATCAGTTATTAAGAGGATGATAGACAAATCACTCGAAGCACTCCCAAAGAGATCTCAGTTCGCAGTATGGTTCTCTGAGATCCTAGAGATCTCAGGGGACAAGGAAATCTCAAAGTTGGCAGAGGCCTTGACCAGGACAGATTCCCTGAACCCCCGTTTCCTTCACGACATTCTGGGGGCCACTCTTTATGGATACTGCACGGCTATAACTAGTAAAGTTGACAAGACAGTGACCTTATCTCGGATGGCACTAGCGTCTAAGGATGTTGTTGGAGCTCTAATCAAGGGGGAGATGAGACTTTACTCCTATTTCGGATGGAGAACCTTGCAATCGAGAGGCTTACCTCTGACCACCAGATGTCCCAATAAGTGGGTGCGGATAATTAGGGATATCTCATGGCAAAAACAAATTAAGGCTGTTAGCGTTCCGTATCCTACTCATTTCTTATCAGAGGATATTTCAGAGACTGATAGGCCCGATTCCTGGATTGAGTGCTATATTGATGATGCCCCGACATCTGACAGAAGTTGCATGATCTATTCGACAGGAAAAGCTCTGCCGTACTTGGGTAGTGTCACTAGAGAAAAACTGACAACTCGAGGTGCAAAGGCGGCCTATGGAACTGAACCTCTTGTATTGAGGCCAATTAATCTAGTCAGAACTATAGGTTGGTTCATTGAAGAGGATAGCAATTTTGCTGAATTGATCAAGATGCTACTGGGAGCCGTCACAGATCTTCCCATAGAGGAGGTACTCTATATCCCAGAAATGGTGAGTGGCTCTATGGCACATAGATACCTAGATATGTCAACACAGCATGGATCTTTGTGGATGCCTCTTTATGGTCCTGCCACTTTTCTCCATATGAGCACTAATACTTTTGTGCAGTACCTGAAGGGGACAGAAAATGTCACTTTGCACTTTCAGTGTGTCATGGGCTTAATTCAGTATGCTATAGTCAACAAAGCTCTAGGGGAATGTCCCACAAAGAGAATGACGAGATTCTTCCGATCATGCCCTGACTGTATAGTTCCCATTGATGATACGTTGGAGGACTTGCCTGAGGTCCCTTCGCTAGATCTCATCCCTGAGAGGACCACAAACCCTTATCTCTACCTCAAGAAGGAGAAGATTGAACTGAATGTTAGGCATCGACTTGCAGTGATAAATGAGATCCGGGTAATAGCTAGGGATGAGATAGAAGAGACTCCCCTGTTGGCTAACAATACTCTGGAGGACGTGATGAGTTTGAGGGCAGCTCAGAGAATATTCTACACAGCCAAAGGAAAGGAGGCACAGTGGGACTTGCAGACTGCTGATCGAGAGGGGTACTTAAAACTGGATTTCGTGAGCGTAATCAGGAAGATTATAGGACACCTCTTTGTAATGGAATCAGAGACGTTAAAAGCCGGGTCAGACTACCCTACTTTCCAGTTACAGCAGAGAAGGATCATGCGGCGCGTTAGGCAGTCAGATACATCTAACTTTGTCCACTTAGGAGGGTTCTTCTGCTGGCAAGAGAGCATTGCGAGGATACAGAAACTGAAGTGGTCAGTGATGCCTGCCACATTCCCTATCACTGCAGAATCAGTGTCACTGGCAGCTAAGATGAGTCTAATAGGGGCAATGACTAGCGGGCTAACACCTAAGAGATGTGATGGTGTTCTGGCCGAAAACTTAATCCCGGATGTCACAAGGCAAGCAAAGAACATCATATGCCTGGACAAAGTGTTCCAAAGCAGATGTGATTACTGCTATACTGCAGCAATGACAAATAGGTGGTCAGACTCGATAAACTCCGACAGCATATTCAATATGAGATGTGAGAGAGGCCATACCATTCTGTCCCCGAGAATGTTGCATCAATTGAGGCGAACTATCTTGCCTGAAGGAGCCCTATACACTTTGGCTGTCAGGGTGGTTCACCAAAGCCCTCCTGAAGCATCACAACCTCTTCAGATCATCCCATGCAGGCGAGAGAGATATCAAATCTTGAGTGAAGCTGACATGCCTAGACAAACTGGCTACTCGGGGGTCTATAGAAGGCCTACCAATCAAATCTGCATGTATACAGAGGTTGAATTGGCAAAGAAGTACAGGTTGCCAACTAATTCATTATACAGGATCCTTGACTTACACGACTGCTTCTTCAGTCAGAAACTCATGGACACAAGGGGCAACATTTTAGTGGTCGGAGACGGGTATGGATACAGCTCCTTGCTAACCAAGTGCTTGAACCCAGACAGGAATGTAGTGAGCTGGACCTATATTGAACCATCTGAGGCACTTCCACATAGCCTGAGAATCTCTAAGCCTCCAATGCACTACAAAGCTGATGTGCAGATAGATTCTAGCCCTTCAATTGACAGAATCTCCGACATACATAACTCCTCATACCCGGATGAGTTCGCTAAAGTGGTAACCAAGAATGGGATTACAGCACTTATCAGTGACATTGAAACTGTGTATGTCTCTGGAGAGAAGAGTGTGGCGTCTTTGATCAACTTGGCTTGGTATAACCAGATCCAATTAGGAGCACTCAAGTTGGAATTGATGACGGATCCCTTGGAGAAAGTCGTAGAATATGCCCACAATGCTTACCAAAGATGGGAATTATTCACTCTACCAGGCGCCAATCTCGGAGGAGGGGTACTATACATAGGTTTCTACGGTAGAAGAGAGAAGTTGACTGGGTACATCATTCCCCATTCAGGAGTTGAGACCTTGATGGATAGGTTGGCAACTGAGGTAGATGAGGATAGAGGAAGATTGAGAGCAGAGGACCGTGATCGGTGGGAGCAGTTGGATACAATGGAAAGCAAAATACATCTGCAGCAATACCATAAGATGAGGCTCGATCTATGGTTCGGATCTGCTTATCTGAGTGGTTATCTCTCAGAGGATATGACTGAGTTCTTTTACTCGGTAAAAACATCCTACAGACCACCTGCAATCTCATGGGAGAAAGGGAACCAGGCCAGATACCTCTATGGCAGCAGGGAAGAGATCTTATTCATGAGCATGATCACAGTGGCACTATCATCCTATCTAGAGGACCAGGATGTCTTAAGAGAGTTCCTTTCTTCCTCAGGGTGGAAACTCCAGTGGAAGAAGGATAAGAAGAATCCGAGGCATTGGTCCCCGTATTTGGAGCGGAGTGACTCTCAACTAGTCTGGAAAAAGAACAAAGCCATGATTTTCAAACTGGTATCAGTTCTGAGAGGATCAAAGCCGAGAGAAGAATATAAGATGGGAAAAGCATCTGACTCCATAGTTTTCAAATACATTCCGAGGAAGGTAAGGGAAGGAGAAGTTCCCCTGTGCTTCCCGATCTCTAAGGTGGCCTCAATGTCGGTATAAGAAAAACGAGACCTTCATCATGGATGATTTAATGGTCTATAGCCTTTGGGCTCTGCTGCAATGGGGTTTGCCTGCGGATACAGCCAGATACATAATCTCACTGAGGATGGAAGAAGACTACTGGGAAGAGATGAATGAACATGAGCAGTGGGACGGGTACGAGTCTTGGGATGATACTGATTGGCAGTATCCGAGTCCTTGAATGAGTCAGTATGTGACAATTTAGAAAAAACGATTACCTTACTGTTGACTTTAAAGAACACTGTTATGTTGTGTTCGCAACACACAACTTCCTT

>Seq3 Rice stripe mosaic virus LJ isolate 1, complete genome

AAGGAAGTTGCGTTGCGAACGCAACATTAAAATGTTGATATAACCCACCTTATCATAACAAAAGAAAAACGAGACCGCCAAGTCTTAAAATGGCAACCGACAAGTCTTTTGAGGAGAAGCTGAGCCTGGTTCCTGAGAACACTAAGCTATATTCAATATCTCCTGAGGCATATTCAGATGATAAGTTTGATAAGGCCAATTGCTACAAGCTAGAGAAGAGATCTGAATATGAGCTAACTAGGCTCTATAAGGGACTAGTCCGGGACTTAGGGAATTCTTCCCCCTCCACTTATGCAGTCGAGAGATTGCTAGTCTTGGCAAGCCATCTGTATGAGACCAAGAAAGGGTCTTCTAACTTCTTCTTGACCGATTACCTGCCCAAGACAACATCAACAGCAAATCTTGATGCAGGCTTTTTGGCAAAGCTGAAAGAGACCCCTAAGGCCTCTGACCCCGATGTTTCCGATGTGACAGAAGTCAAGACAGCTAAGGCTACTCTAGACTCTGCTACAACTGATGCGGATACTAAAAAGGCCGCTTATGAGGCTATCGGAGATGAGGATTCAAAGAAGGCTGAGAAGGCTACAGCAAACACTGCCTGGATTGCTGCCCAAGAGGCCCAGAAGAAGGCTCAGAGTGCCTATGATAAGGCAGTTTCGAATGCGAAGAAGGCCTCAAGGAAAACTACCTCTGGAAAAAGCTTGTTCGGGGATGCAGGGGAGACAGTCACAGATGAGTCTAAGGTAGTGGAAGAGGTTGGAGAGGGGAAGAAGAAGTTTGGACCCTTCTTAGCTGCTTACTTGATGAGGCTACTGACCAAAATAGCATCCAATGTCACAGAGTCATGGGAGCATATGAAAGGAATGTATAAGAACTTTTATGGTTATGATGCTCCTTCAGACCTGAACTGTCCCGAGGCAGGATTCCTTGAGCAACTGAAGTCTGAGCTTAACAAGGATAGAAGGGCGGCTACCTCATGGGTCAAAATAGTAGCAGAGGCAGATAATAAGTTGGATCAATCTACAGCCGAGGCTGGGATTCTCCGCTATGTGGCTGTCCTCCCTCTTGCATATTCTGGGATGCATGCGATGAAGTTGTTCATGGACGTCAAGATGCTGACCAAACTCACCAGCAACTACTTGATTGGTGCCATGAGGAGCCCTCTGACCAAGGATGCATTAGGTGCAATCATGGATATCTTGATCTCGTTTGAGTCCACTACAAAGACTAAGAAGTCGGAGAAGTTCCGGTTTGCTAGGATTGTGAGTACTCAATTCTTTCAATCCCTCCAGACCAAGAATTGCAAAGAGCTGGTCTATCTGATGGTTCAGATAATTGCTGAGTACAGGAAAGCAGAGGGGGTCAGGGATCCCATGAACATTGCTGGATTAGATGACATCTCTAGCAGGAACAAGAAGAAGTTGAATAAAGCAGTACGGATAATACTGGCAGAAGCTCCAAAGGCATCCGCAGGTGAGTATTCGTCAGCCATGAAGAAGGCTTTCCTGGATGACGAAGAGGATGATACAGCCAAGACCAGGTCCATCTTCCAGACCAAGGCTTAAGGAACATCATGTTTGGATAGAATATGACTCCTCTAAATAACTATGCAGCCTACTGGCGAGTCTTTGTGTGTTATATAAGAAAAACGAGACCGTCAGGTCAATAATGAGTGTGCCAGAGGATACTCCCTTCAGATCGTACTCCAGCATCTTTGACGACTCAGACTTTGTCCAACCACAGCCCATGTCCTTCAAAGCGACCAAGGAGTCAGAGAGCCTTCCTGAGACAGAGAAAGAAGATATGTCGACTGAATACCTCTCTGAACCACTGAGGACCAAGTCAGGTAAGAAGAACAGACGGAGGAAGGGAAAGGACCTAAAATCTCTCTTCACTCAAGAAGCTGGACTCCCGGCTCCAGAAGCAGACAGTGTCCTCCCCGAATCGTCTCCATATGAGGATGATAACGCACAGTTGGAGCTACCAAAGCCCATTCTAAAGACATCAGACGCTCCGGTCTTCCTAAGAGAGAAGGATCTTAGTAAAGAATTTGCTGCAGCCTGCAAGACTAATGGGATTCTCCCAAGGGATGAATGGAAGTCATCTGTAGCAGCCAAGTATCATGCAGAGGAAGGGAAAATGACCAAACGAGACATCTCGTTAATCGTATTTGGAATGGAGCTTTACAAGAGATACAATGTGGAATCTGAGGTATCAACTTTGTTCACTTCATTGGTGACTGAGTTGCAGGGGATAAAGGTTGCTGCTAAGGAGTTGAATGATACCCGGGAGGTCCTCACTAAGATTCCAGGAGAAATTGTGTCTGCTGTCAAGGCAGGGGTAAAGGAGGGGACCGAGATGGGGATGGATTATATAGAGACTAGAACTAAAGTGGCCCCCAAGAGTGCTCCTAAAGTGGACATCTCCAAGCCGATGAGTAGTAAGATGATGGAGCAGCAGGATGAGAGTTCTGATGAGTCCTCAGATAATGAGAGTGAGGAGAGTGAGGAAGAATCCTTTGAGACAAAGGCTGCAATCTTCTTAGCTTTGATAAAGGTTCCAGAGGAAGAGAGAGACAACGCAATAGTCCTTATGGCCTTGAGGGCAGTGATATCTGACAGTGAGCTGAATCAGGCAATTAGGAATGACAGAATCTCCTCCTCAGTAGCAGATATGTACCATCAAAAGATATCTGACAAGGCAAGGGAATTGATGGGAAAGGGAAAGACCAACAAGAGGGCCAAGCAGCCTAAATCCTCTAAGTATGCATCGGATTACTATGATGATGCACTGTGAGTAATCAGTTACTACCTGTGTGGCAATGTCGGAACTGTACTTACCTATATATTGATAATCGTCTGTGATCTTGTGCCTTTAATTGCTGCAGTATTATAGCTTAAAATAATCTAGTTACCAATGCTCTATTTTCGTACTTAAGTGCCATGTTGCCTGCCTTTACCAAAGTGATCTAGTGTGCTCTATTTAAGAAAAACGAGACCTTCATCATGAAGATCATCTGCAGTACTGGGATCTTCAATGAAGAGAAAGGCTTCCCCCTCCCCAACCTCCTCAACAGTCCCTTGATGAAGCAGGAGATCATGACGGTCAAGTACTTGAGGTTTCAGTATATCCCTATTATGACCAGCAACCCCTCGAGTTCATTGACTATTGACATTAAAGATACTCGACTGGTCAATTGGGATAATAGGTCGATTTTCCAGGTCAAAATATTCGGGGATGTTCAAAGCTCATTTATTGTATCGGGTCTACAGCCTTATTCAGCTAGAGACCGTTGCCCTTATCTCCTCTCCCTTTCAGTAAACGCAGGGAAGGTTGTTCCAGGGACCAAATACGGCATCTTAAAATCTTATGCTGTGTACACATCTAAAGACTCAGGGATAATTTCGTCACAGATCTCTGTAAAACTTGAAAGGTCCCCACGTGACTACTTCTTGAAGAGGTCTAAGGAGCATGACAAAAAAGATCTTGACAGTGATGTCTCATTCAAGATGTGTCGCCATGTCAAGTTTGCTACTTGAAGCTTGGAGAGGATGGGCAGCTGAATGTTGTCGGGGTGATGTTTTCCTTATGAATATCTTCAATAATTTCCCTGGAGGGAGAAACAGTTGATCTCTGGGAGGACGCCAGGGCTCACTACTAGCATGTGCTTGCCAGGTTGAGGTTTTATGCCTATCATGGTTTAGCCTAAATAAGAAAAACGAGACCATCATGGCCGTTCCGTGGACTGAGGTTAAAGACTCCAAGTACTTGGCAACTAAGATGTCTGTTACCCTGATCATGGAGATGAATGAAGATACACCTCTAAAGTATCCTTCTTACAACGCATTCGAGAGCATCTTTAAAAGGCTAGCAGAACCAGAAAGCGCAGCACCCCAAGTGGCCGCATGGTTTACTTGGTTCCTAAGAGAGGCAAAGGACATCTACTATCTGGAAGTGAGTAACAAGGAAACTGCCCAATACGGACCAACTAAAGTCTATAAACTTCAGTGTCCAGCCTACCTCTTATCCCGAGTGACAGGAGGATCACAGCTTGATTATACCTCTCTTATAGGTAGCAAGGTGATGACTGAGAAAGATCGGGGGATCCCAGTCAGAACACTCTATATTACTGGAGGGGGTACATCATTCAGAGTCATCAACGAGGAGACAGCCAACCAGTTCATCATAAATGATAATGCTGTCCGCCTACCAGGAGAGTGCAAGGTGGATGGAGGAAGTATAATCTGGAGTTAGAATTTCAAGAAAACTAAACAATAATGAACAAGCGGAACAATCTCTTGTAGTGTATTAGGTGTCCTCTTTAACTAAAGTTAAATAAGAAAAACGAGACCCGCAAGGATCTTAAGATAACAAGATGATGAGGATTTCGGTCTTTCTCTTGATGCTCTGCTGGCTCCCTGTCAGCCTGACCTTCTTCGACAAATCGCACATTCCTATAACCACATGTGATAAGAATCTAATGAGTCCTATCCCTTGGAGAACTTACTGCATAGAGGAGTGTGGAATCCGGAATGTAATAGGTGATAAGCTGGATCTGTTTATCTACAATAGGTCTGACAGTGGGAAAGTACAGCTGGCTGACTGCAGGAAGTACAAGATCAGACAGACCTTCACCAAGACTTGGACATTCTCGACATTCAAAGGGGCGATAGAGACAGAGGAGCTAATGCCTAATTATGCAGAGTGTGAATCCACCTGGAGAGATCTGTGCAACTCTGGACCGTGTAGTACCACAACTCCGGTGATCCCTGAGGAATACCATTGGGCTTCTGACACCACGAAGGAGGTCATCTATGTATCTATAGATGCATACCAGAAAACTGTTGCATTCCAGGATCCTAGTGGTGATATCCAGCTCCTAGTCCATGGGGTGATCATAGATGGGAGCCAGTCTGGTTATGTTCAACCCAGCAAAGATCTCATCACTATGTGGGATAAGGTTGAGTTACAAGATGAATGCCCTTGGTCAACGGGAAATTCTCTTTCGTGTTCTACGTCTGATGAGGGAATTTCATACTACTGTGCTGGGAAAGGGCTAGTACTGACCAACATCAGTACGGTGACTGATACCAGATGTGACAACAACCCGCACCTAATGACATCAGGGCACCATGTGATTTTTAGAGTGAAGAAGGCATCAGACCCGAATGCGACTCTCAGCAGGACAGCTCAAATAGTGCTGGACAGGGGGTCAGAAGAGGCCGAGATTGTAGATAGTGTTAATAAGGCGTTGCTGGATAGAGATTCTATCAGGTGTGCAAGCTCATGTCTCGCCTTTGATTACACCATCTCCAAGCCTCAGATGTTTGGCAACCAATTGGCGCTACCTTATAAGGGGTCTTTTCTCCCTTGCAACATACTGCCTAATTGTCGGGTTGTCTTCCCAGTCAAGTATTGCAGCTCTCCTCCGATGATTCTGGTAGAATGTACCGGCACTATGACATGGTGGAATATCACTGGAGATTACACGATCAGACCCACCTATTGCCACATGAACCAGTCGGCGACCAAGATTAAGACATCTATATCCTTTATGACAACAAATGGGAGAGTCTTAGTGAATGAGTCTGGCGCTTATCCTGTCTCCCGTGAAATAGGAAATACGTTCCAGGTCGGACATGTCATAGAGCCTAGCTCCATGATAGAGGTGACTGATCCACTTAATGTTAGGATAGATGACACCTTAGTCACACCAGAGTCCCATACTATATCTAATATCACTTCAGTCGGGGACTCACTTTTGGATACAATGGTTGAGACCGTGAAAGGTATCGGTCGCTTCATATCCCATGAGGTCAGGATCGTGGTTTTTGGTGTTCTGACTCTTTTTATATTATATCTGTCGTTTAAATATTTGTTCGCTAAGAAGAAGAGCAGAGTGCCGCACCCTAAAGTTGTCTATACAAAACCCACATCTGAAGGACCAGTGATCTATGACACCGAATATACTATAGAAAGTGACTAATAAAAAACAGAGACCAACATGGAGTTCAATTGGCCTTGGGGACAGAACAGTGAAACGGAAATCACCAAGAATCTCCGCTTTGAGGACATTAAGGTGATGGCCATAATAATACTAGTCTGGGTGAAGTGTCTTCTCATCTACCATTTCAAGAGGAAAATAAGGCGACTAAGATCTCTATTGATAAAAGGATCCTCACAATGGGTACTGCATGATGCCTAACTCATTAGGAAGATCTGAGATGAGTTTTTATCCTGCCTTATCCTTCTAATTAAGAAAAACGAGACCGCCATCATGGACCTTGATGACGGTGGTCTATGGAGACGTGCTAGGGGTCTGGGGGATTATCACCTGAGGTCTGCTCTGGTGACCCCCTCCTTAGAGCGTTTCCGCAGTCGAAAGGGAAGGCACCGTGAACAACTGTGCTTTGATAGGATGAAATCACTAGGCTGGATGTTGAGGTGGGTAGATCAAGGGAAACTGCTTGGATATCTAATGGTAGAAGCCAACAAATCTTTACCAAAATCCATTGCAAACCAGGAACTCCTAGTTGAGACTCTGAAGTTAGAATACGGATGTCTTAGACAGATAATCATGACGGATGGAGACCTCCATGATCAGGTGATCTCTTATCTAGACAGGAAGTCTATCTCTACCCATTACACCCATGGTAGGGAGGTCTTTCAGGAAGCCTTGATAGTTGTCATGGCACTTTCTTCAGGGAGAGAACCACCAGATCATGTCAATAACTTGGGTTATGAAATGCTGAATGAAGAACTTGAAGTGCCCGTGGTCAGAACCTATGGCGTAATCTTCTATCTCTTTGGGGACTTGATCTACGTAAAGTATCCGGAAGAAGAGGGCATGATCACACTGGACATGTTCAGAAACCTGACAGATAAATTCTCTGAGAGGGAGAATATTATGATTGCAACTCAACTAGGAACTGAGATCCTTCAAGAGATATACCCTTCTGAGACAGTGCTCAAAACAGTATTTTCTCTTTGGGACAAAGGTCTATTGAACGAGGGAAATGACTTTTACACAGTGGTAAAGACATTCGAAGCAATCATAAATGGGATGTTGATCAAGAACAATGATGGAACATACTATGATCCTTCAGCATACCTCCGAGAAACTATCATGGGGCTGCCAGTACGACTGAGGGATTATGCACAGACGTTAGTGAGTTACTTAGACTCTCTCCCCTTTAATCCACACCACCTATCTCAGATTGGGGGGTTGTTTCGGTTGTGGGGACACCCCATAGTAGATCCTAACGCTGGGGTCAGAAAGGTAAGGCTTCTGGGAACAGCTGATAAGATGAACTTGACACATATCCCTACCCTAGCAGAGCGGAAATTCAAGGAGATCTTCTACCTGTCGTATTATGAAAAGCATAGAGTCTATCCTAATCATAACTTGAATGGAGAAATTGAAGGAAGCTATTTGCTCTCTCAGCTTGCACAAATGGCTCTGGTTAATCCCAAGCATGCAAATTACTCACTAGTAGACTGGGATTCAGTCAACACACTAGAGACCTTTCCCTTTCCTAAATCATTCAACCTGTCTCTTATTATAGCGGATAAAGCTGTAAGTCCGAACAGAGAAGAATGGTTGGAATTGAGACGTAAGGGAGGAACTCAGATGGATCCGCACATTAGAAGAGGGCCTCTGAAGGCTATGAAAGACGGAGTAATCGATTGTGAGAAACTATTGAGGAAGATAGACCGTAACCCGTCCGGTTTAGCTAAGAAACATAGGATCATTGGACTGTACCCAAAGGAGAGAGAAGAAAATATGGTTCCCAGGATGTTTGCATTAATGTCCTTTGACATGAGAGCTTTCTCAGTGGTCTCTGAATCCATGATAGCAGATCACATCATTCCTCATATAGAAGGTGTCACAATGACGAAGAGCATGTTGGCCCTCCAGAAAGAGATGATAATTTCAACTAAAAGCCAGGCCTCCTCGACCCAGTCAGACAGTATAACCTTCTGTCTTAATATTGACTTTGAAAAGTGGAACCTTAACTTCCGAAGATGGATGACGGAGGGGGTATTCAGAGAAATGGGGCGATTGTTCGGACTTCCAGAGATATTCAATCGGACTTATGACATTTTCAAGAAATCCATCATTTATCTAGCAGATGGAAGCTTCGACCTCTTGCTGACAGATGAGTTGGAAATAGAACCAGGGACCAATCCAGATTGCGCATACACCGGTCATGTTGGGGGGTTTGAGGGATTGAGGCAGAAAGGATGGACAGTCTTCACAGCGGTCTTAATCTCTTCCATCTGCGATGAAATGGGGATCAAGACTCATCTAATGGGGCAAGGTGATAACCAGGTTTTGATGCTGACCATCTACTCAAGAGCTGCTAGAGAGACGGGGGATTTGAAATCAGCACCAGCAGTACTAGAGATCACAAATACATTGGAGACATTCAAGAGCAGACTAGTCTCACTCTTTGCTAATCTTGGATTGCCTATAAAACCACTAGAGACCTGGGTCTCAGAGGAATTGTTTGCCTATGGCAAGACTCCAATATACCGAGCAGTCCCCCTTGCAATGAGTCTCAAGCGAATCTCTAGAGTTTTTGCGTTTTCCAATGAAGATCTAATGACCTTGTCTAATGCGTTAGGTGCTATCTCTGCGAATGCACAAGCTGCATCCATGTGCGATGTCCATCCGATGGTGTCATATGCCATAGCAAAGTGGCAACACCTATGCTGTGCGATAATCTTCTCCAACTATCATCCGCTATGCGGGTGTGCTCCACATGTCTCAGGAGAGGAATGGGCAATAAAGCTACGACTTCCGTCAGGAAAGAAGATTCAAGAGACATCTGATGAGGAGATAGACGAAAGAGATCTGATGAAACTAATAGTGACCATACCTCGAAGCTTAGGAGGTTATAATACCCTGACTCTGTATGAGATGATCATGAGAGGATTTTCTGACCCAGTCTCTAGGGACATGTGCTGGCTATTCGCAATTGCCAGTGAATCAACAGGAAAACTTCGAGGTTATCTGATAAACTGGATTAAACCAATCGTGTCTCCGGAAGTAAATGCTCAGCATCTCATTCAGGATCCCACAGCACTCAATTTGCTAGTACCCCCTAATGCTACATCAGTTATTAAGAGGATGATAGACAAATCACTCGAAGCACTCCCAAAGAGATCTCAGTTCGCAGTATGGTTCTCTGAGATCCTAGAGATCTCAGGGGACAAGGAAATCTCAAAGTTAGCAGAGGCCTTGACCAGGACAGATTCCCTGAACCCCCGTTTCCTTCACGACATTCTGGGGGCCACTCTTTATGGATACTGCACGGCTATAACTAGTAAAGTTGACAAGACAGTGACCTTATCTCGGATGGCACTAGCGTCTAAGGATGTTGTTGGAGCTCTAATCAAGGGGGAGATGAGACTTTACTCCTATTTCGGATGGAGAACCTTGCAATCGAGAGGCTTACCTCTGACCACCAGATGTCCCAATAAGTGGGTGCGGATAATTAGGGATATCTCATGGCAAAAACAAATTAAGGCTGTTAGCGTTCCGTATCCTACTCATTTCTTATCAGAGGATATTTCAGAGACTGATAGGCCCGATTCCTGGATTGAGTGCTATATTGATGATGCCCCGACATCTGACAGAAGTTGCATGATCTATTCGACAGGAAAAGCTCTGCCGTACTTGGGTAGTGTCACTAGAGAAAAACTGACAACTCGAGGTGCAAAGGCGGCCTATGGAACTGAACCTCTTGTATTGAGGCCAATTAATCTAGTCAGAACTATCGGTTGGTTCATTGAAGAGGATAGCAATTTTGCTGAATTGATCAAGATGCTACTGGGAGCCGTCACCGATCTTCCCATAGAGGAGGTACTCTATATCCCAGAAATGGTGAGTGGCTCTATGGCACATAGATACCTAGATATGTCAACACAGCATGGATCTTTGTGGATGCCTCTTTATGGTCCTGCCACTTTTCTCCATATGAGCACTAATACTTTTGTGCAGTACCTGAAGGGGACAGAAAATGTCACTTTGCACTTTCAGTGTGTCATGGGCTTAATTCAGTATGCTATAGTCAACAAAGCTCTAGGGGAATGTCCCACAAAGAGAATGACGAGATTCTTCCGATCATGCCCTGACTGTATAGTTCCCATTGATGATACGTTGGAGGACTTGCCTGAGGTCCCTTCGCTAGATCTCATCCCTGAGAGGACCACAAACCCTTATCTCTACCTCAAGAAGGAGAAGATTGAACTGAATGTTAGGCATCGACTTGCAGTGATAAATGAGATCCGGGTAATAGCTAGGGATGAGATAGAAGAGACTCCCCTGTTGGCTAACAATACTCTGGAGGACGTGATGAGTTTGAGGGCAGCTCAGAGAATATTCTACACAGCCAAAGGAAAGGAGGCACAGTGGGACTTGCAGACTGCTGATCGAGAGGGGTACTTAAAACTGGATTTCGTGAGCGTAATCAGGAAGATTATAGGACACCTCTTTGTAATGGAATCAGAGACGTTAAAAGCCGGGTCAGACTACCCTACTTTCCAGTTACAGCAGAGAAGGATCATGCGGCGCGTTAGGCAGTCAGATACGTCTAACTTTGTCCACTTAGGAGGGTTCTTCTGCTGGCAAGAGAGCATTGAGAGGATACAGAAACTGAAGTGGTCAGTGATGCCTGCCACATTCCCTATCACTGCAGAATCAGTGTCACTGGCAGCTAAGATGAGTCTAATAGGGGCAATGACTAGCGGGCTAACACCTAAGAGATGTGATGGTGTTCTGGCCGAAAACTTAATCCCGGATGTCACAAGGCAAGCAAAGAACATCATATGCCTGGACAAAGTGTTCCAAAGCAGATGTGATTACTGCTATACTGCAGCAATGACAAATAGGTGGTCTGACTCGATAAACTCCGACAGCATATTCAATATGAGATGTGAGAGAGGCCATACCATTCTGTCCCCGAGAATGTTGCATCAATTGAGGCGAACTATCTTGCCTGAAGGAGCCCTATACACTTTGGCTGTCAGAGTGGTTCACCAAAGCCCTCCTGAAGCATCACAACCTCTTCAGATCATCCCATGCAGGCGAGAGAGATATCAAATCTTGAGTGAAGCTGACATGCCCAGACAAACTGGCTACTCGGGGGTCTATAGAAGGCCTACCAATCAAATCTGCATGTATACAGAGGTTGAATTGGCAAAGAAGTACAGGTTGCCAACTAATTCATTATACAGGATCCTTGACTTACACGACTGCTTCTTCAGTCAGAAACTCATGGACACGAGGGGCAACATTTTAGTGGTCGGAGACGGGTATGGATACAGCTCCTTGCTAACCAAGTGCTTGAACCCAGACAGGAATGTAGTGAGCTGGACCTATATTGAACCATCTGAGGCACTTCCACATAGCCTGAGAATCTCTAAGCCTCCTATGCACTACAAAGCTGATGTGAAGATAGATTCTAGCCCTTCAATTGACAGAATCTCCGACATACATAACTCCTCATACCCGGATGAGTTCGCTAAAGTGGTAACCAAGAATGGGATTACAGCACTGATTAGTGACATTGAAACTGTGTATGTCTCTGGAGAGAAGAGTGTGGCGTCTCTGATCAACTTGGCTTGGAATAACCAGATCCAATTAGGAGCACTCAAGTTGGAATTGATGACAGATCCCTTGGAGAAAGTCGTAGAATATGCCCACAATGCTTACCAAAGATGGGAATTATTCACTCTACCAGGCGCCAATCTCGGAGGAGGGGTACTATACATAGGTTTCTACGGTAGAAGAGAGAAGTTGACTGGGTACATCATTCCCCATTCAGGAGTTGAGACCTTGATGGATAGGTTGGCAACTGAGGTAGATGAGGATAGAGGAAGATTGAGAGCAGAGGACCGTGATCGGTGGGAGCAGTTGGATACAATGGAAAGCAAAATACATCTGCAGCAATACCATAAGATGAGGCTCGATCTATGGTTCGGATCTGCTTATCTGAGTGGTTATCTCTCAGAGGATATGACTGAGTTCTTTTACTCGGTAAAAACATCCTACAGACCACCTGCAATCTCATGGGAGAAAGGGAACCAGGCCAGATACCTCTATGGCAGCAGGGAAGAGATCTTATTCATGAGCATGATCACAGTGGCACTATCATCCTATCTAGAGGACCAGGATGTCTTAAGAGAGTTCCTTTCTTCCTCAGGGTGGAAACTCCAGTGGAAGAAGGATAAGAAGAATCCGAGGCATTGGTCCCCGTATTTGGAGCGGAGTGACTCTCAACTAGTCTGGAAAAAGAACAAAGCCATGATTTTCAAACTGGTATCAGTTCTGAGAGGATCAAAGCCGAGAGAAGAATATAAGATGGGAAAAGCATCTGACTCCATAGTTTTCAGATACATTCCGAGGAAGGTAAGGGAAGGAGAAGTTCCCCTGTGCTTCCCGATCTCTAAGGTGGCCTCAATGTCGGTATAAGAAAAACGAGACCTTCATCATGGATGATTTAATGGTCTATAGCCTTTGGGCTCTGCTGCAATGGGGTTTGCCTGCGGATACAGCCAGATACATAATCTCACTGAGGATGGAAGAAGACTACTGGGAAGAGATGAATGAACATGAGCAGTGGGACGGGTACGAGTCTTGGGATGATACTGATTGGCAGTATCCGAGTCCTTGAATAAGTCAGTATGTGACAATTTAGAAAAAACGATTACCTTACTGTTGACTTTAAAGAACACTGTTATGTTGTGTTCGCAACACACAACTTCCTT

>Seq4 Rice stripe mosaic virus LJ isolate 2, complete genome

AAGGAAGTTGCGTTGCGAACGCAACATTAAAATGTTGATATAACCCACCTTATCATAACAAAAGAAAAACGAGACCGCCAAGTCTTAAAATGGCAACCGACAAGTCTTTTGAGGAGAAGCTGAGCCTGGTTCCTGAGAACACTAAGCTATATTCAATATCTCCTGAGGCATATTCAGATGATAAGTTTGATAAGGCCAATTGCTACAAGCTAGAGAAGAGATCTGAATATGAGCTAACTAGGCTCTATAAGGGACTAGTCCGGGACTTAGGGAATTCTTCCCCCTCCACTTATGCAGTCGAGAGATTGCTAGTCTTGGCAAGCCATCTGTATGAGACCAAGAAAGGGTCTTCTAACTTCTTCTTGACCGATTACCTGCCCAAGACAACATCAACAGCAAATCTTGATGCAGGCTTTTTGGCAAAGCTGAAAGAGACCCCTAAGGCCTCTGACCCCGATGTTTCCGATGTGACAGAAGTCAAGACAGCTAAGGCTACTCTAGACTCTGCTACAACTGATGCGGATACTAAAAAGGCCGCTTATGAGGCTATCGGAGATGAGGATTCAAAGAAGGCTGAGAAGGCTACAGCAAACACTGCCTGGATTGCTGCCCAAGAGGCCCAGAAGAAGGCTCAGAGTGCCTATGATAAGGCAGTTTCGAATGCGAAGAAGGCCTCAAGGAAAACTACCTCTGGAAAAAGCTTGTTCGGGGATGCAGGGGAGACAGTCACAGATGAGTCTAAGGTAGTGGAAGAGGTTGGAGAGGGGAAGAAGAAGTTTGGACCCTTCTTAGCTGCTTACTTGATGAGGCTACTGACCAAAATAGCATCCAATGTCACAGAGTCATGGGAGCATATGAAAGGAATGTATAAGAACTTTTATGGTTATGATGCTCCTTCAGACCTGAACTGTCCCGAGGCAGGATTCCTTGAGCAACTGAAGTCTGAGCTTAACAAGGATAGAAGGGCGGCTACCTCATGGGTCAAAATAGTAGCAGAGGCAGATAATAAGTTGGATCAATCTACAGCCGAGGCTGGGATTCTCCGCTATGTGGCTGTCCTCCCTCTTGCATATTCTGGGATGCATGCGATGAAGTTGTTCATGGACGTCAAGATGCTGACCAAACTCACCAGCAACTACTTGATTGGTGCCATGAGGAGCCCTCTGACCAAGGATGCATTAGGTGCAATCATGGATATCTTGATCTCGTTTGAGTCCACTACAAAGACTAAGAAGTCGGAGAAGTTCCGGTTTGCTAGGATTGTGAGTACTCAATTCTTTCAATCCCTCCAGACCAAGAATTGCAAAGAGCTGGTCTATCTGATGGTTCAGATAATTGCTGAGTACAGGAAAGCAGAGGGGGTCAGGGATCCCATGAACATTGCTGGATTAGATGACATCTCTAGCAGGAACAAGAAGAAGTTGAATAAAGCAGTACGGATAATACTGGCAGAAGCTCCAAAGGCATCCGCAGGTGAGTATTCGTCAGCCATGAAGAAGGCTTTCCTGGATGACGAAGAGGATGATACAGCCAAGACCAGGTCCATCTTCCAGACCAAGGCTTAAGGAACATCATGTTTGGATAGAATATGACTCCTCTAAATAACTATGCAGCCTACTGGCGAGTCTTTGTGTGTTATATAAGAAAAACGAGACCGTCAGGTCAATAATGAGTGTGCCAGAGGATACTCCCTTCAGATCATACTCCAGCATCTTTGACGACTCAGACTTTGTCCAACCACAGCCCATGTCCTTCAAAGCGACCAAGGAGTCAGAGAGCCTTCCTGAGACAGAGAAAGAAGATATGTCGACTGAATACCTCTCTGAGCCACTGAGGACCAAGTCAGGTAAGAAGAACAGACGGAGGAAGGGAAAGGACCTAAAATCTCTCTTCACTCAAGAAGCTGGACTCCCGGCTCCAGAAGCAGACAGTGTCCTCCCTGAATCGTCTCCGTATGAGAATGATAACGCACAGTTGGAGCTACCCAAGCCCATTCTAAAGACATCGGACGCTCCGGTCTTCCTGAGAGAGAAGGATCTTAGTAAAGAATTTGCTGCAGCCTGCAAGACTAATGGGATTCTCCCAAGGGATGAATGGAAGTCATCTGTAGCAGCCAAGTATCATGCAGAGGAAGGGAAAATGACCAAACGAGACATCTCGTTAATCGTATTTGGAATGGAGCTTTACAAGAGATACAATGTGGAATCTGAGGTATCAACTTTGTTCACTTCATTGGTGACTGAGTTGCAGGGGATAAAGGTTGCTGCTAAGGAGTTGAATGATACCCGGGAGGTCCTCACTAAGATTCCAGGAGAGATTGTGTCTGCTGTCAAGGCAGGGGTAAAGGAGGGGACCGAGATGGGGATGGATTATATAGAGACTAGAACTAAAGTGGCCCCCAAGAGTGCTCCTAAAGTGGACATCTCCAAGCCGATGAGTAGTAAGATGATGGAGCAGCAGGATGAGAGTTCTGATGAGTCCTCAGATAATGAGAGTGAGGAGAGTGAGGAAGAATCCTTTGAGACAAAGGCTGCAATCTTCTTAGCTTTGATAAAGGTTCCAGAGGAAGAGAGAGACAACGCAATAGTCCTTATGGCCTTGAGGGCAGTGATATCTGACAGTGAGCTGAATCAGGCAATTAGGAATGACAGAATCTCCTCCTCAGTAGCAGATATGTACCATCAAAAGATATCTGACAAGGCTAGGGAATTGATGGGAAAGGGAAAGACCAACAAGAGGGCCAAGCAGCCTAAATCCTCTAAGTATGCATCGGATTACTATGATGATGCACTGTGAGTAATCAGTTACTACCTGTGTGGCAATGTCGGAACTGTACTTACCTATATATTGATAATCGTCTGTGATCTTGTGCCTTTAATTGCTGCAGTATTATAGCTTAAAATAATCTAGTTACCAATGCTCTATTTTCGTACTTAAGTGCCATGTTGCCTGCCTTTACCAAAGTAATCTAGTGTGCTCTATTTAAGAAAAACGAGACCTTCATCATGAAGATCATCTGCAGTACTGGGATCTTCAATGAAGAGAAAGGCTTCCCCCTCCCCAACCTCCTCAACAGTCCCTTGATGAAGCAGGAGATCATGACGGTCAAGTACTTGAGGTTTCAGTATATCCCTATTATGACCAGCAACCCCTCGAGTTCATTGACTATTGACATTAAAGATACTCGACTGGTCAATTGGGATAATAGGTCGATTTTCCAGGTCAAAATATTCGGGGATGTTCAAAGCTCATTTATTGTATCGGGTCTACAGCCTTATTCAGCTAGAGACCGTTGCCCTTATCTCCTCTCCCTTTCAGTAAACGCAGGGAAGGTTGTTCCAGGGACCAAATACGGCATCTTAAAATCTTATGCTGTGTACACATCTAAAGACTCAGGGATAATTTCGTCACAGATCTCTGTAAAACTTGAAAGGTCCCCACGTGACTACTTCTTGAAGAGGTCTAAGGAGCACGACAAAAAAGATCTTGACAGTGATGTCTCATTCAAGATGTGTCGCCATGTCAAGTTTGCTACTTGAAGCTTGGAGAGGATGGGCAGCTGAATGTTGTCGGGGTGATGTTTTCCTTATGAATATCTTCAATAATTTCCATGGAGGGAGAAACAGTTGATCTCTGGGAGGACGCAGGGGCTCACTACTAGCATGTGCTTGCCAGGTTGGGGTTTTATGCCTATCATGGTTTAGCCTAAATAAGAAAAACGAGACCATCATGGCCGTTCCGTGGACTGAGGTTAAAGACTCCAAGTACTTGGCAACTAAGATGTCTGTTACCCTGATCATGGAGATGAATGAAGATACACCTCTAAAGTATCCTTCTTACAACGCATTCGAGAGCATCTTTAAAAGGCTAGCAGAACCAGAAAGCGCAGCACCCCAAGTGGCCGCATGGTTTACTTGGTTCCTAAGAGAGGCAAAGGACATCTACTATCTGGAAGTGAGTAACAAGGAAACTGCCCAATACGGACCAACTAAAGTCTATAAACTTCAGTGTCCAGCCTACCTCTTATCCCGAGTGACAGGAGGATCACAGCTTGATTATACCTCTCTTATAGGTAGCAAGGTGATGACTGAGAAAGATCGGGGGATCCCAGTCAGAACACTCTATATTACTGGAGGGGGTACATCATTCAGAGTCATCAACGAGGAGACAGCCAACCAGTTCATCATAAATGATAATGCTGTCCGCCTACCAGGAGAGTGCAAGGTGGATGGAGGAAGTATAATCTGGAGTTAGAATTTCAAGAAAACTAAACAATAATGAACAAGTGGAACAATCTCGTGTAGTGTATTAGGTGTCCTCTTTAACTAAAGTTAAATAAGAAAAACGAGACCCGCAAGGATCTTAAGATAACAAGATGATGAGGATTTCGGTCTTTCTCTTGATGCTCTGCTGGCTCCCTGTCAGCCTGACCTTCTTCGACAAATCACACATTCCTATAACCACATGTGATAAGAATCTAATGAGTCCTATCCCTTGGAGAACTTACTGCATAGAGGAGTGTGGAATCCGGAATGTAATAGGTGATAAGCTGGATCTGTTTATCTACAATAGGTCTGACAGTGGGAAAGTACAGCTGGCTGACTGCAGGAAGTACAAGATCAGACAGACCTTCACCAAGACTTGGACATTCTCGACATTCAAAGGGGCGATAGAGACAGAGGAGCTAATGCCTAATTATGCAGAGTGTGAATCCACCTGGAGAGATCTGTGCAACTCTGGACCGTGTAGTACCACAACTCCGGTGATCCCTGAGGAATACCATTGGGCTTCTGACACCACGAAGGAGGTCATCTATGTATCTATAGATGCATACCAGAAAACTGTTGCATTCCAGGATCCTAGTGGTGATATCCAGCTCCTAGTCCATGGGGTGATCATAGATGGGAGCCAGTCTGGTTATGTTCAACCCAGCAAAGATCTCATCACTATGTGGGATAAGGTTGAGTTACAAGATGAATGCCCTTGGTCAACGGGAAATTCTCTTTCGTGTTCTACGTCTGATGAGGGAATTTCATACTACTGTGCTGGGAAAGGGCTAGTACTGACCAACATCAGTACGGTGACTGATACCAGATGTGACAACAACCCGCACCTAATGACATCAGGGCACCATGTGATTTTTAGAGTGAAGAAGGCATCAGACCCGAATGCGACTCTCAGCAGGACAGCTCAAATAGTGCTGGACAGGGGGTCAGAAGAGGCCGAGATTGTAGATAGTGTTAATAAGGCGTTGCTGGATAGAGATTCCATCAGGTGTGCAAGCTCATGTCTCGCCTTTGATTACACCATCTCCAAGCCTCAGATGTTTGGCAACCAATTGGCGCTACCTTATAAGGGGTCTTTTCTCCCTTGCAACATACTGCCTAATTGTCGGGTTGTCTTCCCAGTCAAGTATTGCAGCTCTCCTCCGATGATTCTGGTAGAATGTACCGGCACTATGACATGGTGGAATATCACTGGAGATTACACGATCAGACCCACCTATTGCCACATGAACCAGTCGGCGACCAAGATTAAGACATCTATATCCTTTATGACAACAAATGGGAGAGTCTTAGTGAATGAGTCTGGCGCTTATCCTGTCTCCCGTGAAATAGGAAATACGTTCCAGGTCGGACATGTCATAGAGCCTAGCTCCATGATAGAGGTGACTGATCCACTTAATGTTAGGATAGATGACACCTTAGTCACACCAGAGTCCCACACTATATCTAATATCACTTCAGTCGGGGACTCACTTTTGGATACAATGGTTGAGACCGTGAAAGGTATCGGTCGCTTCATATCCCATGAGGTCAGGATAGTGGTTTTTGGTGTTCTGACTCTTTTTATATTATATCTGTCGTTTAAATATTTGTTCGCTAAGAAGAAGAGCAGAGTGCCGCACCCTAAAGTTGTCTATACAAAACCCACATCTGAAGGACCAGTGATCTATGACACCGAATATACTATAGAAAGTGACTAATAAAAAACAGAGACCAACATGGAGTTCAATTGGCCTTGGGGACAGAACAGTGAAACGGAAATCACCAAGAATCTCCGCTTTGAGGACATTAAGGTGATGGCCATAATAATACTAGTCTGGGTGAAGTGTCTTCTCATCTACCATTTCAAGAGGAAAATAAGGCGACTAAGATCTCTATTGATAAAAGGATCCTCACAATGGGTACTGCATGATGCCTAACTCATTAGGAAGATCTGAGATGAGTTTTTATCCTGCCTTATCCTTCTAATTAAGAAAAACGAGACCGCCATCATGGACCTTGATGACGGTGGTCTATGGAGACGTGCTAGGGGTCTGGGGGATTATCACCTGAGGTCTGCTCTGGTGACCCCCTCCTTAGAGCGTTTCCGCAGTCGAAAGGGAAGGCACCGTGAACAACTGTGCTTTGATAGGATGAAATCACTAGGCTGGATGTTGAGGTGGGTAGATCAAGGGAAACTGCTTGGATATTTAATGGTAGAAGCCAACAAATCTTTACCAAAATCCATTGCAAACCAGGAACTCCTAGTTGAGACTCTGAAGTTAGAATACGGATGTCTTAGACAGATAATCATGACGGATGGAGACCTCCATGATCAGGTGATCTCTTATCTAGACAGGAAGTCTATCTCTACCCATTACACCCATGGTAGGGAGGTCTTTCAGGAAGCCTTGATAGTTGTCATGGCACTTTCTTCAGGGAGAGAACCACCAGATCATGTCAATAACTTGGGTTATGAAATGCTGAATGAAGAACTTGAAGTGCCCGTGGTCAGAACCTATGGCGTAATCTTCTATCTCTTTGGGGACTTGATCTACGTAAAGTATCCGGAGGAAGAGGGCATGATCACACTGGACATGTTCAGAAACCTGACAGATAAATTCTCTGAGAGGGAGAATATTATGATTGCAACTCAACTAGGAACTGAGATCCTTCAAGAGATATACCCTTCTGAGACAGTGCTCAAAACAGTCTTTTCTCTTTGGGACAAAGGTCTATTGAAAGAGGGAAATGACTTTTACACAGTGGTAAAGACATTCGAAGCAATCATAAATGGGATGTTGATCAAGAACAATGACGGAACATACTATGATCCTTCAGCATACCTCCGAGAAACTATCATGGGGCTGCCAGTACGACTGAGGGATTATGCACAGACGTTAGTGAGTTACTTAGACTCTCTCCCCTTTAATCCACACCACCTATCTCAGATTGGGGGGTTGTTTCGGTTGTGGGGACACCCCATAGTAGATCCTAACGCTGGGGTCAGAAAGGTAAGGCTTCTGGGAACAGCTGATAAGATGAACCTGACACATATCCCCACCCTAGCAGAGCGGAAATTCAAGGAGATCTTCTACCTGTCGTATTATGAAAAGCATAGAGTCTATCCTAATCATAACTTGAATGGAGAAATTGAAGGAAGCTATTTGCTCTCTCAGCTTGCACAAATGGCTCTGGTTAATCCCAAGCATGCAAATTACTCACTAGTAGACTGGGATTCAGTCAACACACTAGAGACCTTTCCCTTTCCTAAATCATTCAACCTGTCTCTTATTATAGCGGATAAAGCTGTAAGTCCGAACAGAGAAGAATGGTTGGAATTGAGACGTAAGGGAGGAACTCAGATGGATCCGCACATTAGAAGAGGGCCTCTGAAGGCTATGAAAGACGGAGTAATCGATTGTGAGAAACTATTGAGGAAGATAGACCGTAACCCGTCCGGTTTAGCTAAGAAACATAGGATCATTGGACTGTACCCTAAGGAGAGAGAAGAAAATATGGTTCCTAGGATGTTTGCATTAATGTCCTTTGACATGAGAGCTTTCTCAGTGGTCTCTGAATCCATGATAGCAGATCACATCATTCCTCATATAGAAGGTGTCACAATGACGAAGAGCATGTTGGCCCTCCAGAAAGAGATGATAATTTCAACTAAAAGCCAGGCCTCCTCAACCCAGTCAGACAGTATAACCTTCTGTCTTAATATTGACTTTGAAAAGTGGAACCTTAACTTCCGAAGATGGATGACGGAGGGGGTATTCAGAGAAATGGGGCGATTGTTCGGACTTCCAGAGATATTCAATCGGACTTATGACATCTTCAAGAAATCCATCATTTATCTAGCAGATGGAAGCTTCGACCTCTTGCTGACAGATGAGTTGGAAATAGAACCAGGGACCAATCCAGATTGCGCATACACCGGTCATGTTGGGGGGTTTGAGGGATTGAGGCAGAAAGGATGGACAGTCTTCACAGCGGTCTTAATCTCTTCCATCTGCGATGAAATGGGGATCAAGACTCATCTAATGGGGCAAGGTGATAACCAGGTTTTGATGCTGACCATCTACTCAAGAGCTGCTAGAGAGACGGGGGATTTGAAATCAGCACCAGCAGTACTAGAGATCACAAATACATTGGAGACATTCAAGAGCAGACTAGTCTCACTCTTTGCTAATCTTGGATTGCCTATAAAACCACTAGAGACCTGGGTCTCAGAGGAATTGTTTGCCTATGGCAAGACTCCAATATACCGAGCAGTCCCCCTTGCAATGAGTCTCAAGCGAATCTCTAGAGTTTTTGCGTTTTCCAATGAGGACCTAATGACCTTGTCTAATGCGTTAGGTGCTATCTCTGCGAATGCACAAGCTGCATCCATGTGCGATGTCCATCCGATGGTGTCATATGCCATAGCAAAGTGGCAACACCTATGCTGTGCGATAATCTTCTCCAACTATCATCCGCTATGCGGGTGTGCTCCACATGTCTCAGGAGAGGAATGGGCAATAAAGCTACGACTTCCGTCAGGAAAGAAGATTCAAGAGACATCTGATGAGGAGATAGACGAAAGAGATCTGATGAAACTAATAGTGACCATACCTCGAAGCTTAGGAGGTTATAATACCCTGACTCTGTATGAGATGATCATGAGAGGATTTTCTGACCCAGTCTCTAGGGACATGTGCTGGCTATTCGCAATTGCCAGTGAATCAACAGGAAAACTTCGAGGTTATCTGATAAACTGGATTAAACCAATCGTGTCTCCGGAAGTAAATGCTCAGCATCTCATTCAGGATCCCACGGCACTCAATTTGCTAGTACCCCCTAATGCTACATCAGTTATTAAGAGGATGATAGACAAATCACTCGAAGCACTCCCAAAGAGATCTCAGTTCGCAGTATGGTTCTCTGAGATCCTAGAGATCTCAGGGGACAAAGAAATCTCAAAGTTAGCAGAGGCCTTGACCAGGACAGATTCCTTGAACCCCCGTTTCCTTCACGACATTCTGGGGGCCACTCTTTATGGATACTGCACGGCTATAACTAGTAAAGTTGACAAGACAGTGACCTTATCTCGGATGGCACTAGCGTCTAAGGATGTTGTTGGAGCTCTAATCAAGGGGGAGATGAGACTTTACTCCTATTTCGGATGGAGAACCTTGCAATCGAGAGGCTTACCTCTGACCACCAGATGTCCCAATAAGTGGGTGCGGATAATTAGGGATATCTCATGGCAAAAACAAATTAAGGCTGTTAGCGTTCCGTATCCTACTCATTTCTTATCAGAGGATATTTCAGAGACTGATAGGCCCGATTCCTGGATTGAGTGCTATATTGATGATGCCCCGACATCTGACAGAAGTTGCATGATCTATTCGACAGGAAAAGCTCTGCCGTACTTGGGTAGTGTCACTAGAGAAAAACTGACAACTCGAGGTGCAAAGGCGGCCTATGGAACTGAACCTCTTGTATTGAGGCCAATTAATCTAGTCAGAACTATAGGTTGGTTCATTGAAGAGGATAGCAATTTTGCTGAATTGATCAAGATGCTACTGGGAGCCGTCACAGATCTTCCCATAGAGGAGGTACTCTATATCCCAGAAATGGTGAGTGGCTCTATGGCACATAGATACCTGGATATGTCAACACAGCATGGATCTTTGTGGATGCCTCTTTATGGTCCTGCCACTTTTCTCCATATGAGCACTAATACTTTTGTGCAGTACCTGAAGGGGACAGAAAATGTCACTTTGCACTTTCAGTGTGTCATGGGCTTAATTCAGTATGCTATAGTCAACAAAGCTCTAGGGGAATGTCCCACAAAGAGAATGACGAGATTCTTCCGATCATGCCCTGACTGTATAGTTCCCATTGATGATACGTTGGAGGACTTGCCTGAGGTCCCTTCGCTAGATCTCATCCCTGAGAGGACCACAAACCCTTATCTCTACCTCAAGAAGGAGAAGATTGAACTGAATGTTAGGCATCGACTTGCAGTGATAAATGAGATCCTGGTAATAGCTAGGGATGAGATAGAAGAGACTCCCCTGTTGGCTAACAATACTCTGGAGGACGTGATGAGTTTGAGGGCAGCTCAGAGAATATTCTACACAGCCAAAGGAAAGGAGGCACAGTGGGACTTGCAGACTGCTGATCGAGAGGGGTACTTAAAACTGGATTTCGTGAGCGTAATCAGGAAGATTATAGGACACCTCTTTGTAATGGAATCAGAGACGTTAAAAGCCGGGTCAGACTACCCTACTTTTCAGTTACAGCAGAGAAGGATCATGCGGCGCGTTAGGCAGTCAGATACATCTAACTTTGTCCACTTGGGAGGGTTCTTCTGCTGGCAAGAGAGCATTGCGAGGATACAGAAACTGAAGTGGTCAGTGATGCCTGCCACATTCCCTATCACTGCAGAATCAGTGTCACTGGCAGCTAAGATGAGTCTAATAGGGGCAATGACTAGCGGGCTAACACCTAAGAGATGTGATGGTGTTCTGGCCGAAAACTTAATCCCGGATGTCACAAGGCAAGCAAAGAACATCATATGCCTGGACAAAGTGTTCCAAAGCAGATGTGATTACTGCTATACTGCAGCAATGACAAATAGGTGGTCAGACTCGATAAACTCCGACAGCATATTCAATATGAGATGTGAGAGAGGCCATACCATTCTGTCCCCGAGAATGTTGCATCAATTGAGGCGAACTATCTTGCCTGAAGGAGCCCTATACACTTTGGCTGTCAGAGTGGTTCACCAAAGCCCTCCTGAAGCATCACAACCTCTTCAGATCATCCCATGCAGGCGAGAGAGATATCAAATCTTGAGTGAAGCTGACATGCCTAGACAAACTGGCTACTCGGGGGTCTATAGAAGGCCTACCAATCAAATCTGCATGTATACAGAGGTTGAATTGGCAAAGAAGTACAGGTTGCCAACTAATTCATTATACAGGATCCTTGACTTACACGACTGCTTCTTCAGTCAGAAACTCATGGACACAAGGGGCAACATTTTAGTGGTCGGAGACGGGTATGGATACAGCTCCTTGCTAACCAAGTGCTTGAACCCAGACAGGAATGTAGTGAGCTGGACCTATATTGAACCATCTGAGGCACTTCCACATAGCCTGAGAATCTCTAAGCCTCCAATGCACTACAAAGCTGATGTGCAGATAGATTCTAGCCCTTCAATTGACAGAATCTCCGACATACATAACTCCTCATACCCGGATGAGTTCGCTAAAGTGGTAACCAAGAATGGGATTACAGCACTGATCAGTGACATTGAAACTGTGTATGTCTCTGGGGAGAAGAGTGTGGCGTCCTTGATCAACTTGGCTTGGAATAACCAGATCCAATTAGGAGCACTCAAGTTGGAATTGATGACGGATCCCTTGGAGAAAGTCGTAGAATATGCCCACAATGCTTACCAAAGATGGGAATTATTCACTCTACCAGGCGCCAATCTCGGAGGAGGGGTACTATACATCGGTTTCTACGGTAGAAGAGAGAAGTTGACTGGGTACATCATTCCCCATTCAGGAGTTGAGACCTTGATGGATAGGTTGGCAACTGAGGTAGATGAGGATAGAGGAAGATTGAGAGCAGAGGACCGTGATCGGTGGGAGCAGTTGGATACAATGGAAAGCAAAATACATCTGCAGCAATACCATAAGATGAGGCTCGATCTATGGTTCGGATCTGCTTATCTGAGTGGTTATCTCTCAGAGGATATGACTGAGTTCTTTTACTCGGTAAAAACATCTTACAGACCACCTGCAATCTCATGGGAGAAAGGGAACCAGGCCAGATACCTCTATGGCAGCAGGGAAGAGATCTTATTCATGAGCATGATCACAGTGGCACTATCATCCTATCTAGAGGACCAGGATGTCTTAAGAGAGTTCCTTTCTTCCTCAGGGTGGAAACTCCAGTGGAAGAAGGATAAGAAGAATCCGAGGCATTGGTCCCCGTATTTGGAGCGGAGTGACTCTCAACTAGTCTGGAAAAAGAACAAAGCCATGATTTTCAAACTGGTATCAGTTCTGAGAGCATCAAAGCCGAGAGAAGAATATAAGATGGGAAAAGCATCTGACTCCATAGTTTTCAGATACATTCCGAGGAAGGTAAGGGAAGGAGAAGTTCCCCTGTGCTTCCCGATCTCTAAAGTGGCCTCAATGTCGGTATAAGAAAAACGAGACCTTCATCATGGATGATTTAATGGTCTATAGCCTTTGGGCTCTGCTGCAATGGGGTTTGCCTGCGGATACAGCCAGATACATAATCTCACTGAGGATGGAAGAAGACTACTGGGAAGAGATGAATGAACATGAGCAGTGGGACGGGTACGAGTCTTGGGATGATACTGATTGGCAGTATCCGAGTCCTTGAATAAGTCAGTATGTGACAATTTAGAAAAAACGATTACCTTACTGTTGACTTTAAAGAACACTGTTATGGTGTGTTCGCAACACACAACTTCCTT

>Seq5 Rice stripe mosaic virus SG isolate 1, complete genome

AAGGAAGTTGCGTTGCGAACGCAACATTAAAATGTTGATATAACCCACCTTATCATAACAAAAGAAAAACGAGACCGCCAAGTCTTAAAATGGCAACCGACAAGTCTTTTGAGGAGAAGCTGAGCCTGGTTCCTGAGAACACTAAGCTATATTCAATATCTCCTGAGGCATATTCAGATGATAAGTTTGATAAGGCCAATTGCTACAAGCTAGAGAAGAGGTCTGAATATGAGCTAACTAGGCTCTACAAAGGACTAGTCCGGGACTTAGGGAATTCTTCCCCCTCCACTTATGCAGTCGAGAGATTGCTAGTCTTGGCAAGCCATCTGTATGAGACCAAGAAAGGGTCTTCTAACTTCTTCTTGACCGATTACCTGCCCAAGACAACATCAACAGCAAATCTTGATGCAGGCTTTTTGGCAAAGCTGAAAGAGACCCCTAAGGCCTCTGACCCCGATGTTTCCGATGTGACAGAAGTCAAGACAGCTAAGGCTACTCTAGACTCTGCTACAACTGATGCGGATACTAAAAAGGCCGCTTATGAGGCTATCGGAGATGAGGATTCAAAGAAGGCTGAGAAGGCTACAGCAAACACTGCCTGGATTGCTGCCCAAGAGGCCCAGAAGAAGGCTCAGAGTGCCTATGATAAGGCAGTTTCGAATGCGAAGAAGGCCTCAAGGAAAACTACCTCTGGAAGAAGTTTGTTCGGGGATGCAGGGGAGACAGTCACAGATGAGTCTAAGGTAGTGGAAGAGGTTGGAGAGGGGAAGAAGAAGTTTGGACCCTTCTTAGCTGCTTACTTGATGAGGCTACTGACCAAAATAGCATCCAATGTCACAGAGTCATGGGAGCATATGAAAGGAATGTATAAGAACTTTTATGGTTATGATGCTCCTTCAGACCTGAACTGTCCCGAGGCAGGATTCCTTGAGCAACTGAAGTCTGAGCTTAACAAGGATAGAAGGACGGCTACCTCATGGGTCAAAATAGTAGCAGAGGCAGATAATAAGTTGGATCAATCTACAGCCGAGGCCGGGATTCTCCGCTATGTGGCTGTCCTCCCTCTTGCATATTCTGGGATGCATGCGATGAAGTTGTTCATGGACGTCAAGATGCTGACCAAACTCACCAGCAACTACTTGATTGGTGCCATGAGGAGCCCTCTGACCAAGGATGCATTAGATGCAATCATGGATATCTTGATCTCGTTTGAGTCCACTACAAAGACTAAGAAGTCGGAGAAGTTCCGGTTTGCTAGGATTGTGAGTACTCAATTCTTTCAATCCCTCCAGACCAAGAATTGCAAAGAGCTGGTCTATCTGATGGTTCAGATAATTGCTGAGTACAGGAAAGCAGAGGGGGTCAGGGATCCCATGAACATTGCTGGATTAGATGACATCTCTAGCAGGAACAAGAAGAAGTTGAATAAAGCAGTACGGATAATACTGGCAGAAGCTCCAAAGGCGTCCGCAGGGGAGTATTCGTCAGCCATGAAGAAGGCTTTCCTGGATGACGAAGAGGATGATACAGCCAAGACCAGGTCCATCTTCCAGACCAAGGCTTAAGGAACATCATGTTTGGATAGAATATGACTCCTCTAAATAACTATGCAGCCTACTGGCGAGTCTTTGTGTGTTATATAAGAAAAACGAGACCGTCAGGTCAATAATGAGTGTGCCAGAGGATACTCCCTTCAGATCGTACTCCAGCATCTTTGACGACTCAGACTTTGTCCAACCACAGCCCATGTCCTTCAAAGCGACCAAGGAGTCAGAGAGCCTTCCTGAGACTGAGAAGGAAGATATGTCGACTGAATACCTCTCTGAACCACTGAGGACCAAGTCAGGTAAGAAGAACAGGCGGAGGAAGGGAAAGGACCTAAAATCTCTCTTCACTCAAGAAGCTGGACTCCCGGCTCCAGAAGCAGACAGTGTCCTCCCCGAATCGTCTCCGTATGAGAATGATAACACACAGTTGGAGCTACCCAAGCCCATTCTAAAGACATCAGACGCTCCGGTCTTCCTAAGAGAGAAGGATCTTAGTAAAGAATTTGCTGCAGCCTGCAAGACTAATGGGATTCTCCCAAGGGATGAATGGAAATCATCTGTAGCAGCCAAGTATCATGCAGAGGAAGGGAAAATGACCAAACGAGACATCTCGTTAATCGTATTTGGAATGGAGCTTTACAAGAGATACAATGTGGAATCTGAGGTATCAACTTTGTTCACTTCATTGGTGACTGAGTTGCAGGGGATAAAGGTTGCTGCTAAGGAGTTGAATGATACCCGGGAGGTCCTCACTAAGATTCCAGGAGAAATTGTGTCTGCTGTCAAGGCAGGGGTAAAGGAGGGGACCGAGATGGGGATGGATTATATAGAGACTAGAACTAAAGTGGCCCCCAAGGGTGCTCCTAAAGTGGACATCTCCAAGCCGATGAGTAGTAAGATGATGGAGCAGCAGGATGAGAGTTCTGATGAGTCCTCAGATAATGAGAGTGAGGAGAGTGAGGAAGAATCCTTTGAGACAAAGGCTGCAATCTTCTTAGCTTTGATAAAGGTTCCAGAGGAAGAGAGAGACAACGCAATAGTCCTTATGGCCTTGAGGGCAGTGATATCTGACAGTGAGCTGAATCAGGCAATTAGGAATGACAGAATCTCTTCCTCAGTAGCAGATATGTACCATCAAAAGATATCTGACAAGGCTAGGGAATTGATGGGAAAGGGAAAGACCAACAAGAGGGCCAAGCAGCCTAAATCCTCTAAGTATGCATCGGATTACTATGATGATGCACTGTGAGTAATCAGTTACTACCTGTGTGGCAATGTCGGAACTGTACTTACCTATATATTGATAATCGTCTGTGATCTTGTGCCTTTAATTGCTGCAGTATTATAGCTTAAAATAATCTAGTTACCAATGCTCTATTTTCGTACTTAAGTGCCATGTTGCCTGCCTTTACCAAAGTGATCTAGTGTGCTCTATTTAAGAAAAACGAGACCTTCATCATGAAGATCATCTGCAGTACTGGGATCTTCAATGAAGAGAAAGGCTTCCCCCTCCCCAACCTCCTCAACAGTCCCTTGATGAAGCAGGAGATCATGACGGTCAAGTACTTGAGGTTTCAGTATATCCCTATTATGACCAGCAACCCCTCGAGTTCATTGACTATTGACATTAAAGATACTCGACTGGTCAATTGGGATAATAGGTCGATTTTCCAGGTCAAAATATTCGGGGATGTTCAAAGCTCATTTATTGTATCGGGTCTACAGCCTTATTCAGCTAGGGACCGTTGCCCTTATCTCCTCTCCCTTTCAGTAAACGCAGGGAAGGTTGTTCCAGGGACCAAATACGGCATCTTAAAATCTTATGCTGTGTACACATCTAAAGACTCAGGGATAATTTCGTCACAGATCTCTGTAAAACTTGAAAGGTCCCCACGTGACTACTTCTTGAAGAGGTCTAAGGAGCATGACAAAAAAGATCTTGACAGTGACGTCTCATTCAAGATGTGTCGCCATGTCAAGTTTGCTACTTGAAGCTTGGAGAGGATGGGCAGCTGAATGTTGTCGGGGTGATGTTTTCCTTATGAATATCTTCAATAATTTCCCTGGAGGGAGAAACAGTTGATCTCTGGGAGGACGCAAGGGCTCACTACTAGCATGTGCTTGCCAGGTTGAGGTTTTATGCCTATCATGGTTTAGCCTAAATAAGAAAAACGAGACCATCATGGCCGTTCCGTGGACTGAGGTTAAAGACTCCAAGTACTTGGCAACTAAGATGTCTGTTACCCTGATCATGGAGATGAATGAAGATACACCTCTAAAGTATCCTTCTTACAACGCATTCGAGAGCATCTTTAAAAGGCTAGCAGAACCAGAAAGCGCAGCACCCCAAGTGGCCGCATGGTTTACTTGGTTCCTAAGAGAGGCAAAGGACATCTACTATCTGGAAGTGAGTAACAAGGAAACTGCCCAATACGGACCAACTAAAGTCTATAAACTTCAGTGTCCAGCCTACCTCTTATCCCGAGTGACAGGAGGATCACAGCTTGATTATACCTCTCTTATAGGTAGCAAGGTGATGACTGAGAAAGATCGGGGGATCCCAGTCAGAACACTCTATATTACTGGAGGGGGTACATCATTCAGAGTCATCAACGAGGAGACAGCCAACCAGTTCATCATAAATGATAATGCTGTCCGCCTACCAGGAGAGTGCAAGGTGGATGGAGGAAGTATAATCTGGAGTTAGAATTTCAAGAAAACTAAACAATAATGAACAAGTGGAACAATCTCGTGTAGTGTATTAGGTGTCCTCTTTAACTAAAGTTAAATAAGAAAAACGAGACCCGCAAGGATCTTAAGATAACAAGATGATGAGGATTTCGGTCTTTCTCTTGATGCTCTGCTGGCTCCCTGTCAGCCTGACCTTCTTCGACAAATCACACATTCCTATAACCACATGTGATAAGAATCTAATGAGTCCTATCCCTTGGAGAACTTACTGCATAGAGGAGTGTGGAATCCGGAATGTAATAGGTGATAAGCTGGATCTGTTTATCTACAATAGGTCTGACAGTGGGAAAGTACAGCTGGCTGACTGCAGGAAGTACAAGATCAGACAGACCTTCACCAAGACTTGGACATTCTCGACATTCAAAGGGGCGATAGAGACAGAGGAGCTAATGCCTAATTATGCAGAGTGTGAATCCACCTGGAGAGATCTGTGCAACTCTGGACCGTGTAGTACCACAACTCCGGTGATCCCTGAGGAATACCATTGGGCTTCTGACACCACGAAGGAGGTCATCTATGTATCTATAGATGCATACCAGAAAACTGTTGCATTCCAGGATCCTAGTGGTGATATCCAGCTCCTAGTCCATGGGGTGATCATAGATGGGAGCCAGTCTGGTTATGTTCAACCCAGCAAAGATCTCATCACTATGTGGGATAAGGTTGAGTTACAAGATGAATGCCCTTGGTCAACGGGAAATTCTCTTTCGTGTTCTACGTCTGATGAGGGAATTTCATACTACTGTGCTGGGAAAGGGCTAGTACTGACCAACATCAGTACGGTGACTGATACCAGATGTGACAACAACCCGCATCTAATGACATCAGGGCACCATGTGATTTTTAGAGTGAAGAAGGCATCAGACCCGAATGCGACTCTCAGCAGGACAGCTCAAATAGTGCTGGACAGGGGGTCAGAAGAGGCCGAGATTGTAGATAGTGTTAATAAGGCGTTGCTGGATAGAGATTCTATCAGGTGTGCAAGCTCATGTCTCGCCTTTGATTACACCATCTCCAAGCCTCAGATGTTTGGCAACCAATTGGCGCTACCTTATAAGGGGTCTTTTCTCCCTTGCAACATACTGCCTAATTGTCGGGTTGTCTTCCCAGTCAAGTATTGCAGCTCTCCTCCGATGATTCTGGTAGAATGTACCGGCACTATGACATGGTGGAATATCACTGGAGATTACACGATCAGACCCACCTATTGCCACATGAACCAGTCGGCGACCAAGATTAAGACATCTATATCCTTTATGACAACAAATGGGAGAGTCTTAGTGAATGAGTCTGGCGCTTATCCTGTCTCCCGTGAAATAGGAAATACGTTCCAGGTCGGACATGTCATAGAGCCTAGCTCCATGATAGAGGTGACTGATCCACTTAATGTTAGGATAGATGACACCTTAGTCACACCAGAGTCCCATACTATATCTAATATCACTTCAGTCGGGGACTCACTTTTGGATACAATGGTTGAGACTGTGAAAGGTATCGGTCGCTTCATATCCCATGAGGTCAGGATAGTGGTTTTTGGTGTTCTGACTCTTTTTATGTTATATCTGTCGTTTAAATATTTGTTCGCTAAGAAGAAGAGCAGAGTGCCGCACCCTAAAGTTGTCTATACAAAACCCACATCTGAAGGACCAGTGATCTATGACACCGAATATACTATAGAAAGTGACTAATAAAAAACAGAGACCAACATGGAGTTCAATTGGCCTTGGGGACAGAACAGTGAAACGGAAATCACCAAGAATCTCCGCTTTGAGGACATTAAGGTGATGGCCATAATAATACTAGTCTGGGTGAAGTGTCTTCTCATCTACCATTTCAAGAGGAAAATAAGGCGACTAAGATCTCTATTGATAAAAGGATCCTCACAATGGGTACTGCATGATGCCTAACTCATTAGGAAGATCTGAGATGAGTTTTTATCCTGCCTTATCCTTCTAATTAAGAAAAACGAGACCGCCATCATGGACCTTGATGACGGTGGTCTATGGAGACGTGCTAGGGGTCTGGGGGATTATCACCTGAGGTCTGCTCTGGTGACCCCCTCCTTAGAGCGTTTCCGCAGTCGAAAGGGAAGGCACCGTGAACAACTGTGCTTTGATAGGATGAAATCACTAGGCTGGATGTTGAGGTGGGTAGATCAAGGGAAACTGCTTGGATATCTAATGGTAGAAGCCAACAAATCTTTACCAAAATCCATTGCAAACCAGGAACTCCTAGTTGATACTCTGAAGTTAGAATATGGATGTCTTAGACAGATAATCATGACGGATGGAGACCTCCATGATCAGGTGATCTCTTATCTAGACAGGAAGTCTATCTCTACCCATTACACCCATGGTAGGGAGGTCTTTCAGGAAGCCTTGATAGTTGTCATGGCACTTTCTTCAGGGAGAGAACCACCAGATCATGTCAATAACTTGGGTTATGAAATGCTGAATGAAGAACTTGAAGTGCCCGTGGTCAGAACCTATGGCGTAATCTTCTATCTCTTTGGGGACTTGATCTATGTAAAGTATCCGGAGGAAGAGGGCATGATCACACTGGACATGTTCAGAAACCTGACAGATAAATTCTCTGAGAGGGAGAATATTATGATTGCAACTCAAATAGGAACTGAGATCCTTCAAGAGATATACCCTTCTGAGACAGTGCTCAAAGCAGTCTTTTCTCTTTGGGACAAAGGTCTATTGAAAGAGGGAAATGACTTTTACACAGTGGTAAAGACATTCGAAGCAATCATAAATGGGATGTTGATCAAGAACAATGACGGAACATACTATGATCCTTCAGCATACCTCCGAGAAACTGTCATGGGGCTGCCAGTACGACTGAGGGATTATGCACAGACGTTAGTGAGTTACTTAGACTCTCTCCCCTTTAATCCACACCACCTATCTCAGATTGGGGGGTTGTTTCGGTTGTGGGGACACCCCATAGTAGATCCTAACGCTGGGGTCAGAAAGGTAAGGCTTCTGGGAACAGCTGATAAGATGAACCTGACACATATTCCCACCCTAGCAGAGCGGAAATTCAAGGAGATCTTCTACCTGTCGTATTATGAAAAGCATAGAGTCTATCCTAATCATAACTTGAATGGAGAAATTGAAGGAAGCTATTTGCTCTCTCAGCTTGCACAAATGGCTCTGGTTAATCCCAAGCATGCAAATTACTCACTAGTAGACTGGGATTCAGTCAACACACTAGAGACCTTTCCCTTTCCTAAATCATTCAACCTGTCTCTTATTATAGCGGATAAAGCTGTAAGTCCGAACAGAGAAGAATGGTTGGAATTGAGACGTAAGGGAGGAACTCAGATGGATCCGCACATTAGAAGAGGGCCTCTGAAGGCTATGAAAGACGGAGTAATCGATTGTGAGAAACTATTGAGGAAGATAGACCGTAACCCGTCCGGTTTAGCTAAGAAACATAGGATCATTGGACTGTACCCAAAGGAGAGAGAAGAAAATATGGTTCCCAGGATGTTTGCATTAATGTCCTTTGACATGAGAGCTTTCTCAGTGGTCTCTGAATCCATGATAGCAGATCACATCATTCCTCATATAGAAGGTGTCACAATGACGAAGAGCATGTTGGCCCTCCAGAAAGAGATGATAATTTCAACTAAAAGCCAGGCCTCCTCAACCCAGTCAGACAGTATAACCTTCTGTCTTAATATTGACTTTGAAAAGTGGAACCTTAACTTCCGAAGATGGATGACGGAGGGGGTATTCAGAGAAATGGGGCGATTGTTCGGACTTCCAGAGATATTCAATCGGACTTATGACATTTTCAAGAAATCCATCATTTATCTAGCAGATGGAAGCTTCGACCTCTTGCTGACAGATGAGTTGGAAATAGAACCAGGGACCAATCCAGATTGCGCATACACCGGTCATGTTGGGGGGTTTGAGGGATTGAGGCAGAAAGGATGGACAGTCTTCACAGCGGTCTTAATCTCTTCCATCTGCGATGAAATGGGGATCAAGACTCATCTAATGGGGCAAGGTGATAACCAGGTTTTGATGCTGACCATCTACTCAAGAGCTGCTAGAGAGACGGGGGATTTGAAATCAGCACCAGCAGTACTAGAGATCACAAATACATTGGAGACATTCAAGAGCAGACTAGTCTCACTCTTTGCTAATCTTGGATTGCCTATAAAACCACTAGAGACCTGGGTCTCAGAAGAATTGTTTGCCTATGGCAAGACTCCAATATACCGAGCAGTCCCCCTTGCAATGAGTCTCAAGCGAATCTCTAGAGTTTTTGCGTTTTCCAATGAGGATCTAATGACCTTGTCTAATGCGTTAGGTGCTATCTCTGCGAATGCACAAGCTGCATCCATGTGCGATGTCCATCCGATGGTGTCATATGCCATAGCAAAGTGGCAACACCTATGCTGTGCGATAATCTTCTCCAACTATCATCCGCTATGCGGGTGTGCTCCACATGTCTCAGGAGAGGAATGGGCAATAAAGCTACGACTTCCGTCAGGAAAGAAGATTCAAGAGGCATCTGATGGGGAGATAGACGAAAGAGATCTGATGAAACTAATAGTGACCATACCTCGAAGCTTAGGAGGTTATAATACCCTGACTCTGTATGAGATGATCATGAGAGGATTTTCTGACCCAGTCTCTAGGGACATGTGCTGGCTATTCGCAATTGCCAGTGAATCAACAGGAAAACTTCGAGGTTATCTGATAAACTGGATTAAACCAATCGTGTCTCCGGAAGTAAATGCTCAGCATCTCATTCAGGATCCCACGGCACTCAATTTGCTAGTACCCCCTAATGCTACATCAGTTATTAAGAGGATGATAGACAAATCACTCGAAGCACTCCCAAAGAGATCTCAGTTCGCAGTATGGTTCTCTGAGATCCTAGAGATCTCAGGGGACAAGGAAATCTCAAAGTTAGCAGAGGCCTTGACCAGGACAGATTCCCTTAACCCCCGTTTCCTTCACGACATTCTGGGGGCCACTCTTTATGGATACTGCACGGCTATAACTAGTAAAGTTGACAAGACAGTGACCTTATCTCGGATGGCACTAGCGTCTAAGGATGTTGTTGGAGCTCTAATTAAGGGGGAGATGAGACTTTACTCCTATTTCGGATGGAGAACCTTGCAATCGAGAGGCTTACCTCTGACCACCAGATGTCCCAATAAGTGGGTGCGGATAATTAGGGATATCTCATGGCAAAAACAAATTAAGGCTGTTAGCGTTCCGTATCCTACTCATTTCTTATCAGAGGATATTTCAGAGACTGATAGGCCCGATTCCTGGATTGAGTGCTATATTGATGATGCCCCGACATCTGACAGAAGTTGCATGATCTATTCGACAGGAAAAGCTCTGCCGTACTTGGGTAGTGTCACTAGAGAAAAACTGACAACTCGAGGTGCAAAGGCGGCCTATGGAACTGAACCTCTTGTATTGAGGCCAATTAATCTAGTCAGAACTATAGGTTGGTTCATTGAAGAGGATAGCAATTTTGCTGAATTGATCAAGATGCTACTGGGAGCTGTCACCGATCTTCCCATAGAGGAGGTACTCTATATCCCAGAAATGGTGAGTGGCTCTATGGCACATAGATACCTAGATATGTCAACACAGCATGGATCTTTGTGGATGCCTCTTTATGGTCCTGCCACTTTTCTCCATATGAGCACTAATACTTTTGTGCAGTACCTGAAGGGGACAGAAAATGTCACTTTGCACTTTCAGTGTGTCATGGGCTTAATTCAGTATGCTATAGTCAACAAAGCTCTAGGGGAATGTCCCACAAAGAGAATGACGAGATTCTTCCGATCATGCCCTGACTGTATAGTTCCCATTGATGATACGTTGGAGGACTTGCCTGAGGTCCCTTCGCTAGATCTCATCCCTGAGAGGACCACAAACCCTTATCTCTACCTCAAGAAGGAGAAGATTGAACTGAATGTTAGGCATCGACTTGCAGTGATAAATGAGATCCGGGTAATAGCTAGGGATGAGATAGAAGAGACTCCCCTGTTGGCTAACAATACTCTGGAGGACGTGATGAGTTTGAGGGCAGCTCAGAGAATATTCTACACAGCCAAAGGAAAGGAGGCACAGTGGGACTTGCAGACTGCTGATCGAGAGGGGTACTTAAAACTGGATTTCGTGAGCGTAATCAGGAAGATTATAGGACACCTCTTTGTAATGGAATCAGAGACGTTAAAAGCCGGGTCAGACTACCCTACTTTCCAGTTACAGCAGAGAAGGATCATGCGGCGCGTTAGGCAGTCAGATACATCTAACTTTGTCCACTTAGGAGGGTTCTTCTGCTGGCAAGAGAGCATTGCGAGGATACAGAAACTGAAGTGGTCAGTGATGCCTGCCACATTCCCTATCACTGCAGAATCAGTGTCACTGGCAGCTAAGATGAGTCTAATAGGGGCAATGACTAGCGGGCTAACACCTAAGAGATGTGATGGTGTTCTGGCCGAAAACTTAATCCCGGATGTCACAAGGCAAGCAAAGAACATCATATGCCTGGACAAAGTGTTCCAAAGCAGATGTGATTACTGCTATACTGCAGCAATGACAAATAGGTGGTCAGACTCGATAAACTCCGACAGCATATTCAATATGAGATGTGAGAGAGGCCATACCATTCTGTCCCCGAGAATGTTGCATCAATTGAGGCGAACTATCTTGCCTGAAGGAGCCCTATACACTTTGGCTGTCAGAGTGGTTCACCAAAGCCCTCCTGAAGCATCACAACCTCTTCAGATCATCCCATGCAGGCGAGAGAGATATCAAATCTTGAGTGAAGCTGACATGCCTAGACAAACTGGCTACTCAGGGGTCTATAGAAGGCCTACCAATCAAATCTGCATGTATACAGAGGTTGAATTGGCAAAGAAGTACAGGTTGCCAACTAATTCATTATACAGGATCCTTGACTTACACGACTGCTTCTTCAGTCAGAAACTCATGGACACAAGGGGCAACATTTTAGTGGTCGGAGACGGGTATGGATACAGCTCCTTGCTAACCAAGTGCTTGAACCCAGACAGGAATGTAGTGAGCTGGACCTATATTGAACCATCTGAGGCACTTCCACATAGCCTGAGAATCTCTAAGCCTCCAATGCACTACAAAGCTGATGTGCAGATAGATTCTAGCCCTTCAATTGACAGAATCTCCGACATACATAACTCCTCATACCCGGATGAGTTCGCTAAAGTGGTAACCAAGAATGGGATTACAGCACTGATCAGTGACATTGAAACTGTGTATGTCTCTGGAGAGAAGAGTGTGGCGTCTTTGATCAACTTGGCTTGGAATAACCAGATCCAATTAGGAGCACTCAAGTTGGAATTGATGACGGATCCCTTGGAGAAAGTCGTAGAATATGCCCACAATGCTTACCAAAGATGGGAATTATTCACTCTACCAGGCGCCAATCTCGGAGGAGGGGTACTATACATAGGTTTCTACGGTAGAAGAGAGAAGTTGACTGGGTACATCATTCCCCATTCAGGAGTTGAGACCTTGATGGATAGGTTGGCAACTGAGGTAGATGAGGATAGAGGAAGATTGAGAGCAGAGGACCGTGATCGGTGGGAGCAGTTGGATACAATGGAAAGCAAAATACATCTGCAGCAATACCATAAGATGAGGCTCGATCTATGGTTCGGATCTGCTTATCTGAGTGGTTATCTCTCAGAGGATATGACTGAGTTCTTTTACTCGGTAAAAACATCCTACAGACCACCTGCAATCTCATGGGAGAAAGGGAACCAGGCCAGATACCTCTATGGCAGCAGGGAAGAGATCTTATTCATGAGCATGATCACAGTGGCAGTATCATCCTATCTAGAGGACCAGGATGTCTTAAGAGAGTTCCTTTCTTCCTCAGGGTGGAAACTCCAGTGGAAGAAGGATAAGAAGAATCCGAGGCATTGGTCCCCGTATTTGGAGCGGAGTGACTCTCAACTAGTCTGGAAAAAGAACAAAGCCATGATTTTCAAACTGGTATCAGTTCTGAGAGGATCAAAGCCGAGAGAAGAATATAAGATGGGAAAAGCATCTGACTCCATAGTTTTCAAATACATTCCGAGGAAGGTAAGGGAAGGAGAAGTTCCCCTGTGCTTCCCGATCTCTAAGGTGGCCTCAATGTCGGTATAAGAAAAACGAGACCTTCATCATGGATGATTTAATGGTCTATAGCCTTTGGGCTCTGCTGCAATGGGGTTTGCCTGCGGATACAGCCAGATACATAATCTCACTGAGGATGGAAGAAGACTACTGGGAAGAGATGAATGAACATGAGCAGTGGGACGGGTACGAGTCTTGGGATGATACTGATTGGCAGTATCCGAGTCCTTGAATAAGTCAGTATGTGACAATTTAGAAAAAACGATTACCTTACTGTTGACTTTAAAGAACACTGTTATGTTGTGTTCGCAACACACAACTTCCTT

>Seq6 Rice stripe mosaic virus SG isolate 2, complete genome

AAGGAAGTTGCGTTGCGAACGCAACATTAAAATGTTGATATAACCCACCTTATCATAACAAAAGAAAAACGAGACCGCCAAGTCTTAAAATGGCAACCGACAAGTCTTTTGAGGAGAAGCTGAGCCTGGTTCCTGAGAACACTAAGCTATATTCAATATCTCCTGAGGCATATTCAGATGATAAGTTTGATAAGGCCAATTGCTACAGGCTAGAGAAGAGGTCTGAATATGAGCTAACTAGGCTCTACAAAGGACTAGTCCGGGACTTAGGGAATTCTTCCCCCTCCACTTATGCAGTCGAGAGATTGCTAGTCTTGGCAAGCCATCTGTATGAGACCAAGAAAGGGTCTTCTAACTTCTTCTTGACCGATTACCTGCCCAAGACAACATCAACAGCAAATCTTGATGCAGGCTTTTTGGCAAAGCTGAAAGAGACCCCTAAGGCCTCTGACCCCGATGTTTCCGATGTGACAGAAGTCAAGACAGCTAAGGCTACTCTAGACTCTGCTACAACTGATGCGGATACTAAAAAGGCCGCCTATGAGGCTATCGGAGATGAGGATTCAAAGAAGGCTGAGAAGGCTACAGCAAACACTGCCTGGATTGCTGCCCAGGAGGCCCAGAAGAAGGCTCAGAGTGCCTATGATAAGGCAGTCTCGAATGCGAAGAAGGCCTCAAGGAAAACTACCTCTGGAAAAAGTTTGTTCGGGGATGCAGGGGAGACAGTCACAGATGAGTCTAAGGTAGTGGAAGAGGTTGGAGAGGGGAAGAAGAAGTTTGGACCCTTCTTAGCTGCTTACTTGATGAGGCTACTGACCAAAATAGCATCCAATGTCACAGAGTCATGGGAGCATATGAAAGGAATGTATAAGAACTTTTATGGTTATGATGCTCCTTCAGACCTGAACTGTCCCGAGGCAGGATTCCTTGAGCAACTGAAGTCTGAGCTTAACAAGGATAGAAGGGCGGTTACCTCATGGGTCAAAATAGTAGCAGAGGCAGATAATAAGTTGGATCAATCTACAGCCGAGGCTGGGATTCTCCGCTATGTGGCTGTCCTCCCTCTTGCATATTCTGGGATGCATGCGATGAAGTTGTTCATGGACGTCAAGATGCTGACCAAACTCACCAGCAACTACTTGATTGGTGCCATGAGGAGCCCTCTGACCAAGGATGCATTAGATGCAATCATGGATATCTTGATCTCGTTTGAGTCCACTACAAAGACTAAGAAGTCGGAGAAGTTCCGGTTTGCTAGGATTGTGAGTACTCAATTCTTTCAATCCCTCCAGACCAAGAATTGCAAAGAGCTGGTCTATCTGATGGTTCAGATAATTGCTGAGTACAGGAAAGCAGAGGGGGTCAGGGATCCCATGAACATTGCTGGATTAGATGACATCTCTAGCAGGAACAAGAAGAAGTTGAATAAAGCAGTACGGATAATACTGGCAGAAGCTCCAAAGGCATCCGCAGGTGAGTATTCGTCGGCCATGAAGAAGGCTTTCCTGGATGACGAAGAGGATGATACAGCCAAGACCAGGTCCATCTTCCAGACCAAGGCTTAAGGAACATCATGTTTGGATAGAATATGACTCCTCTAAATAACTATGCAGCCTACTGGCGAGTCTTTGTGTGTTATATAAGAAAAACGAGACCGTCAGGTCAATAATGAGTGTGCCAGAGGATACTCCCTTCAGATCGTACTCCAGCATCTTTGACGACTCAGACTTTGTCCAACCACAGCCCATGTCCTTCAAAGCGACCAAGGAGTCAGAGAGCCTTCCTGAGACAGAGAAAGAAGATATGTCGACTGAATACCTCTCTGAACCACTGAGGACCAAGTCAGGTAAGAAGAACAGGCGGAGGAAGGGAAAGGACCTAAAATCTCTCTTCACTCAAGAAGCTGGACTCCCGGCTCCAGAAGCAGACAGTGTCCTCCCAGAATCGTCTCCGTATGAGAATGATAACGCACAGTTGGAGCTACCCAAGCCCATTCTAAAGACATCAGACGCTCCAGTCTTCCTAAGAGAGAAGGATCTTAGTAAAGAATTTGCTGCAGCCTGCAAGACTAATGGGATTCTCCCAAGGGATGAATGGAAGTCATCTGTAGCAGCCAAGTATCATGCAGAGGAAGGGAAAATGACCAAACGAGACATCTCGTTAATCGTATTTGGAATGGAGCTTTACAAGAGATACAATGTGGAATCTGAGGTATCAACTTTGTTCACTTCATTGGTGACTGAGTTGCAGGGGATAAAGGTTGCTGCTAAGGAGTTGAATGATACCCGGGAGGTCCTCACTAAAATTCCAGGAGAAATTGTGTCTGCTGTCAAGGCAGGGGTAAAAGAGGGGACCGAGATGGGGATGGATTATATAGAGACTAGAACTAAAGTGGCCCCCAAGGGTGCTCCTAAAGTGGACATCTCCAAGCCGATGAGTAGCAAGATGATGGAGCAGCAGGATGAGAGTTCTGATGAGTCCTCAGATAATGAGAGTGAGGAGAGTGAGGAAGAATCCTTTGAGACAAAGGCTGCAATCTTCTTAGCTTTGATAAAGGTTCCAGAGGAAGAGAGAGACAACGCAATAGTCCTTATGGCCTTGAGGGCAGTGATATCTGACAGTGAGCTGAATCAGGCAATTAGGAATGACAGAATCTCCTCCTCAGTAGCAGATATGTACCATCAAAAGATATCTGACAAGGCTAGGGAATTGATGGGAAAGGGAAAGACCAACAAGAGGGCCAAGCAGCCTAAATCCTCTAAGTATGCATCGGATTACTATGATGATGCACTGTGAGTAATCAGTTACTACCTGTGTGGCAATGTCGGAACTGTACTTACCTATATATTGATAATCGTCTGTGATCTTGTGCCTTTAATTGCTGCAGTATTATAGCTTAAAATAATCTAGTTACCAATGCTCTATTTTCGTACTTAAGTGCCATGTTGCCTGCCTTTACCAAAGTGATCTAGTGTGCTCTATTTAAGAAAAACGAGACCTTCATCATGAAGATCATCTGCAGTACTGGGATCTTCAATGAAGAGAAAGGCTTCCCCCTCCCCAACCTCCTCAACAGTCCCTTGATGAAGCAGGAGATCATGACGGTCAAGTACTTGAGGTTTCAGTATATCCCTATTATGACCAGCAACCCCTCGAGTTCATTGACTATTGACATTAAAGATACTCGACTGGTCAATTGGGATAATAGGTCGATTTTCCAGGTCAAAATATTCGGGGATGTTCAAAGCTCATTTATTGTATCGGGTCTACAGCCTTATTCAGCTAGGGACCGTTGCCCTTATCTCCTCTCCCTTTCAGTAAACGCAGGGAAGGTTGTTCCAGGGACCAAATACGGCATCTTAAAATCTTATGCTGTGTACACATCTAAAGACTCAGGGATAATTTCGTCACAGATCTCTGTAAAACTTGAAAGGTCCCCACGTGACTACTTCTTGAAGAGGTCTAAGGAGCATGACAAAAAAGATCTTGACAGTGATGTCTCATTCAAGATGTGTCGCCATGTCAAGTTTGCTACTTGAAGCTTGGAGAGGATGGGCAGCTGAATGTTGTCGGGGTGATGTTTTCCTTATGAATATCTTCAATAATTTCCCTGGAGGGAGAAACAGTTGATCTCTGGGAGGACGCAAGGGCTCACTACTAGCATGTGCTTGCCAGGTTGAGGTTTTATGCCTATCATGGTTTAGCCTAAATAAGAAAAACGAGACCATCATGGCCGTTCCGTGGACTGAGGTTAAAGACTCCAAGTACTTGGCAACTAAGATGTCTGTTACCCTGATCATGGAGATGAATGAAGATACACCTCTAAAGTATCCTTCTTACAACGCATTCGAGAGCATCTTTAAAAGGCTAGCAGAACCAGAAAGCGCAGCACCCCAAGTGGCCGCATGGTTTACTTGGTTCCTAAGAGAGGCAAAGGACATCTACTATCTGGAAGTGAGTAACAAGGAAACTGCCCAATACGGACCAACTAAAGTCTATAAACTTCAGTGTCCAGCCTACCTCTTATCCCGAGTGACAGGAGGATCACAGCTTGATTATACCTCTCTTATAGGTAGCAAGGTGATGACTGAGAAAGATCGGGGGATCCCAGTCAGAACACTCTATATTACTGGAGGGGGTACATCATTCAGAGTCATCAACGAGGAGACAGCCAACCAGTTCATCATAAATGATAATGCTGTCCGCCTACCAGGAGAGTGCAAGGTGGATGGAGGAAGTATAATCTGGAGTTAGAATTTCAAGAAAACTAAACAATAATGAACAAGTGGAACAATCTCGTGTAGTGTATTAGGTGTCCTCTTTAACTAAAGTTAAATAAGAAAAACGAGACCCGCAAGGATCTTAAGATAACAAGATGATGAGGATTTCGGTCTTTCTCTTGATGCTCTGCTGGCTCCCTGTCAGCCTGACCTTCTTCGACAAATCACACATTCCTATAACCACATGTGATAAGAATCTAATGAGTCCTATCCCTTGGAGAACTTACTGCATAGAGGAGTGTGGAATCCGGAATGTAATAGGTGATAAGCTGGATCTGTTTATCTACAATAGGTCTGACAGTGGGAAAGTACAGCTGGCTGACTGCAGGAAGTACAAGATCAGACAGACCTTCACCAAGACTTGGACATTCTCGACATTCAAAGGGGCGATAGAGACAGAGGAGCTAATGCCTAATTATGCAGAGTGTGAATCCACCTGGAGAGATCTGTGCAACTCTGGACCGTGTAGTACCACAACTCCGGTGATCCCTGAGGAATACCATTGGGCTTCTGACACCACGAAGGAGGTCATCTATGTATCTATAGATGCATACCAGAAAACTGTTGCATTCCAGGATCCTAGTGGTGATATCCAGCTCCTAGTCCATGGGGTGATCATAGATGGGAGCCAGTCTGGTTATGTTCAACCCAGCAAAGATCTCATCACTATGTGGGATAAGGTTGAGTTACAAGATGAATGCCCTTGGTCAACGGGAAATTCTCTTTCGTGTTCTACGTCTGATGAGGGAATTTCATACTACTGTGCTGGGAAAGGGCTAGTACTGACCAACATCAGTACGGTGACTGATACCAGATGTGACAACAACCCGCACCTAATGACATCAGGGCACCATGTGATTTTTAGAGTGAAGAAGGCATCAGACCCGAATGCGACTCTCAGCAGGACAGCTCAAATAGTGCTGGACAGGGGGTCAGAAGAGGCCGAGATTGTAGATAGTGTTAATAAGGCGTTGCTGGATAGAGATTCTATCAGGTGTGCAAGCTCATGTCTCGCCTTTGATTACACCATCTCCAAGCCTCAGATGTTTGGCAACCAATTGGCGCTACCTTATAAGGGGTCTTTTCTCCCTTGCAACATACTGCCTAATTGTCGGGTTGTCTTCCCAGTCAAGTATTGCAGCTCTCCTCCGATGATTCTGGTAGAATGTACCGGCACTATGACATGGTGGAATATCACTGGAGATTACACGATCAGACCCACCTATTGCCACATGAACCAGTCGGCGACCAAGATTAAGACATCTATATCCTTTATGACAACAAATGGGAGAGTCTTAGTGAATGAGTCTGGCGCTTATCCTGTCTCCCGTGAAATAGGAAATACGTTCCAGGTCGGACATGTCATAGAGCCTAGCTCCATGATAGAGGTGACTGATCCACTTAATGTTAGGATAGATGACACCTTAGTCACACCAGAGTCCCATACTATATCTAATATCACTTCAGTCGGGGACTCACTTTTGGATACAATGGTTGAGACCGTGAAAGGTATCGGTCGCTTCATATCCCATGAGGTCAGGATAGTGGTTTTTGGTGTTCTGACTCTTTTTATATTATATCTGTCGTTTAAATATTTGTTCGCTAAGAAGAAGAGCAGAGTGCCGCACCCGAAAGTTGTCTATACAAAACCCACATCTGAAGGACCAGTGATCTATGACACCGAATATACTATAGAAAGTGACTAATAAAAAACAGAGACCAACATGGAGTTCAATTGGCCTTGGGGACAGAACAGTGAAACGGAAATCACCAAGAATCTCCGCTTTGAGGACATTAAGGTGATGGCCATAATAATACTAGTCTGGGTGAAGTGTCTTCTCATCTACCATTTCAAGAGGAAAATAAGGCGACTAAGATCTCTATTGATAAAAGGATCCTCACAATGGGTACTGCATGATGCCTAACTCATTAGGAAGATCTGAGATGAGTTTTTATCCTGCCTTATCCTTCTAATTAAGAAAAACGAGACCGCCATCATGGACCTTGATGACGGTGGTCTATGGAGACGTGCTAGGGGTCTGGGGGATTATCACCTGAGGTCTGCTCTGGTGACCCCCTCCTTAGAGCGTTTCCGCAGTCGAAAGGGAAGGCACCGTGAACAACTGTGCTTTGATAGGATGAAATCACTAGGCTGGATGTTGAGGTGGGTAGATCAAGGGAAACTGCTTGGATATCTAATGGTAGAAGCCAACAAATCTTTACCAAAATCCATTGCAAACCAGGAACTCCTAGTTGATACTCTGAAGTTAGAATACGGATGTCTTAGACAGATAATCATGACGGATGGAGACCTCCATGATCAGGTGATCTCTTATCTAGACAGGAAGTCTATCTCTACCCATTACACCCATGGTAGGGAGGTCTTTCAGGAAGCCTTGATAGTTGTCATGGCACTTTCTTCAGGGAGAGAACCACCAGATCATGTCAATAACTTGGGTTATGAAATGCTGAATGAAGAACTTGAAGTGCCCGTGGTCAGAACCTATGGCGTAATCTTCTATCTCTTTGGGGACTTGATCTACGTAAAGTATCCGGAGGAAGAGGGCATGATCACACTGGACATGTTCAGAAACCTGACAGATAAATTCTCTGAGAGGGAGAATATTATGATTGCAACTCAACTAGGAACTGAGATCCTTCAAGAGATATACCCTTCTGAGACAGTGCTCAAAACAGTCTTTTCTCTTTGGGACAAAGGTCTATTGAAAGAGGGAAATGACTTTTACACAGTGGTAAAGACATTCGAAGCAATCATAAATGGGATGTTGATCAAGAACAATGACGGAACATACTATGATCCTTCAGCATACCTCCGAGAAACTGTCATGGGGCTGCCAGTACGACTGAGGGATTATGCACAGACGTTAGTGAGTTACTTAGACTCTCTCCCCTTTAATCCACACCACCTATCTCAGATTGGGGGGTTGTTTCGGTTGTGGGGACACCCCATAGTAGATCCTAACGCTGGGGTCAGAAAGGTAAGGCTTCTGGGAACAGCTGATAAGATGAACCTGACACATATCCCCACCCTAGCAGAGCGGAAATTCAAGGAGATCTTCTACCTGTCGTATTATGAAAAGCATAGAGTCTATCCTAATCATAACTTGAATGGAGAAATTGAAGGAAGCTATTTGCTCTCTCAGCTTGCACAAATGGCTCTGGTTAATCCCAAGCATGCAAATTACTCACTAGTAGACTGGGATTCAGTCAACACACTAGAGACCTTTCCCTTTCCTAAATCATTCAACCTGTCTCTTATTATAGCGGATAAAGCTGTAAGTCCGAACAGAGAAGAATGGTTGGAATTGAGACGTAAGGGAGGAACTCAGATGGATCCGCACATTAGAAGAGGGCCTCTGAAGGCTATGAAAGACGGAGTAATCGATTGTGAGAAACTATTGAGGAAGATAGACCGTAACCCGTCCGGTTTAGCTAAGAAACATAGGATCATTGGACTGTACCCAAAGGAGAGAGAAGAAAATATGGTTCCCAGGATGTTTGCATTAATGTCCTTTGACATGAGAGCTTTCTCAGTGGTCTCTGAATCCATGATAGCAGATCACATCATTCCTCATATAGAAGGTGTCACAATGACGAAGAGCATGTTGGCCCTCCAGAAAGAGATGATAATTTCAACTAAAAGCCAGGCCTCCTCAACCCAGTCAGACAGTATAACCTTCTGTCTTAATATTGACTTTGAAAAGTGGAACCTTAACTTCCGAAGATGGATGACGGAGGGGGTATTCAGAGAAATGGGGCGATTGTTCGGACTTCCAGAGATATTCAATCGGACTTATGACATTTTCAAGAAATCCATCATTTATCTAGCAGATGGAAGCTTCGACCTCTTGCTGACAGATGAGTTGGAAATAGAACCAGGGACCAATCCAGATTGCGCATACACCGGTCATGTTGGGGGGTTTGAGGGATTGAGGCAGAAAGGATGGACAGTCTTCACAGCGGTCTTAATCTCTTCCATCTGCGATGAAATGGGGATCAAGACTCATCTAATGGGGCAAGGTGATAACCAGGTTTTGATGCTGACCATCTACTCAAGAGCTGCTAGAGAGACGGGGGATTTGAAATCAGCACCAGCAGTACTAGAGATCACAAATACATTGGAGACATTTAAGAGCAGACTAGTCTCACTCTTTGCTAATCTTGGATTGCCTATAAAACCACTAGAGACCTGGGTCTCAGAGGAATTGTTTGCCTATGGCAAGACTCCAATATACCGAGCAGTCCCCCTTGCAATGAGTCTCAAGCGAATCTCTAGAGTTTTTGCGTTTTCCAATGAGGATCTAATGACCTTGTCTAATGCGTTAGGTGCTATCTCTGCGAATGCACAAGCTGCATCCATGTGCGATGTCCATCCGATGGTGTCATATGCCATAGCAAAGTGGCAACACCTATGCTGTGCGATAATCTTCTCCAACTATCATCCGCTATGCGGGTGTGCTCCACATGTCTCAGGAGAGGAATGGGCAATAAAGCTACGACTTCCGTCAGGAAAGAAGATTCAAGAGACATCTGATGAGGAGATAGACGAAAGAGATCTGATGAAACTAATAGTGACCATACCTCGAAGCTTAGGAGGTTATAATACCCTGACTCTGTATGAGATGATCATGAGAGGATTTTCTGACCCAGTCTCTAGGGACATGTGCTGGCTATTCGCAATTGCCAGTGAATCAACAGGAAAACTTCGAGGTTATCTGATAAACTGGATTAAACCAATCGTGTCTCCGGAAGTAAATGCTCAGCATCTCATTCAGGACCCCACGGCACTCAATTTGCTAGTACCCCCTAATGCTACATCAGTTATTAAGAGGATGATAGACAAATCACTCGAAGCACTCCCAAAGAGATCTCAGTTCGCAGTATGGTTCTCTGAGATCCTAGAGATCTCAGGGGACAAGGAAATCTCAAAGTTAGCAGAGGCCTTGACCAGGACAGATTCCCTGAACCCCCGTTTCCTTCACGACATTCTGGGGGCCACTCTTTATGGATACTGCACGGCTATAACTAGTAAAGTTGACAAGACAGTGACCTTATCTCGGATGGCACTAGCGTCTAAGGATGTTGTTGGAGCTCTAATCAAGGGGGAGATGAGACTTTACTCCTATTTCGGATGGAGAACCTTGCAATCAAGAGGCTTACCTCTGACCACCAGATGTCCCAATAAGTGGGTGCGGATAATTAGGGATATCTCATGGCAAAAACAAATTAAGGCTGTTAGCGTTCCGTATCCTACTCATTTCTTATCAGAGGATATTTCAGAGACTGATAGGCCCGATTCCTGGATTGAGTGCTATATTGATGATGCCCCGACATCTGACAGAAGTTGCATGATCTATTCGACAGGAAAAGCTCTGCCGTACTTGGGTAGTGTCACTAGAGAAAAACTGACAACTCGAGGTGCAAAGGCGGCCTATGGAACTGAACCTCTCGTATTGAGGCCAATTAATCTAGTCAGAACTATAGGTTGGTTCATTGAAGAGGATAGCAATTTTGCTGAATTGATCAAGATGCTACTGGGAGCCGTCACCGATCTTCCCATAGAGGAGGTACTCTATATCCCAGAAATGGTGAGTGGCTCTATGGCACATAGATACCTAGATATGTCAACACAGCATGGATCTTTGTGGATGCCTCTTTATGGTCCTGCCACTTTTCTCCATATGAGCACTAATACTTTTGTGCAGTACCTGAAGGGGACAGAAAATGTCACTTTGCACTTTCAGTGTGTCATGGGCTTAATTCAGTATGCTATAGTCAACAAAGCTCTAGGGGAATGTCCCACAAAGAGAATGACGAGATTCTTCCGATCATGCCCTGACTGTATAGTTCCCATTGATGATACGTTGGAGGACTTGCCTGAGGTCCCTTCGCTAGATCTCATCCCTGAGAGGACCACAAACCCTTATCTCTACCTCAAGAAGGAGAAGATTGAACTGAATGTTAGGCATCGACTTGCAGTGATAAATGAAATCCGGGTAATAGCTAGGGATGAGATAGAAGAGACTCCCCTGTTGGCTAACAATACTCTGGAGGACGTGATGAGTTTGAGGGCAGCTCAGAGAATATTCTACACAGCCAAAGGAAAGGAGGCACAGTGGGACTTGCAGACTGCTGATCGAGAGGGGTACTTAAAACTGGATTTCGTGAGCGTAATCAGGAAGATTATAGGACACCTCTTTGTAATGGAATCAGAGACGTTAAAAGCCGGGTCAGACTACCCTACTTTCCAGTTACAGCAGAGAAGGATCATGCGGCGCGTTAGGCAGTCAGATACATCTAACTTTGTCCACTTAGGAGGGTTCTTCTGCTGGCAAGAGAGCATTGCGAGGATACAGAAACTGAAGTGGTCAGTGATGCCTGCCACATTCCCTATCACTGCAGAATCAGTGTCACTGGCAGCTAAGATGAGTCTAATAGGGGCAATGACTAGCGGGCTAACACCTAAGAGATGTGATGGTGTTCTGGCCGAAAACTTAATCCCGGATGTCACAAGGCAAGCAAAGAACATCATATGCCTGGACAAAGTGTTCCAAAGCAGATGTGATTACTGCTATACTGCAGCAATGACAAATAGGTGGTCAGACTCGATAAACTCCGACAGCATATTCAATATGAGATGTGAGAGAGGCCATACCATTCTGTCCCCGAGAATGTTGCATCAATTGAGGCGAACTATCTTGCCTGAAGGAGCCCTATACACTTTGGCTGTCAGAGTGGTTCACCAAAGCCCTCCTGAAGCATCACAACCTCTTCAGATCATCCCATGCAGGCGAGAGAGATATCAAATCTTGAGTGAAGCTGACATGCCTAGACAAACTGGCTACTCGGGGGTCTATAGAAGGCCTACCAATCAAATCTGCATGTATACAGAGGTTGAATTGGCAAAGAAGTACAGGTTGCCAACTAATTCATTATACAGGATCCTTGACTTACACGACTGCTTCTTCAGTCAGAAACTCATGGACACAAGGGGCAACATTTTAGTGGTCGGAGACGGGTATGGATACAGCTCCTTGCTAACCAAGTGCTTGAACCCAGACAGGAATGTAGTGAGCTGGACCTATATTGAACCATCTGAGGCACTTCCACATAGCCTGAGAATCTCTAAGCCTCCAATGCACTACAAAGCTGATGTGAAGATAGATTCTAGCCCTTCAATTGACAGAATCTCCGACATACATAACTCCTCATACCCGGATGAGTTCGCTAAAGTGGTAACCAAGAATGGGATTACAACACTGATCAGTGACATTGAAACTGTGTATGTCTCTGGAGAGAAGAGTGTGGCGTCTCTGATCAACTTGGCTTGGAACAACCAGATCCAATTAGGAGCACTCAAGTTGGAATTGATGACAGATCCCTTGGAGAAAGTCGTAGAATATGCCCACAATGCTTACCAAAGATGGGAATTATTCACTCTACCAGGCGCCAATCTCGGAGGAGGGGTACTATACATAGGTTTCTACGGTAGAAGAGAGAAGTTGACTGGGTACATCATTCCCCATTCAGGAGTTGAGACCTTGATGGATAGGTTGGCAACTGAGGTAGATGAGGATAGAGGAAGATTGAGAGCAGAGGACCGTGATCGGTGGGAGCAGTTGGATACAATGGAAAGCAAAATACATCTGCAGCAATACCATAAGATGAGGCTCGACCTATGGTTCGGATCTGCTTATCTGAGTGGTTATCTCTCAGAGGATATGACTGAGTTCTTTTACTCGGTAAAAACATCCTACAGACCACCTGCAATCTCATGGGAGAAAGGGAACCAGGCCAGATACCTCTATGGCAGCAGGGAAGAGATCTTGTTCATGAGCATGATCACAGTGGCACTATCATCCTATCTAGAGGACCAGGATGTCTTAAGAGAGTTCCTTTCTTCATCAGGGTGGAAACTCCAGTGGAAGAAGGATAAGAAGAATCCGAGGCATTGGTCCCCGTATTTGGAGCGGAGTGACTCTCAACTAGTCTGGAAAAAGAACAAAGCCATGATTTTCAAACTGGTATCAGTTCTGAGAGGATCAAAGCCGAGAGAAGAATATAAGATGGGAAAAGCATCTGACTCCATAGTTTTCAGATACATTCCGAGGAAGGTAAGGGAAGGAGAAGTTCCCCTGTGCTTCCCGATCTCTAAGGTGGCCTCAATGTCGGTATAAGAAAAACGAGACCTTCATCATGGATGATTTAATGGTCTATAGCCTTTGGGCTCTGCTGCAATGGGGTTTGCCTGCGGATACAGCCAGATACATAATCTCACTGAGGATGGAAGAAGACTACTGGGAAGAGATGAATGAACATGAGCAGTGGGACGGGTACGAGTCTTGGGATGATACTGATTGGCAGTATCCGAGTCCTTGAATAAGTCAGTATGTGACAATTTAGAAAAAACGATTACCTTACTGTTGACTTTAAAGAACACTGTTATGTTGTGTTCGCAACACACAACTTCCTT

>Seq7 Rice stripe mosaic virus WZ isolate 9, complete genome

AAGGAAGTTGCGTTGCGAACGCAACATTAAAATGTTGACATAACCCACCTTATCATAACAAAAGAAAAACGAGACCGCCAAGTCTTAAAATGGCAACCGACAAGTCTTTTGAGGAGAAGCTGAGCCTGGTTCCTGAGAACACTAAGCTATATTCAATATCTCCTGAGGCATATTCAGATGATAAGTTTGATAAGGCCAATTGCTACAAGCTAGAGAAGAGGTCTGAATATGAGCTAACTAGGCTCTACAAAGGACTAGTCCGGGACTTAGGGAATTCTTCCCCCTCCACTTATGCAGTCGAGAGATTGCTAGTCTTGGCAAGCCATCTGTATGAGACCAAGAAAGGGTCTTCTAACTTCTTCTTGACCGATTACCTGCCCAAGACAACATCAACAGCAAATCTTGATGCAGGCTTTTTGGCAAAGCTGAAAGAGACCCCTAAGGCCTCTGACCCCGACGTTTCCGATGTGACAGAAGTCAAGACAGCTAAGGCTACTCTAGACTCTGCTACAACTGATGCGGATACTAAAAAGGCCGCTTATGAGGCTATCGGAGATGAGGACTCGAAGAAGGCTGAGAAGGCTACAGCAAACACTGCCTGGATTGCTGCCCAAGAGGCCCAGAAGAAGGCTCAGAGTGCCTATGATAAGGCAGTTTCGAATGCGAAGAAGGCCTCAAGGAAAACTACCTCTGGAAAAAGCTTGTTCGGGGATGCAGGGGAGACAGTCACAGATGAGTCTAAGGTAGTGGAAGAGGTTGGAGAGGGGAAGAAGAAGTTTGGACCCTTCTTAGCTGCTTACTTGATGAGGCTGCTGACTAAAATAGCATCCAATGTCACAGAGTCATGGGAGCATATGAAAGGAATGTATAAGAACTTTTATGGTTATGATGCTCCTTCAGACCTGAACTGTCCCGAGGCAGGATTCCTTGAGCAACTGAAGTCTGAGCTTAACAAGGATAGAAGGGCGGCTACCTCATGGGTCAAAATAGTAGCAGAGGCAGATAATAAGTTGGATCAATCTACAGCCGAGGCTGGGATTCTCCGCTATGTGGCTGTCCTCCCTCTTGCATATTCTGGGATGCATGCGATGAAGTTGTTCATGGATGTCAAGATGCTGACCAAACTCACCAGCAACTACTTGATTGGTGCCATGAGGAGCCCTCTGACCAAGGATGCATTAGATGCAATCATGGATATCTTGATCTCGTTTGAGTCCACTACAAAGACTAAGAAGTCGGAGAAGTTCCGGTTTGCTAGGATTGTGAGTACTCAGTTCTTTCAATCCCTCCAGACCAAGAATTGCAAAGAGCTGGTCTATCTGATGGTTCAGATAATTGCTGAGTACAGGAAAGCAGAGGGGGTCAGGGATCCCATGAACATTGCTGGATTAGATGACATCTCTAGCAGGAACAAGAAGAAGTTGAATAAAGCAGTACGGATAATACTGGCAGAAGCTCCAAAGGCATCCGCAGGTGAGTATTCGTCAGCCATGAAGAAGGCTTTCCTGGATGACGAAGAGGATGATACAGCCAAGACCAGGTCCATCTTCCAGACCAAGGCTTAAGGAACATCATGTTTGGATAGAATATGACTCCTCTAAATAACTATGCAGCCTACTGGCGAGTCTTTGTGTGTTATATAAGAAAAACGAGACCGTCAGGTCAATAATGAGTGTGCCAGAGGATACTCCCTTCAGATCGTACTCCAGCATCTTTGACGACTCAGACTTTGTCCAACCACAGCCCATGTCCTTCAAAGCGACCAAGGAGTCAGAGAGCCTTCCTGAGACAGAGAAAGAAGATATGTCGACTGAATACCTCTCTGAACCACTGAGGACCAAGTCAGGTAAGAAGAACAGACGGAGGAAGGGAAAAGACCTAAAATCTCTCTTCACTCAAGAAGCTGGACTCCCGGCTCCAGAAGCAGACAGTGTCCTCCCCGAATCGTCTCCGTATGAGAATGATAACGCACAGTTGGAGCTACCCAAGCCCATTCTAAAGACATCAGACGCTCCGGTCTTCCTAAGAGAGAAGGATCTTAGTAAAGAATTTGCTGCAGCCTGCAAGACTAATGGGATTCTCCCAAGGGATGAATGGAAGTCATCTGTAGCAGCCAAGTATCATGCAGAGGAAGGGAAAATGACCAAACGAGACATCTCGTTAATCGTATTTGGAATGGAGCTTTACAAGAGATACAATGTGGAATCTGAGGTATCAACTTTGTTCACTTCATTGGTGACTGAGTTGCAGGGGATAAAGGTTGCTGCTAAGGAGTTGAATGATACCCGGGAGGTCCTCACTAAGATTCCAGGAGAAATTGTGTCTGCTGTCAAGGCAGGGGTAAAGGAGGGGACCGAGATGGGGATGGATTATATAGAGACTAGAACTAAAGTGGCCCCCAAAAGTGCTCCTAAAGTGGACATCTCCAAGCCGATGAGTAGTAAGATGATGGAGCAGCAGGATGAGAGTTCTGATGAGTCCTCAGATAATGAGAGTGAGGAGAGTGAGGAAGAATCCTTTGAGACAAAGGCTGCAATCTTCTTAGCTTTGATAAAGGTTCCAGAGGAAGAGAGAGACAACGCAATAGTCCTTATGGCCTTGAGGGCAGTGATATCTGACAGTGAGCTGAATCAGGCAATTAGGAATGACAGAATCTCCTCCTCAGTAGCAGATATGTACCATCAAAAGATATCTGACAAGGCTAGGGAATTGATGGGAAAGGGAAAGACCAACAAGAGGGCCAAGCAGCCTAAATCCTCTAAGTATGCATCGGATTACTATGATGATGCACTGTGAGTAATCAGTTACTACCTGTGTGGCAATGTCGGAACTGTACTTACCTATATATTGATAATCGTCTGTGATCTTGTGCCTTTAATTGCTGCAGTATTATAGCTTAAAATAATCTAGTTACCAATGCTCTATTTTCGTACTTAAATGCCATGTTGCCTGCCTTTACCAAAGTAATCTAGTGTGCTCTATTTAAGAAAAACGAGACCTTCATCATGAAGATCATCTGCAGTACTGGGATCTTCAATGAAGAGAAAGGCTTCCCCCTCCCCAACCTCCTCAACAGTCCCTTGATGAAGCAGGAGATCATGACGGTCAAGTACTTGAGGTTTCAGTATATCCCTATTATGACCAGCAACCCCTCGAGTTCATTGACTATTGACATTAAAGATACTCGACTGGTCAATTGGGATAATAGGTCGATTTTCCAGGTCAAAATATTCGGGGATGTTCAAAGCTCATTTATTGTATCGGGTCTACAGCCTTATTCAGCTAGAGACCGTTGCCCTTATCTCCTCTCCCTTTCAGTAAACGCAGGGAAGGTTGTTCCAGGGACCAAATACGGCATCTTAAAATCTTATGCTGTGTACACATCTAAAGACTCAGGGATAATTTCGTCACAGATCTCTGTAAAACTTGAAAGGTCCCCACGTGACTACTTCTTGAAGAGGTCTAAGGAGCACGACAAAAAAGATCTTGACAGTGATGTCTCATTCAAGATGTGTCGCCATGTCAAGTTTGCTACTTGAAGCTTGGAGAGGATGGGCAGCTGAATGTTGTCGGGGTGATGTTTTCCTTATGAATATCGTCAATAATTTCCCTGGAGGGAGAAACAGTTGATCTCTGGGAGGACGCAAGGGCTCACTACTAGCATGTGCTTGCAAGGTTGAGGTTTTATGCCTATCATGGTTTAGCCTAAATAAGAAAAACGAGACCATCATGGCCGTTCCGTGGACTGAGGTTAAAGACTCCAAGTACTTGGCAACTAAGATGTCTGTTACCCTGATCATGGAGATGAATGAAGATACACCTCTAAAGTATCCTTCTTACAACGCATTCGAGAGCATCTTTAAAAGGCTAGCAGAACCAGAAAGCGCAGCACCCCAAGTGGCCGCATGGTTTACTTGGTTCCTAAGAGAGGCAAAGGACATCTACTATCTGGAAGTGAGTAACAAGGAAACTGCCCAATACGGACCAACTAAAGTCTATAAACTTCAGTGTCCAGCCTACCTCTTATCCCGAGTGACAGGAGGATCACAGCTTGATTATACCTCTCTTATAGGTAGCAAGGTGATGACTGAGAAAGATCGGGGGATCCCAGTCAGAACACTCTATATTACTGGAGGGGGTACATCATTCAGAGTCATCAACGAGGAGACAGCCAACCAGTTCATCATAAATGATAATGCTGTCCGCCTACCAGGAGAGTGCAAGGTGGATGGAGGAAGTATAATCTGGAGTTAGAATTTCAAGAAAACTAAACAATAATGAACAAGTGGAACAATCTCGTGTAGTGTATTAGGTATCCTCTTTAACTAAAGTTAAATAAGAAAAACGAGACCCGCAAGGATCTTAAGATAACAAGATGATGAGGATTTCGGTCTTTCTCTTGATGCTCTGCTGGCTCCCTGTCAGCCTGACCTTCTTCGACAAATCACACATTCCTATAACCACATGTGATAAGAATCTAATGAGTCCTATCCCTTGGAGAACTTACTGCATAGAGGAGTGTGGAATCCGGAATGTAATAGGTGATAAGCTGGATCTGTTTATCTACAATAGGTCTGACAGTGGGAAAGTACAGCTGGCTGACTGCAGGAAGTACAAGATCAGACAGACCTTCACCAAGACTTGGACATTCTCGACATTCAAAGGGGCGATAGAGACAGAGGAGCTAATGCCTAATTATGCAGAGTGTGAATCCACCTGGAGAGATCTGTGCAACTCCGGACCGTGTAGTACCACAACTCCGGTGATCCCTGAGGAATACCATTGGGCCTCTGACACCACGAAGGAGGTCATCTATGTATCTATAGATGCATACCAGAAAACTGTTGCATTCCAGGATCCTAGTGGTGATATCCAGCTCCTAGTCCATGGGGTGATCATAGATGGGAGCCAGTCTGGTTATGTTCAACCCAGCAAAGATCTCATCACTATGTGGGATAAGGTTGAGTTACAAGATGAATGCCCTTGGTCAACGGGAAATTCTCTTTCGTGTTCTACATCTGATGAGGGAATTTCATACTACTGTGCTGGGAAAGGGCTAGTACTGACCAACATCAGTACGGTGACTGATACCAGATGTGACAACAACCCGCACCTAATGACATCAGGGCACCATGTGATTTTTAGAGTGAAGAAGGCATCAGACCCGAATGCGACTCTCAGCAGGACAGCTCAAATAGTGCTGGACAGGGGGTCAGAAGAGGCCGAGATTGTAGATAGTGTTAATAAGGCGTTGCTGGATAGAGATTCCATCAGGTGTGCAAGCTCATGTCTCGCCTTTGATTACACCATCTCCAAGCCTCAGATGTTTGGCAACCAATTGGCGCTACCTTATAAGGGGTCTTTTCTCCCTTGCAACATACTGCCTAATTGTCGGGTTGTCTTCCCAGTCAAGTATTGCAGCTCTCCTCCGATGATTCTGGTAGAATGTACCGGCACTATGACATGGTGGAATATCACTGGAGATTACACGATCAGACCCACCTATTGCCACATGAACCAGTCGGCGACCAAGATTAAGACATCTATATCCTTTATGACAACAAATGGGAGAGTCTTAGTGAATGAGTCTGGCGCTTATCCTGTCTCCCGTGAAATAGGAAATACGTTCCAGGTCGGACATGTCATAGAGCCTAGCTCCATGATAGAGGTGACTGATCCACTGAATGTTAGGATAGATGACACCTTAGTCACACCAGAGTCCCATACTATATCTAATATCACTTCAGTCGGGGACTCACTTTTGGATACAATGGTTGAGACCGTGAAAGGTATCGGTCGCTTCATATCCCATGAGGTCAGGATAGTGGTTTTTGGTGTTCTGACTCTTTTTATATTATATCTGTCGTTTAAATATTTGTTCGCTAAGAAGAAGAGCAGAGTGCCGCACCCTAAAGTTGTCTATACAAAACCCACATCTGAAGGACCAGTGATCTATGACACTGAATATACTATAGAAAGTGACTAATAAAAAACAGAGACCAACATGGAGTTCAACTGGCCTTGGGGACAGAACAGTGAAACGGAAATCACCAAGAATCTCCGCTTTGAGGACATTAAGGTGATGGCCATAATAATACTAGTCTGGGTGAAGTGTCTTCTCATCTACCATTTCAAGAGGAAAATAAGGCGACTAAGATCTCTATTGATAAAAGGATCCTCACAATGGGTACTGCATGATGCCTAACTCATTAGGAAGATCTGAGATGAGTTTTTATCCTGCCTTATCCTTCTAATTAAGAAAAACGAGACCGCCATCATGGACCTTGATGACGGTGGTCTATGGAGACGTGCTAGGGGTCTGGGGGATTATCACCTGAGGTCTGCTCTGGTGACCCCCTCCTTAGAGCGTTTCCGCAGTCGAAAGGGAAGGCACCGTGAACAACTGTGCTTTGATAGGATGAAATCACTAGGCTGGATGTTGAGGTGGGTAGATCAAGGGAAACTGCTTGGATATTTAATGGTAGAAGCCAACAAATCTTTACCAAAATCCATTGCAAACCAGGAACTCCTAGTTGAGACTCTGAAGTTGGAATACGGATGTCTTAGACAGATAATCATGACGGATGGAGACCTCCATGATCAGGTGATCTCTTATCTAGACAGGAAGTCTATCTCTACCCATTACACCCATGGTAGGGAGGTCTTTCAGGAAGCCTTGATAGTTGTCATGGCACTTTCTTCAGGGAGAGAACCACCAGATCATGTCAATAACTTGGGTTATGAAATGCTGAATGAAGAACTTGAAGTGCCCGTGGTCAGAACCTATGGCGTAATCTTCTATCTCTTTGGGGACTTGATCTACGTAAAGTATCCGGAGGAAGAGGGCATGATCACACTGGACATGTTCAGAAACCTGACAGATAAATTCTCTGAGAGGGAGAATATTATGATTGCAACTCAACTAGGAACTGAGATCCTTCAAGAGATATACCCTTCTGAGACAGTGCTCAAAACAGTCTTTTCTCTTTGGGACAAAGGTCTATTGAAAGAGGGAAATGACTTTTACACAGTGGTAAAGACATTCGAAGCAATCATAAATGGGATGTTGATCAAGAACAATGACGGAACATACTATGATCCTTCAGCATACCTCCGAGAAACTATCATGGGGCTGCCAGTACGACTGAGGGATTATGCACAGACGTTAGTGAGTTACTTAGACTCTCTCCCCTTTAATCCACACCACCTATCTCAGATTGGGGGGTTGTTTAGGTTGTGGGGACACCCCATAGTAGATCCTAACGCTGGGGTCAGAAAGGTAAGGCTTCTGGGAACAGCTGATAAGATGAACCTGACACATATCCCCACCCTAGCAGAGCGGAAATTCAAGGAGATCTTCTACCTGTCGTATTATGAAAAGCATAGAGTCTATCCTAATCATAACTTGAATGGAGAAATTGAAGGAAGCTATTTGCTCTCTCAGCTTGCACAAATGGCTCTGGTTAATCCCAAGCATGCAAATTACTCACTAGTAGACTGGGATTCAGTCAACACACTAGAGACCTTTCCCTTTCCTAAATCATTCAACCTGTCTCTTATTATAGCGGATAAAGCTGTAAGTCCGAACAGAGAAGAATGGTTGGAATTGAGACGTAAGGGAGGAACTCAGATGGATCCGCACATTAGAAGAGGGCCTCTGAAGGCTATGAAAGACGGAGTAATCGATTGTGAGAAACTATTGAGGAAGATAGACCGTAACCCGTCCGGTTTAGCTAAGAAACATAGGATCATTGGACTGTACCCAAAGGAGAGAGAAGAAAATATGGTTCCCAGGATGTTTGCATTAATGTCCTTTGACATGAGAGCTTTCTCAGTGGTCTCTGAATCCATGATAGCAGATCACATCATTCCTCATATAGAAGGTGTCACAATGACGAAGAGCATGTTGGCCCTCCAGAAAGAGATGATCATTTCAACTAAAAGCCAGGCCTCCTCAACCCAGTCAGACAGTATAACCTTCTGTCTTAATATTGACTTTGAAAAGTGGAACCTTAACTTCCGAAGATGGATGACGGAGGGGGTATTCAGAGAAATGGGGCGATTGTTCGGACTTCCAGAGATATTCAATCGGACTTATGACATCTTCAAGAAATCCATCATTTATCTAGCAGATGGAAGCTTCGACCTCTTGCTGACAGATGAGTTGGAAATAGAACCAGGGACCAATCCAGATTGCGCATACACCGGTCATGTTGGGGGGTTTGAGGGATTGAGGCAGAAAGGATGGACAGTCTTCACAGCGGTCTTAATCTCTTCCATCTGCGATGAAATGGGGATCAAGACTCATCTAATGGGGCAAGGTGATAACCAGGTTTTGATGCTGACCATCTACTCAAGAGCTGCTAGAGAGACGGGGGATTTGAAATCAGCACCAGCAGTACTGGAGATCACAAATACATTGGAGACATTCAAGAGCAGACTAGTCTCACTCTTTGCTAATCTTGGATTGCCTATAAAACCACTAGAGACCTGGGTCTCAGAGGAATTGTTTGCCTATGGCAAGACTCCAATATACCGAGCAGTCCCCCTTGCAATGAGTCTCAAGCGAATCTCTAGAGTTTTTGCGTTCTCCAATGAGGATCTAATGACCTTGTCTAATGCGTTAGGTGCTATCTCTGCGAACGCACAAGCTGCATCCATGTGCGATGTCCATCCGATGGTGTCATATGCCATAGCAAAGTGGCAACACTTATGCTGTGCGATAATCTTCTCCAACTATCATCCGCTATGCGGGTGTGCTCCACATGTCTCAGGAGAGGAATGGGCAATAAAGCTACGACTTCCGTCAGGAAAGAAGATTCAAGAGACATCTGATGAGGAGATAGACGAAAGAGATCTGATGAAACTAATAGTGACCATACCTCGAAGCTTAGGAGGTTATAATACCCTGACTCTGTATGAGATGATCATGAGAGGATTTTCTGACCCAGTCTCTAGGGACATGTGCTGGCTATTCGCAATTGCCAGTGAATCAACAGGAAAACTTCGAGGTTATCTGATAAACTGGATTAAACCAATCGTGTCTCCGGAAGTAAATGCTCAGCATCTCATTCAGGATCCCACGGCACTCAATTTGCTAGTACCCCCTAATGCTACATCAGTTATTAAGAGGATGATAGACAAATCACTCGAAGCACTCCCAAAGAGATCTCGGTTCGCAGTATGGTTCTCTGAGATCCTAGAGATCTCAGGGGACAAGGAAATCTCAAAGTTAGCAGAGGCCTTGACCAGGACAGATTCCCTGAACCCCCGTTTCCTTCACGACATTCTGGGGGCCACTCTTTATGGATACTGCACGGCTATAACTAGTAAAGTTGACAAGACAGTGACCTTATCTCGGATGGCACTAGCGTCTAAGGATGTTGTTGGAGCTCTAATCAAGGGGGAGATGAGACTTTACTCCTATTTCGGATGGAGAACCTTGCAATCGAGAGGCTTACCTCTGACCACCAGATGTCCCAATAAGTGGGTGCGGATAATTAGGGATATCTCATGGCAAAAACAAATTAAGGCTGTTAGCGTTCCGTATCCTACTCATTTCTTATCAGAGGATATTTCAGAGACTGATAGGCCCGATTCCTGGATTGAGTGCTATATTGATGATGCCCCGACATCTGACAGAAGTTGCATGATCTATTCGACAGGAAAAGCTCTGCCGTACTTGGGTAGTGTCACTAGAGAAAAACTGACAACTCGAGGTGCAAAGGCGGCCTATGGAACTGAACCTCTTGTATTGAGGCCAATTAATCTAGTCAGAACTATAGGTTGGTTCATTGAAGAGGATAGCAATTTTGCTGAATTGATCAAGATGCTACTGGGAGCCGTCACAGATCTTCCCATAGAGGAGGTACTCTATATCCCAGAAATGGTGAGTGGCTCTATGGCACATAGATACCTAGATATGTCAACACAGCATGGATCTTTGTGGATGCCTCTTTATGGTCCTGCCACTTTTCTCCATATGAGCACTAATACTTTTGTGCAGTACCTGAAGGGGACAGAAAATGTCACTTTGCACTTTCAGTGTGTCATGGGCTTAATTCAGTATGCTATAGTCAACAAAGCTCTAGGGGAATGTCCCACAAAGAGAATGACGAGATTCTTCCGATCATGCCCTGACTGTATAGTTCCCATTGATGATACGTTGGAGGACTTGCCTGAGGTCCCTTCGCTAGATCTCATCCCTGAGAGGACCACAAACCCTTATCTCTACCTCAAGAAGGAGAAGATTGAACTGAATGTTAGGCATCGACTTGCAGTGATAAATGAGATCCGGGTAATAGCTAGGGATGAGATAGAAGAGACTCCCCTCTTGGCTAACAATACTCTGGAGGACGTGATGAGTTTGAGGGCAGCTCAGAGAATATTCTACACAGCCAAAGGAAAGGAGGCACAGTGGGACTTGCAGACTGCTGATCGAGAGGGGTACTTAAAACTGGATTTCGTGAGCGTAATCAGGAAGATTATAGGACACCTCTTTGTAATGGAATCAGAGACGTTAAAAGCCGGGTCAGACTACCCTACTTTCCAGTTACAGCAGAGAAGGATCATGCGGCGCGTTAGGCAGTCAGATACATCTAACTTTGTCCACTTAGGAGGGTTCTTCTGCTGGCAAGAGAGCATTGCGAGGATACAGAAACTGAAGTGGTCAGTGATGCCTGCCACATTCCCTATCACTGCAGAATCAGTGTCACTGGCAGCTAAGATGAGTCTAATAGGGGCAATGACTAGCGGGCTAACACCTAAGAGATGTGATGGTGTTCTGGCCGAAAACTTAATCCCGGATGTCACAAGGCAAGCAAAGAACATCATATGCCTGGACAAAGTGTTCCAAAGCAGATGTGATTACTGCTATACTGCAGCAATGACAAATAGGTGGTCAGACTCGATAAACTCCGACAGCATATTCAATATGAGATGTGAGAGAGGCCATACCATTCTGTCCCCGAGAATGTTGCATCAATTGAGGCGAACTATCTTGCCTGAAGGAGCCCTATACACTTTGGCTGTCAGAGTGGTTCACCAAAGCCCTCCTGAAGCATCACAACCTCTTCAGATCATCCCATGCAGGCGAGAGAGATATCAAATCTTGAGTGAAGCTGACATGCCTAGACAAACTGGCTACTCGGGGGTCTATAGAAGGCCTACCAATCAAATCTGCATGTATACAGAGGTTGAATTGGCAAAGAAGTACAGGTTGCCAACTAATTCATTATACAGGATCCTTGACTTACACGACTGCTTCTTCAGTCAGAAACTCATGGACACAAGGGGCAACATTTTAGTGGTCGGAGACGGGTATGGATACAGCTCCTTGCTAACCAAGTGCTTGAACCCAGACAGGAATGTAGTGAGCTGGACCTATATTGAACCATCTGAGGCACTTCCACATAGCCTGAGAATCTCTAAGCCTCCAATGCACTACAAAGCTGATGTGCAGATAGATTCTAGCCCTTCAATTGACAGAATCTCCGACATACATAACTCCTCATACCCGGATGAGTTCGCTAAAGTGGTGACCAAGAATGGGGTTACAGCACTGATCAGTGACATTGAAACTGTGTATGTCTCTGGAGAGAAGAGTGTGGCGTCTTTGATCAACTTGGCTTGGAATAACCAGATCCAATTAGGAGCACTCAAGTTGGAATTGATGACGGATCCCTTGGAGAGAGTCGTAGAATATGCCCACAATGCTTACCAAAGATGGGAATTATTCACTCTACCAGGCGCCAATCTCGGAGGAGGGGTACTATACATCGGTTTCTACGGTAGAAGAGAGAAGTTGACTGGGTACATCATTCCCCATTCAGGAGTTGAGACCTTGATGGATAGGTTGGCAACTGAGGTAGATGAGGATAGAGGAAGATTGAGAGCAGAGGACCTTGATCGGTGGGAGCAGTTGGATACAATGGAAAGCAAAATACATCTGCAGCAATACCATAAGATGAGGCTCGATCTATGGTTCGGATCTGCTTATCTGAGTGGTTATCTCTCAGAGGATATGACTGAGTTCTTTTACTCAGTAAAAACATCTTACAGACCACCTGCAATCTCATGGGAGAAAGGGAACCAGGCCAGATACCTCTATGGCAGCAGGGAAGAGATCTTATTCATGAGCATGATCACAGTGGCACTATCATCATATCTAGAGGACCAGGATGTCTTAAGAGAGTTCCTTTCTTCCTCAGGGTGGAAACTCCAGTGGAATAAGGATAAGAAGAATCCGAGGCATTGGTCCCCGTATTTGGAGCGGAGTGACTCTCAACTAGTCTGGAAAAAGAACAAAGCCATGATTTTCAAACTGGTATCAGTTCTGAGAGGATCAAAGCCGAGAGAAGAATATAAGATGGGAAAAGCATCTGACTCCATAGTTTTCAGATACATTCCGAGGAAGGTAAGGGAAGGAGAAGTTCCCCTGTGCTTCCCGATCTCTAAGGTGGCCTCAATGTCGGTATAAGAAAAACGAGACCTTCATCATGGATGATTTAATGGTCTATAGCCTTTGGGCTCTGCTGCAATGGGGTTTGCCTGCGGATACAGCCAGATACATAATCTCACTGAGGATGGAAGAAGACTACTGGGAAGAGATGAATGAACATGAGCAGTGGGACGGGTACGAGTCTTGGGATGATACTGATTGGCAGTATCCGAGTCCTTGAATAAGTCAGTATGTGACAATTTAGAAAAAACGATTACCTTACTGTTGACTTTAAAGAACACTGTTATGTTGTGTTCGCAACACACAACTTCCTT

>Seq8 Rice stripe mosaic virus WZ isolate 12, complete genome

AAGGAAGTTGCGTTGCGAACGCAACATTAAAATGTTGACATAACCCACCTTATCATAACAAAAGAAAAACGAGACCGCCAAGTCTTAAAATGGCAACCGACAAGTCTTTTGAGGAGAAGCTGAGCCTGGTTCCTGAGAACACTAAGCTATATTCAATATCTCCTGAGGCATATTCAGATGATAAGTTTGATAAGGCCAATTGCTACAAGCTAGAGAAGAGGTCTGAATATGAGCTAACTAGGCTCTACAAAGGACTAGTCCGGGACTTAGGGAATTCTTCCCCCTCCACTTATGCAGTCGAGAGATTGCTAGTCTTGGCAAGCCATCTGTATGAGACCAAGAAAGGGTCTTCTAACTTCTTCTTGACCGATTACCTGCCCAAGACAACATCAACAGCAAATCTTGATGCAGGCTTTTTGGCAAAGCTGAAAGAGACCCCTAAGGCCTCTGACCCCGACGTTTCCGATGTGACAGAAGTCAAGACAGCTAAGGCTACTCTAGACTCTGCTACAACTGATGCGGATACTAAAAAGGCCGCTTATGAGGCTATCGGAGATGAGGACTCGAAGAAGGCTGAGAAGGCTACAGCAAACACTGCCTGGATTGCTGCCCAAGAGGCCCAGAAGAAGGCTCAGAGTGCCTATGATAAGGCAGTTTCGAATGCGAAGAAGGCCTCAAGGAAAACTACCTCTGGAAAAAGCTTGTTCGGGGATGCAGGGGAGACAGTCACAGATGAGTCTAAGGTAGTGGAAGAGGTTGGAGAGGGGAAGAAGAAGTTTGGACCCTTCTTAGCTGCTTACTTGATGAGGCTGCTGACTAAAATAGCATCCAATGTCACAGAGTCATGGGAGCATATGAAAGGAATGTATAAGAACTTTTATGGTTATGATGCTCCTTCAGACCTGAACTGTCCCGAGGCAGGATTCCTTGAGCAACTGAAGTCTGAGCTTAACAAGGATAGAAGGGCGGCTACCTCATGGGTCAAAATAGTAGCAGAGGCAGATAATAAGTTGGATCAATCTACAGCCGAGGCTGGGATTCTCCGCTATGTGGCTGTCCTCCCTCTTGCATATTCTGGGATGCATGCGATGAAGTTGTTCATGGATGTCAAGATGCTGACCAAACTCACCAGCAACTACTTGATTGGTGCCATGAGGAGCCCTCTGACCAAGGATGCATTAGATGCAATCATGGATATCTTGATCTCGTTTGAGTCCACTACAAAGACTAAGAAGTCGGAGAAGTTCCGGTTTGCTAGGATTGTGAGTACTCAGTTCTTTCAATCCCTCCAGACCAAGAATTGCAAAGAGCTGGTCTATCTGATGGTTCAGATAATTGCTGAGTACAGGAAAGCAGAGGGGGTCAGGGATCCCATGAACATTGCTGGATTAGATGACATCTCTAGCAGGAACAAGAAGAAGTTGAATAAAGCAGTACGGATAATACTGGCAGAAGCTCCAAAGGCATCTGCAGGTGAGTATTCGTCAGCCATGAAGAAGGCTTTCCTGGATGACGAAGAGGATGATACAGCCAAGACCAGGTCCATCTTCCAGACCAAGGCTTAAGGAACATCATGTTTGGATAGAATATGACTCCTCTAAATAACTATGCAGCCTACTGGCGAGTCTTTGTGTGTTATATAAGAAAAACGAGACCGTCAGGTCAATAATGAGTGTGCCAGAGGATACTCCCTTCAGATCGTACTCCAGCATCTTTGACGACTCAGACTTTGTCCAACCACAGCCCATGTCCTTCAAAGCGACCAAGGAGTCAGAGAGCCTTCCTGAGACAGAGAAAGAAGATATGTCGACTGAATACCTCTCTGAACCACTGAGGACCAAGTCAGGTAAGAAGAACAGACGGAGGAAGGGAAAAGACCTAAAATCTCTCTTCACTCAAGAAGCTGGACTCCCGGCTCCAGAAGCAGACAGTGTCCTCCCCGAATCGTCTCCGTATGAGAATGATAACGCACAGTTGGAGCTACCCAAGCCCATTCTAAAGACATCAGACGCTCCGGTCTTCCTAAGAGAGAAGGATCTTAGTAAAGAATTTGCTGCAGCCTGCAAGACTAATGGGATTCTCCCAAGGGATGAATGGAAGTCATCTGTAGCAGCCAAGTATCATGCAGAGGAAGGGAAAATGACCAAACGAGACATCTCGTTAATCGTATTTGGAATGGAGCTTTACAAGAGATACAATGTGGAATCTGAGGTATCAACTTTGTTCACTTCATTGGTGACTGAGTTGCAGGGGATAAAGGTTGCTGCTAAGGAGTTGAATGATACCCGGGAGGTCCTCACTAAGATTCCAGGAGAAATTGTGTCTGCTGTCAAGGCAGGGGTAAAGGAGGGGACCGAGATGGGGATGGATTATATAGAGACTAGAACTAAAGTGGCCCCCAAAAGTGCTCCTAAAGTGGACATCTCCAAGCCGATGAGTAGTAAGATGATGGAGCAGCAGGATGAGAGTTCTGATGAGTCCTCAGATAATGAGAGTGAGGAGAGTGAGGAAGAATCCTTTGAGACAAAGGCTGCAATCTTCTTAGCTTTGATAAAGGTTCCAGAGGAAGAGAGAGACAACGCAATAGTCCTTATGGCCTTGAGGGCAGTGATATCTGACAGTGAGCTGAATCAGGCAATTAGGAATGACAGAATCTCCTCCTCAGTAGCAGATATGTACCATCAAAAGATATCTGACAAGGCTAGGGAATTGATGGGAAAGGGAAAGACCAACAAGAGGGCCAAGCAGCCTAAATCCTCTAAGTATGCATCGGATTACTATGATGATGCACTGTGAGTAATCAGTTACTACCTGTGTGGCAATGTCGGAACTGTACTTACCTATATATTGATAATCGTCTGTGATCTTGTGCCTTTAATTGCTGCAGTATTATAGCTTAAAATAATCTAGTTACCAATGCTCTATTTTCGTACTTAAATGCCATGTTGCCTGCCTTTACCAAAGTAATCTAGTGTGCTCTATTTAAGAAAAACGAGACCTTCATCATGAAGATCATCTGCAGTACTGGGATCTTCAATGAAGAGAAAGGCTTCCCCCTCCCCAACCTCCTCAACAGTCCCTTGATGAAGCAGGAGATCATGACGGTCAAGTACTTGAGGTTTCAGTATATCCCTATTATGACCAGCAACCCCTCGAGTTCATTGACTATTGACATTAAAGATACTCGACTGGTCAATTGGGATAATAGGTCGATTTTCCAGGTCAAAATATTCGGGGATGTTCAAAGCTCATTTATTGTATCGGGTCTACAGCCTTATTCAGCTAGAGACCGTTGCCCTTATCTCCTCTCCCTTTCAGTAAACGCAGGGAAGGTTGTTCCAGGGACCAAATACGGCATCTTAAAATCTTATGCTGTGTACACATCTAAAGACTCAGGGATAATTTCGTCACAGATCTCTGTAAAACTTGAAAGGTCCCCACGTGACTACTTCTTGAAGAGGTCTAAGGAGCACGACAAAAAAGATCTTGACAGTGATGTCTCATTCAAGATGTGTCGCCATGTCAAGTTTGCTACTTGAAGCTTGGAGAGGATGGGCAGCTGAATGTTGTCGGGGTGATGTTTTCCTTATGAATATCGTCAATAATTTCCCTGGAGGGAGAAACAGTTGATCTCTGGGAGGACGCAAGGGCTCACTACTAGCATGTGCTTGCAAGGTTGAGGTTTTATGCCTATCATGGTTTAGCCTAAATAAGAAAAACGAGACCATCATGGCCGTTCCGTGGACTGAGGTTAAAGACTCCAAGTACTTGGCAACTAAGATGTCTGTTACCCTGATCATGGAGATGAATGAAGATACACCTCTAAAGTATCCTTCTTACAACGCATTCGAGAGCATCTTTAAAAGGCTAGCAGAACCAGAAAGCGCAGCACCCCAAGTGGCCGCATGGTTTACTTGGTTCCTAAGAGAGGCAAAGGACATCTACTATCTGGAAGTGAGTAACAAGGAAACTGCCCAATACGGACCAACTAAAGTCTATAAACTTCAGTGTCCAGCCTACCTCTTATCCCGAGTGACAGGAGGATCACAGCTTGATTATACCTCTCTTATAGGTAGCAAGGTGATGACTGAGAAAGATCGGGGGATCCCAGTCAGAACACTCTATATTACTGGAGGGGGTACATCATTCAGAGTCATCAACGAGGAGACAGCCAACCAGTTCATCATAAATGATAATGCTGTCCGCCTACCAGGAGAGTGCAAGGTGGATGGAGGAAGTATAATCTGGAGTTAGAATTTCAAGAAAACTAAACAATAATGAACAAGTGGAACAATCTCGTGTAGTGTATTAGGTATCCTCTTTAACTAAAGTTAAATAAGAAAAACGAGACCCGCAAGGATCTTAAGATAACAAGATGATGAGGATTTCGGTCTTTCTCTTGATGCTCTGCTGGCTCCCTGTCAGCCTGACCTTCTTCGACAAATCACACATTCCTATAACCACATGTGATAAGAATCTAATGAGTCCTATCCCTTGGAGAACTTACTGCATAGAGGAGTGTGGAATCCGGAATGTAATAGGTGATAAGCTGGATCTGTTTATCTACAATAGGTCTGACAGTGGGAAAGTACAGCTGGCTGACTGCAGGAAGTACAAGATCAGACAGACCTTCACCAAGACTTGGACATTCTCGACATTCAAAGGGGCGATAGAGACAGAGGAGCTAATGCCTAATTATGCAGAGTGTGAATCCACCTGGAGAGATCTGTGCAACTCCGGACCGTGTAGTACCACAACTCCGGTGATCCCTGAGGAATACCATTGGGCCTCTGACACCACGAAGGAGGTCATCTATGTATCTATAGATGCATACCAGAAAACTGTTGCATTCCAGGATCCTAGTGGTGATATCCAGCTCCTAGTCCATGGGGTGATCATAGATGGGAGCCAGTCTGGTTATGTTCAACCCAGCAAAGATCTCATCACTATGTGGGATAAGGTTGAGTTACAAGATGAATGCCCTTGGTCAACGGGAAATTCTCTTTCGTGTTCTACATCTGATGAGGGAATTTCATACTACTGTGCTGGGAAAGGGCTAGTACTGACCAACATCAGTACGGTGACTGATACCAGATGTGACAACAACCCGCACCTAATGACATCAGGGCACCATGTGATTTTTAGAGTGAAGAAGGCATCAGACCCGAATGCGACTCTCAGCAGGACAGCTCAAATAGTGCTGGACAGGGGGTCAGAAGAGGCCGAGATTGTAGATAGTGTTAATAAGGCGTTGCTGGATAGAGATTCCATCAGGTGTGCAAGCTCATGTCTCGCCTTTGATTACACCATCTCCAAGCCTCAGATGTTTGGCAACCAATTGGCGCTACCTTATAAGGGGTCTTTTCTCCCTTGCAACATACTGCCTAATTGTCGGGTTGTCTTCCCAGTCAAGTATTGCAGCTCTCCTCCGATGATTCTGGTAGAATGTACCGGCACTATGACATGGTGGAATATCACTGGAGATTACACGATCAGACCCACCTATTGCCACATGAACCAGTCGGCGACCAAGATTAAGACATCTATATCCTTTATGACAACAAATGGGAGAGTCTTAGTGAATGAGTCTGGCGCTTATCCTGTCTCCCGTGAAATAGGAAATACGTTCCAGGTCGGACATGTCATAGAGCCTAGCTCCATGATAGAGGTGACTGATCCACTGAATGTTAGGATAGATGACACCTTAGTCACACCAGAGTCCCATACTATATCTAATATCACTTCAGTCGGGGACTCACTTTTGGATACAATGGTTGAGACCGTGAAAGGTATCGGTCGCTTCATATCCCATGAGGTCAGGATAGTGGTTTTTGGTGTTCTGACTCTTTTTATATTATATCTGTCGTTTAAATATTTGTTCGCTAAGAAGAAGAGCAGAGTGCCGCACCCTAAAGTTGTCTATACAAAACCCACATCTGAAGGACCAGTGATCTATGACACTGAATATACTATAGAAAGTGACTAATAAAAAACAGAGACCAACATGGAGTTCAACTGGCCTTGGGGACAGAACAGTGAAACGGAAATCACCAAGAATCTCCGCTTTGAGGACATTAAGGTGATGGCCATAATAATACTAGTCTGGGTGAAGTGTCTTCTCATCTACCATTTCAAGAGGAAAATAAGGCGACTAAGATCTCTATTGATAAAAGGATCCTCACAATGGGTACTGCATGATGCCTAACTCATTAGGAAGATCTGAGATGAGTTTTTATCCTGCCTTATCCTTCTAATTAAGAAAAACGAGACCGCCATCATGGACCTTGATGACGGTGGTCTATGGAGACGTGCTAGGGGTCTGGGGGATTATCACCTGAGGTCTGCTCTGGTGACCCCCTCCTTAGAGCGTTTCCGCAGTCGAAAGGGAAGGCACCGTGAACAACTGTGCTTTGATAGGATGAAATCACTAGGCTGGATGTTGAGGTGGGTAGATCAAGGGAAACTGCTTGGATATTTAATGGTAGAAGCCAACAAATCTTTACCAAAATCCATTGCAAACCAGGAACTCCTAGTTGAGACTCTGAAGTTGGAATACGGATGTCTTAGACAGATAATCATGACGGATGGAGACCTCCATGATCAGGTGATCTCTTATCTAGACAGGAAGTCTATCTCTACCCATTACACCCATGGTAGGGAGGTCTTTCAGGAAGCCTTGATAGTTGTCATGGCACTTTCTTCAGGGAGAGAACCACCAGATCATGTCAATAACTTGGGTTATGAAATGCTGAATGAAGAACTTGAAGTGCCCGTGGTCAGAACCTATGGCGTAATCTTCTATCTCTTTGGGGACTTGATCTACGTAAAGTATCCGGAGGAAGAGGGCATGATCACACTGGACATGTTCAGAAACCTGACAGATAAATTCTCTGAGAGGGAGAATATTATGATTGCAACTCAACTAGGAACTGAGATCCTTCAAGAGATATACCCTTCTGAGACAGTGCTCAAAACAGTCTTTTCTCTTTGGGACAAAGGTCTATTGAAAGAGGGAAATGACTTTTACACAGTGGTAAAGACATTCGAAGCAATCATAAATGGGATGTTGATCAAGAACAATGACGGAACATACTATGATCCTTCAGCATACCTCCGAGAAACTATCATGGGGCTGCCAGTACGACTGAGGGATTATGCACAGACGTTAGTGAGTTACTTAGACTCTCTCCCCTTTAATCCACACCACCTATCTCAGATTGGGGGGTTGTTTAGGTTGTGGGGACACCCCATAGTAGATCCTAACGCTGGGGTCAGAAAGGTAAGGCTTCTGGGAACAGCTGATAAGATGAACCTGACACATATCCCCACCCTAGCAGAGCGGAAATTCAAGGAGATCTTCTACCTGTCGTATTATGAAAAGCATAGAGTCTATCCTAATCATAACTTGAATGGAGAAATTGAAGGAAGCTATTTGCTCTCTCAGCTTGCACAAATGGCTCTGGTTAATCCCAAGCATGCAAATTACTCACTAGTAGACTGGGATTCAGTCAACACACTAGAGACCTTTCCCTTTCCTAAATCATTCAACCTGTCTCTTATTATAGCGGATAAAGCTGTAAGTCCGAACAGAGAAGAATGGTTGGAATTGAGACGTAAGGGAGGAACTCAGATGGATCCGCACATTAGAAGAGGGCCTCTGAAGGCTATGAAAGACGGAGTAATCGATTGTGAGAAACTATTGAGGAAGATAGACCGTAACCCGTCCGGTTTAGCTAAGAAACATAGGATCATTGGACTGTACCCAAAGGAGAGAGAAGAAAATATGGTTCCCAGGATGTTTGCATTAATGTCCTTTGACATGAGAGCTTTCTCAGTGGTCTCTGAATCCATGATAGCAGATCACATCATTCCTCATATAGAAGGTGTCACAATGACGAAGAGCATGTTGGCCCTCCAGAAAGAGATGATCATTTCAACTAAAAGCCAGGCCTCCTCAACCCAGTCAGACAGTATAACCTTCTGTCTTAATATTGACTTTGAAAAGTGGAACCTTAACTTCCGAAGATGGATGACGGAGGGGGTATTCAGAGAAATGGGGCGATTGTTCGGACTTCCAGAGATATTCAATCGGACTTATGACATCTTCAAGAAATCCATCATTTATCTAGCAGATGGAAGCTTCGACCTCTTGCTGACAGATGAGTTGGAAATAGAACCAGGGACCAATCCAGATTGCGCATACACCGGTCATGTTGGGGGGTTTGAGGGATTGAGGCAGAAAGGATGGACAGTCTTCACAGCGGTCTTAATCTCTTCCATCTGCGATGAAATGGGGATCAAGACTCATCTAATGGGGCAAGGTGATAACCAGGTTTTGATGCTGACCATCTACTCAAGAGCTGCTAGAGAGACGGGGGATTTGAAATCAGCACCAGCAGTACTGGAGATCACAAATACATTGGAGACATTCAAGAGCAGACTAGTCTCACTCTTTGCTAATCTTGGATTGCCTATAAAACCACTAGAGACCTGGGTCTCAGAGGAATTGTTTGCCTATGGCAAGACTCCAATATACCGAGCAGTCCCCCTTGCAATGAGTCTCAAGCGAATCTCTAGAGTTTTTGCGTTCTCCAATGAGGATCTAATGACCTTGTCTAATGCGTTAGGTGCTATCTCTGCGAACGCACAAGCTGCATCCATGTGCGATGTCCATCCGATGGTGTCATATGCCATAGCAAAGTGGCAACACTTATGCTGTGCGATAATCTTCTCCAACTATCATCCGCTATGCGGGTGTGCTCCACATGTCTCAGGAGAGGAATGGGCAATAAAGCTACGACTTCCGTCAGGAAAGAAGATTCAAGAGACATCTGATGAGGAGATAGACGAAAGAGATCTGATGAAACTAATAGTGACCATACCTCGAAGCTTAGGAGGTTATAATACCCTGACTCTGTATGAGATGATCATGAGAGGATTTTCTGACCCAGTCTCTAGGGACATGTGCTGGCTATTCGCAATTGCCAGTGAATCAACAGGAAAACTTCGAGGTTATCTGATAAACTGGATTAAACCAATCGTGTCTCCGGAAGTAAATGCTCAGCATCTCATTCAGGATCCCACGGCACTCAATTTGCTAGTACCCCCTAATGCTACATCAGTTATTAAGAGGATGATAGACAAATCACTCGAAGCACTCCCAAAGAGATCTCGGTTCGCAGTATGGTTCTCTGAGATCCTAGAGATCTCAGGGGACAAGGAAATCTCAAAGTTAGCAGAGGCCTTGACCAGGACAGATTCCCTGAACCCCCGTTTCCTTCACGACATTCTGGGGGCCACTCTTTATGGATACTGCACGGCTATAACTAGTAAAGTTGACAAGACAGTGACCTTATCTCGGATGGCACTAGCGTCTAAGGATGTTGTTGGAGCTCTAATCAAGGGGGAGATGAGACTTTACTCCTATTTCGGATGGAGAACCTTGCAATCGAGAGGCTTACCTCTGACCACCAGATGTCCCAATAAGTGGGTGCGGATAATTAGGGATATCTCATGGCAAAAACAAATTAAGGCTGTTAGCGTTCCGTATCCTACTCATTTCTTATCAGAGGATATTTCAGAGACTGATAGGCCCGATTCCTGGATTGAGTGCTATATTGATGATGCCCCGACATCTGACAGAAGTTGCATGATCTATTCGACAGGAAAAGCTCTGCCGTACTTGGGTAGTGTCACTAGAGAAAAACTGACAACTCGAGGTGCAAAGGCGGCCTATGGAACTGAACCTCTTGTATTGAGGCCAATTAATCTAGTCAGAACTATAGGTTGGTTCATTGAAGAGGATAGCAATTTTGCTGAATTGATCAAGATGCTACTGGGAGCCGTCACAGATCTTCCCATAGAGGAGGTACTCTATATCCCAGAAATGGTGAGTGGCTCTATGGCACATAGATACCTAGATATGTCAACACAGCATGGATCTTTGTGGATGCCTCTTTATGGTCCTGCCACTTTTCTCCATATGAGCACTAATACTTTTGTGCAGTACCTGAAGGGGACAGAAAATGTCACTTTGCACTTTCAGTGTGTCATGGGCTTAATTCAGTATGCTATAGTCAACAAAGCTCTAGGGGAATGTCCCACAAAGAGAATGACGAGATTCTTCCGATCATGCCCTGACTGTATAGTTCCCATTGATGATACGTTGGAGGACTTGCCTGAGGTCCCTTCGCTAGATCTCATCCCTGAGAGGACCACAAACCCTTATCTCTACCTCAAGAAGGAGAAGATTGAACTGAATGTTAGGCATCGACTTGCAGTGATAAATGAGATCCGGGTAATAGCTAGGGATGAGATAGAAGAGACTCCCCTCTTGGCTAACAATACTCTGGAGGACGTGATGAGTTTGAGGGCAGCTCAGAGAATATTCTACACAGCCAAAGGAAAGGAGGCACAGTGGGACTTGCAGACTGCTGATCGAGAGGGGTACTTAAAACTGGATTTCGTGAGCGTAATCAGGAAGATTATAGGACACCTCTTTGTAATGGAATCAGAGACGTTAAAAGCCGGGTCAGACTACCCTACTTTCCAGTTACAGCAGAGAAGGATCATGCGGCGCGTTAGGCAGTCAGATACATCTAACTTTGTCCACTTAGGAGGGTTCTTCTGCTGGCAAGAGAGCATTGCGAGGATACAGAAACTGAAGTGGTCAGTGATGCCTGCCACATTCCCTATCACTGCAGAATCAGTGTCACTGGCAGCTAAGATGAGTCTAATAGGGGCAATGACTAGCGGGCTAACACCTAAGAGATGTGATGGTGTTCTGGCCGAAAACTTAATCCCGGATGTCACAAGGCAAGCAAAGAACATCATATGCCTGGACAAAGTGTTCCAAAGCAGATGTGATTACTGCTATACTGCAGCAATGACAAATAGGTGGTCAGACTCGATAAACTCCGACAGCATATTCAATATGAGATGTGAGAGAGGCCATACCATTCTGTCCCCGAGAATGTTGCATCAATTGAGGCGAACTATCTTGCCTGAAGGAGCCCTATACACTTTGGCTGTCAGAGTGGTTCACCAAAGCCCTCCTGAAGCATCACAACCTCTTCAGATCATCCCATGCAGGCGAGAGAGATATCAAATCTTGAGTGAAGCTGACATGCCTAGACAAACTGGCTACTCGGGGGTCTATAGAAGGCCTACCAATCAAATCTGCATGTATACAGAGGTTGAATTGGCAAAGAAGTACAGGTTGCCAACTAATTCATTATACAGGATCCTTGACTTACACGACTGCTTCTTCAGTCAGAAACTCATGGACACAAGGGGCAACATTTTAGTGGTCGGAGACGGGTATGGATACAGCTCCTTGCTAACCAAGTGCTTGAACCCAGACAGGAATGTAGTGAGCTGGACCTATATTGAACCATCTGAGGCACTTCCACATAGCCTGAGAATCTCTAAGCCTCCAATGCACTACAAAGCTGATGTGCAGATAGATTCTAGCCCTTCAATTGACAGAATCTCCGACATACATAACTCCTCATACCCGGATGAGTTCGCTAAAGTGGTGACCAAGAATGGGGTTACAGCACTGATCAGTGACATTGAAACTGTGTATGTCTCTGGAGAGAAGAGTGTGGCGTCTTTGATCAACTTGGCTTGGAATAACCAGATCCAATTAGGAGCACTCAAGTTGGAATTGATGACGGATCCCTTGGAGAGAGTCGTAGAATATGCCCACAATGCTTACCAAAGATGGGAATTATTCACTCTACCAGGCGCCAATCTCGGAGGAGGGGTACTATACATCGGTTTCTACGGTAGAAGAGAGAAGTTGACTGGGTACATCATTCCCCATTCAGGAGTTGAGACCTTGATGGATAGGTTGGCAACTGAGGTAGATGAGGATAGAGGAAGATTGAGAGCAGAGGACCTTGATCGGTGGGAGCAGTTGGATACAATGGAAAGCAAAATACATCTGCAGCAATACCATAAGATGAGGCTCGATCTATGGTTCGGATCTGCTTATCTGAGTGGTTATCTCTCAGAGGATATGACTGAGTTCTTTTACTCAGTAAAAACATCTTACAGACCACCTGCAATCTCATGGGAGAAAGGGAACCAGGCCAGATACCTCTATGGCAGCAGGGAAGAGATCTTATTCATGAGCATGATCACAGTGGCACTATCATCATATCTAGAGGACCAGGATGTCTTAAGAGAGTTCCTTTCTTCCTCAGGGTGGAAACTCCAGTGGAATAAGGATAAGAAGAATCCGAGGCATTGGTCCCCGTATTTGGAGCGGAGTGACTCTCAACTAGTCTGGAAAAAGAACAAAGCCATGATTTTCAAACTGGTATCAGTTCTGAGAGGATCAAAGCCGAGAGAAGAATATAAGATGGGAAAAGCATCTGACTCCATAGTTTTCAGATACATTCCGAGGAAGGTAAGGGAAGGAGAAGTTCCCCTGTGCTTCCCGATCTCTAAGGTGGCCTCAATGTCGGTATAAGAAAAACGAGACCTTCATCATGGATGATTTAATGGTCTATAGCCTTTGGGCTCTGCTGCAATGGGGTTTGCCTGCGGATACAGCCAGATACATAATCTCACTGAGGATGGAAGAAGACTACTGGGAAGAGATGAATGAACATGAGCAGTGGGACGGGTACGAGTCTTGGGATGATACTGATTGGCAGTATCCGAGTCCTTGAATAAGTCAGTATGTGACAATTTAGAAAAAACGATTACCTTACTGTTGACTTTAAAGAACACTGTTATGTTGTGTTCGCAACACACAACTTCCTT

>Seq9 Rice stripe mosaic virus HZ isolate 5, complete genome

AAGGAAGTTGCGTTGCGAACGCAACATTAAAATGTTGACATAACCCACCTTATCATAACAAAAGAAAAACGAGACCGCCAAGTCTTAAAATGGCAACCGACAAGTCTTTTGAGGAGAAGCTGAGCCTGGTTCCTGAGAACACTAAGCTATATTCAATATCTCCTGAGGCATATTCAGATGATAAGTTTGATAAGGCCAATTGCTACAAGCTAGAGAAGAGGTCTGAATATGAGCTAACTAGGCTCTACAAAGGACTAGTCCGGGACTTAGGGAATTCTTCCCCCTCCACTTATGCAGTCGAGAGATTGCTAGTCTTGGCAAGCCATCTGTATGAGACCAAGAAAGGGTCTTCTAACTTCTTCTTGACCGATTACCTGCCCAAGACAACATCAACAGCAAATCTTGATGCAGGCTTTTTGGCAAAGCTGAAAGAGACCCCTAAGGCCTCTGACCCCGACGTTTCCGATGTGACAGAAGTCAAGACAGCTAAGGCTACTCTAGACTCTGCTACAACTGATGCGGATACTAAAAAGGCCGCTTATGAGGCTATCGGAGATGAGGACTCGAAGAAGGCTGAGAAGGCTACAGCAAACACTGCCTGGATTGCTGCCCAAGAGGCCCAGAAGAAGGCTCAGAGTGCCTATGATAAGGCAGTTTCGAATGCGAAGAAGGCCTCAAGGAAAACTACCTCTGGAAAAAGCTTGTTCGGGGATGCAGGGGAGACAGTCACAGATGAGTCTAAGGTAGTGGAAGAGGTTGGAGAGGGGAAGAAGAAGTTTGGACCCTTCTTAGCTGCTTACTTGATGAGGCTGCTGACTAAAATAGCATCCAATGTCACAGAGTCATGGGAGCATATGAAAGGAATGTATAAGAACTTTTATGGTTATGATGCTCCTTCAGACCTGAACTGTCCCGAGGCAGGATTCCTTGAGCAACTGAAGTCTGAGCTTAACAAGGATAGAAGGGCGGCTACCTCATGGGTCAAAATAGTAGCAGAGGCAGATAATAAGTTGGATCAATCTACAGCCGAGGCTGGGATTCTCCGCTATGTGGCTGTCCTCCCTCTTGCATATTCTGGGATGCATGCGATGAAGTTGTTCATGGATGTCAAGATGCTGACCAAACTCACCAGCAACTACTTGATTGGTGCCATGAGGAGCCCTCTGACCAAGGATGCATTAGATGCAATCATGGATATCTTGATCTCGTTTGAGTCCACTACAAAGACTAAGAAGTCGGAGAAGTTCCGGTTTGCTAGGATTGTGAGTACTCAGTTCTTTCAATCCCTCCAGACCAAGAATTGCAAAGAGCTGGTCTATCTGATGGTTCAGATAATTGCTGAGTACAGGAAAGCAGAGGGGGTCAGGGATCCCATGAACATTGCTGGATTAGATGACATCTCTAGCAGGAACAAGAAGAAGTTGAATAAAGCAGTACGGATAATACTGGCAGAAGCTCCAAAGGCATCTGCAGGTGAGTATTCGTCAGCCATGAAGAAGGCTTTCCTGGATGACGAAGAGGATGATACAGCCAAGACCAGGTCCATCTTCCAGACCAAGGCTTAAGGAACATCATGTTTGGATAGAATATGACTCCTCTAAATAACTATGCAGCCTACTGGCGAGTCTTTGTGTGTTATATAAGAAAAACGAGACCGTCAGGTCAATAATGAGTGTGCCAGAGGATACTCCCTTCAGATCGTACTCCAGCATCTTTGACGACTCAGACTTTGTCCAACCACAGCCCATGTCCTTCAAAGCGACCAAGGAGTCAGAGAGCCTTCCTGAGACAGAGAAAGAAGATATGTCGACTGAATACCTCTCTGAACCACTGAGGACCAAGTCAGGTAAGAAGAACAGACGGAGGAAGGGAAAAGACCTAAAATCTCTCTTCACTCAAGAAGCTGGACTCCCGGCTCCAGAAGCAGACAGTGTCCTCCCCGAATCGTCTCCGTATGAGAATGATAACGCACAGTTGGAGCTACCCAAGCCCATTCTAAAGACATCAGACGCTCCGGTCTTCCTAAGAGAGAAGGATCTTAGTAAAGAATTTGCTGCAGCCTGCAAGACTAATGGGATTCTCCCAAGGGATGAATGGAAGTCATCTGTAGCAGCCAAGTATCATGCAGAGGAAGGGAAAATGACCAAACGAGACATCTCGTTAATCGTATTTGGAATGGAGCTTTACAAGAGATACAATGTGGAATCTGAGGTATCAACTTTGTTCACTTCATTGGTGACTGAGTTGCAGGGGATAAAGGTTGCTGCTAAGGAGTTGAATGATACCCGGGAGGTCCTCACTAAGATTCCAGGAGAAATTGTGTCTGCTGTCAAGGCAGGGGTAAAGGAGGGGACCGAGATGGGGATGGATTATATAGAGACTAGAACTAAAGTGGCCCCCAAAAGTGCTCCTAAAGTGGACATCTCCAAGCCGATGAGTAGTAAGATGATGGAGCAGCAGGATGAGAGTTCTGATGAGTCCTCAGATAATGAGAGTGAGGAGAGTGAGGAAGAATCCTTTGAGACAAAGGCTGCAATCTTCTTAGCTTTGATAAAGGTTCCAGAGGAAGAGAGAGACAACGCAATAGTCCTTATGGCCTTGAGGGCAGTGATATCTGACAGTGAGCTGAATCAGGCAATTAGGAATGACAGAATCTCCTCCTCAGTAGCAGATATGTACCATCAAAAGATATCTGACAAGGCTAGGGAATTGATGGGAAAGGGAAAGACCAACAAGAGGGCCAAGCAGCCTAAATCCTCTAAGTATGCATCGGATTACTATGATGATGCACTGTGAGTAATCAGTTACTACCTGTGTGGCAATGTCGGAACTGTACTTACCTATATATTGATAATCGTCTGTGATCTTGTGCCTTTAATTGCTGCAGTATTATAGCTTAAAATAATCTAGTTACCAATGCTCTATTTTCGTACTTAAATGCCATGTTGCCTGCCTTTACCAAAGTAATCTAGTGTGCTCTATTTAAGAAAAACGAGACCTTCATCATGAAGATCATCTGCAGTACTGGGATCTTCAATGAAGAGAAAGGCTTCCCCCTCCCCAACCTCCTCAACAGTCCCTTGATGAAGCAGGAGATCATGACGGTCAAGTACTTGAGGTTTCAGTATATCCCTATTATGACCAGCAACCCCTCGAGTTCATTGACTATTGACATTAAAGATACTCGACTGGTCAATTGGGATAATAGGTCGATTTTCCAGGTCAAAATATTCGGGGATGTTCAAAGCTCATTTATTGTATCGGGTCTACAGCCTTATTCAGCTAGAGACCGTTGCCCTTATCTCCTCTCCCTTTCAGTAAACGCAGGGAAGGTTGTTCCAGGGACCAAATACGGCATCTTAAAATCTTATGCTGTGTACACATCTAAAGACTCAGGGATAATTTCGTCACAGATCTCTGTAAAACTTGAAAGGTCCCCACGTGACTACTTCTTGAAGAGGTCTAAGGAGCACGACAAAAAAGATCTTGACAGTGATGTCTCATTCAAGATGTGTCGCCATGTCAAGTTTGCTACTTGAAGCTTGGAGAGGATGGGCAGCTGAATGTTGTCGGGGTGATGTTTTCCTTATGAATATCGTCAATAATTTCCCTGGAGGGAGAAACAGTTGATCTCTGGGAGGACGCAAGGGCTCACTACTAGCATGTGCTTGCAAGGTTGAGGTTTTATGCCTATCATGGTTTAGCCTAAATAAGAAAAACGAGACCATCATGGCCGTTCCGTGGACTGAGGTTAAAGACTCCAAGTACTTGGCAACTAAGATGTCTGTTACCCTGATCATGGAGATGAATGAAGATACACCTCTAAAGTATCCTTCTTACAACGCATTCGAGAGCATCTTTAAAAGGCTAGCAGAACCAGAAAGCGCAGCACCCCAAGTGGCCGCATGGTTTACTTGGTTCCTAAGAGAGGCAAAGGACATCTACTATCTGGAAGTGAGTAACAAGGAAACTGCCCAATACGGACCAACTAAAGTCTATAAACTTCAGTGTCCAGCCTACCTCTTATCCCGAGTGACAGGAGGATCACAGCTTGATTATACCTCTCTTATAGGTAGCAAGGTGATGACTGAGAAAGATCGGGGGATCCCAGTCAGAACACTCTATATTACTGGAGGGGGTACATCATTCAGAGTCATCAACGAGGAGACAGCCAACCAGTTCATCATAAATGATAATGCTGTCCGCCTACCAGGAGAGTGCAAGGTGGATGGAGGAAGTATAATCTGGAGTTAGAATTTCAAGAAAACTAAACAATAATGAACAAGTGGAACAATCTCGTGTAGTGTATTAGGTATCCTCTTTAACTAAAGTTAAATAAGAAAAACGAGACCCGCAAGGATCTTAAGATAACAAGATGATGAGGATTTCGGTCTTTCTCTTGATGCTCTGCTGGCTCCCTGTCAGCCTGACCTTCTTCGACAAATCACACATTCCTATAACCACATGTGATAAGAATCTAATGAGTCCTATCCCTTGGAGAACTTACTGCATAGAGGAGTGTGGAATCCGGAATGTAATAGGTGATAAGCTGGATCTGTTTATCTACAATAGGTCTGACAGTGGGAAAGTACAGCTGGCTGACTGCAGGAAGTACAAGATCAGACAGACCTTCACCAAGACTTGGACATTCTCGACATTCAAAGGGGCGATAGAGACAGAGGAGCTAATGCCTAATTATGCAGAGTGTGAATCCACCTGGAGAGATCTGTGCAACTCCGGACCGTGTAGTACCACAACTCCGGTGATCCCTGAGGAATACCATTGGGCCTCTGACACCACGAAGGAGGTCATCTATGTATCTATAGATGCATACCAGAAAACTGTTGCATTCCAGGATCCTAGTGGTGATATCCAGCTCCTAGTCCATGGGGTGATCATAGATGGGAGCCAGTCTGGTTATGTTCAACCCAGCAAAGATCTCATCACTATGTGGGATAAGGTTGAGTTACAAGATGAATGCCCTTGGTCAACGGGAAATTCTCTTTCGTGTTCTACATCTGATGAGGGAATTTCATACTACTGTGCTGGGAAAGGGCTAGTACTGACCAACATCAGTACGGTGACTGATACCAGATGTGACAACAACCCGCACCTAATGACATCAGGGCACCATGTGATTTTTAGAGTGAAGAAGGCATCAGACCCGAATGCGACTCTCAGCAGGACAGCTCAAATAGTGCTGGACAGGGGGTCAGAAGAGGCCGAGATTGTAGATAGTGTTAATAAGGCGTTGCTGGATAGAGATTCCATCAGGTGTGCAAGCTCATGTCTCGCCTTTGATTACACCATCTCCAAGCCTCAGATGTTTGGCAACCAATTGGCGCTACCTTATAAGGGGTCTTTTCTCCCTTGCAACATACTGCCTAATTGTCGGGTTGTCTTCCCAGTCAAGTATTGCAGCTCTCCTCCGATGATTCTGGTAGAATGTACCGGCACTATGACATGGTGGAATATCACTGGAGATTACACGATCAGACCCACCTATTGCCACATGAACCAGTCGGCGACCAAGATTAAGACATCTATATCCTTTATGACAACAAATGGGAGAGTCTTAGTGAATGAGTCTGGCGCTTATCCTGTCTCCCGTGAAATAGGAAATACGTTCCAGGTCGGACATGTCATAGAGCCTAGCTCCATGATAGAGGTGACTGATCCACTGAATGTTAGGATAGATGACACCTTAGTCACACCAGAGTCCCATACTATATCTAATATCACTTCAGTCGGGGACTCACTTTTGGATACAATGGTTGAGACCGTGAAAGGTATCGGTCGCTTCATATCCCATGAGGTCAGGATAGTGGTTTTTGGTGTTCTGACTCTTTTTATATTATATCTGTCGTTTAAATATTTGTTCGCTAAGAAGAAGAGCAGAGTGCCGCACCCTAAAGTTGTCTATACAAAACCCACATCTGAAGGACCAGTGATCTATGACACTGAATATACTATAGAAAGTGACTAATAAAAAACAGAGACCAACATGGAGTTCAACTGGCCTTGGGGACAGAACAGTGAAACGGAAATCACCAAGAATCTCCGCTTTGAGGACATTAAGGTGATGGCCATAATAATACTAGTCTGGGTGAAGTGTCTTCTCATCTACCATTTCAAGAGGAAAATAAGGCGACTAAGATCTCTATTGATAAAAGGATCCTCACAATGGGTACTGCATGATGCCTAACTCATTAGGAAGATCTGAGATGAGTTTTTATCCTGCCTTATCCTTCTAATTAAGAAAAACGAGACCGCCATCATGGACCTTGATGACGGTGGTCTATGGAGACGTGCTAGGGGTCTGGGGGATTATCACCTGAGGTCTGCTCTGGTGACCCCCTCCTTAGAGCGTTTCCGCAGTCGAAAGGGAAGGCACCGTGAACAACTGTGCTTTGATAGGATGAAATCACTAGGCTGGATGTTGAGGTGGGTAGATCAAGGGAAACTGCTTGGATATTTAATGGTAGAAGCCAACAAATCTTTACCAAAATCCATTGCAAACCAGGAACTCCTAGTTGAGACTCTGAAGTTGGAATACGGATGTCTTAGACAGATAATCATGACGGATGGAGACCTCCATGATCAGGTGATCTCTTATCTAGACAGGAAGTCTATCTCTACCCATTACACCCATGGTAGGGAGGTCTTTCAGGAAGCCTTGATAGTTGTCATGGCACTTTCTTCAGGGAGAGAACCACCAGATCATGTCAATAACTTGGGTTATGAAATGCTGAATGAAGAACTTGAAGTGCCCGTGGTCAGAACCTATGGCGTAATCTTCTATCTCTTTGGGGACTTGATCTACGTAAAGTATCCGGAGGAAGAGGGCATGATCACACTGGACATGTTCAGAAACCTGACAGATAAATTCTCTGAGAGGGAGAATATTATGATTGCAACTCAACTAGGAACTGAGATCCTTCAAGAGATATACCCTTCTGAGACAGTGCTCAAAACAGTCTTTTCTCTTTGGGACAAAGGTCTATTGAAAGAGGGAAATGACTTTTACACAGTGGTAAAGACATTCGAAGCAATCATAAATGGGATGTTGATCAAGAACAATGACGGAACATACTATGATCCTTCAGCATACCTCCGAGAAACTATCATGGGGCTGCCAGTACGACTGAGGGATTATGCACAGACGTTAGTGAGTTACTTAGACTCTCTCCCCTTTAATCCACACCACCTATCTCAGATTGGGGGGTTGTTTAGGTTGTGGGGACACCCCATAGTAGATCCTAACGCTGGGGTCAGAAAGGTAAGGCTTCTGGGAACAGCTGATAAGATGAACCTGACACATATCCCCACCCTAGCAGAGCGGAAATTCAAGGAGATCTTCTACCTGTCGTATTATGAAAAGCATAGAGTCTATCCTAATCATAACTTGAATGGAGAAATTGAAGGAAGCTATTTGCTCTCTCAGCTTGCACAAATGGCTCTGGTTAATCCCAAGCATGCAAATTACTCACTAGTAGACTGGGATTCAGTCAACACACTAGAGACCTTTCCCTTTCCTAAATCATTCAACCTGTCTCTTATTATAGCGGATAAAGCTGTAAGTCCGAACAGAGAAGAATGGTTGGAATTGAGACGTAAGGGAGGAACTCAGATGGATCCGCACATTAGAAGAGGGCCTCTGAAGGCTATGAAAGACGGAGTAATCGATTGTGAGAAACTATTGAGGAAGATAGACCGTAACCCGTCCGGTTTAGCTAAGAAACATAGGATCATTGGACTGTACCCAAAGGAGAGAGAAGAAAATATGGTTCCCAGGATGTTTGCATTAATGTCCTTTGACATGAGAGCTTTCTCAGTGGTCTCTGAATCCATGATAGCAGATCACATCATTCCTCATATAGAAGGTGTCACAATGACGAAGAGCATGTTGGCCCTCCAGAAAGAGATGATCATTTCAACTAAAAGCCAGGCCTCCTCAACCCAGTCAGACAGTATAACCTTCTGTCTTAATATTGACTTTGAAAAGTGGAACCTTAACTTCCGAAGATGGATGACGGAGGGGGTATTCAGAGAAATGGGGCGATTGTTCGGACTTCCAGAGATATTCAATCGGACTTATGACATCTTCAAGAAATCCATCATTTATCTAGCAGATGGAAGCTTCGACCTCTTGCTGACAGATGAGTTGGAAATAGAACCAGGGACCAATCCAGATTGCGCATACACCGGTCATGTTGGGGGGTTTGAGGGATTGAGGCAGAAAGGATGGACAGTCTTCACAGCGGTCTTAATCTCTTCCATCTGCGATGAAATGGGGATCAAGACTCATCTAATGGGGCAAGGTGATAACCAGGTTTTGATGCTGACCATCTACTCAAGAGCTGCTAGAGAGACGGGGGATTTGAAATCAGCACCAGCAGTACTGGAGATCACAAATACATTGGAGACATTCAAGAGCAGACTAGTCTCACTCTTTGCTAATCTTGGATTGCCTATAAAACCACTAGAGACCTGGGTCTCAGAGGAATTGTTTGCCTATGGCAAGACTCCAATATACCGAGCAGTCCCCCTTGCAATGAGTCTCAAGCGAATCTCTAGAGTTTTTGCGTTCTCCAATGAGGATCTAATGACCTTGTCTAATGCGTTAGGTGCTATCTCTGCGAACGCACAAGCTGCATCCATGTGCGATGTCCATCCGATGGTGTCATATGCCATAGCAAAGTGGCAACACTTATGCTGTGCGATAATCTTCTCCAACTATCATCCGCTATGCGGGTGTGCTCCACATGTCTCAGGAGAGGAATGGGCAATAAAGCTACGACTTCCGTCAGGAAAGAAGATTCAAGAGACATCTGATGAGGAGATAGACGAAAGAGATCTGATGAAACTAATAGTGACCATACCTCGAAGCTTAGGAGGTTATAATACCCTGACTCTGTATGAGATGATCATGAGAGGATTTTCTGACCCAGTCTCTAGGGACATGTGCTGGCTATTCGCAATTGCCAGTGAATCAACAGGAAAACTTCGAGGTTATCTGATAAACTGGATTAAACCAATCGTGTCTCCGGAAGTAAATGCTCAGCATCTCATTCAGGATCCCACGGCACTCAATTTGCTAGTACCCCCTAATGCTACATCAGTTATTAAGAGGATGATAGACAAATCACTCGAAGCACTCCCAAAGAGATCTCGGTTCGCAGTATGGTTCTCTGAGATCCTAGAGATCTCAGGGGACAAGGAAATCTCAAAGTTAGCAGAGGCCTTGACCAGGACAGATTCCCTGAACCCCCGTTTCCTTCACGACATTCTGGGGGCCACTCTTTATGGATACTGCACGGCTATAACTAGTAAAGTTGACAAGACAGTGACCTTATCTCGGATGGCACTAGCGTCTAAGGATGTTGTTGGAGCTCTAATCAAGGGGGAGATGAGACTTTACTCCTATTTCGGATGGAGAACCTTGCAATCGAGAGGCTTACCTCTGACCACCAGATGTCCCAATAAGTGGGTGCGGATAATTAGGGATATCTCATGGCAAAAACAAATTAAGGCTGTTAGCGTTCCGTATCCTACTCATTTCTTATCAGAGGATATTTCAGAGACTGATAGGCCCGATTCCTGGATTGAGTGCTATATTGATGATGCCCCGACATCTGACAGAAGTTGCATGATCTATTCGACAGGAAAAGCTCTGCCGTACTTGGGTAGTGTCACTAGAGAAAAACTGACAACTCGAGGTGCAAAGGCGGCCTATGGAACTGAACCTCTTGTATTGAGGCCAATTAATCTAGTCAGAACTATAGGTTGGTTCATTGAAGAGGATAGCAATTTTGCTGAATTGATCAAGATGCTACTGGGAGCCGTCACAGATCTTCCCATAGAGGAGGTACTCTATATCCCAGAAATGGTGAGTGGCTCTATGGCACATAGATACCTAGATATGTCAACACAGCATGGATCTTTGTGGATGCCTCTTTATGGTCCTGCCACTTTTCTCCATATGAGCACTAATACTTTTGTGCAGTACCTGAAGGGGACAGAAAATGTCACTTTGCACTTTCAGTGTGTCATGGGCTTAATTCAGTATGCTATAGTCAACAAAGCTCTAGGGGAATGTCCCACAAAGAGAATGACGAGATTCTTCCGATCATGCCCTGACTGTATAGTTCCCATTGATGATACGTTGGAGGACTTGCCTGAGGTCCCTTCGCTAGATCTCATCCCTGAGAGGACCACAAACCCTTATCTCTACCTCAAGAAGGAGAAGATTGAACTGAATGTTAGGCATCGACTTGCAGTGATAAATGAGATCCGGGTAATAGCTAGGGATGAGATAGAAGAGACTCCCCTCTTGGCTAACAATACTCTGGAGGACGTGATGAGTTTGAGGGCAGCTCAGAGAATATTCTACACAGCCAAAGGAAAGGAGGCACAGTGGGACTTGCAGACTGCTGATCGAGAGGGGTACTTAAAACTGGATTTCGTGAGCGTAATCAGGAAGATTATAGGACACCTCTTTGTAATGGAATCAGAGACGTTAAAAGCCGGGTCAGACTACCCTACTTTCCAGTTACAGCAGAGAAGGATCATGCGGCGCGTTAGGCAGTCAGATACATCTAACTTTGTCCACTTAGGAGGGTTCTTCTGCTGGCAAGAGAGCATTGCGAGGATACAGAAACTGAAGTGGTCAGTGATGCCTGCCACATTCCCTATCACTGCAGAATCAGTGTCACTGGCAGCTAAGATGAGTCTAATAGGGGCAATGACTAGCGGGCTAACACCTAAGAGATGTGATGGTGTTCTGGCCGAAAACTTAATCCCGGATGTCACAAGGCAAGCAAAGAACATCATATGCCTGGACAAAGTGTTCCAAAGCAGATGTGATTACTGCTATACTGCAGCAATGACAAATAGGTGGTCAGACTCGATAAACTCCGACAGCATATTCAATATGAGATGTGAGAGAGGCCATACCATTCTGTCCCCGAGAATGTTGCATCAATTGAGGCGAACTATCTTGCCTGAAGGAGCCCTATACACTTTGGCTGTCAGAGTGGTTCACCAAAGCCCTCCTGAAGCATCACAACCTCTTCAGATCATCCCATGCAGGCGAGAGAGATATCAAATCTTGAGTGAAGCTGACATGCCTAGACAAACTGGCTACTCGGGGGTCTATAGAAGGCCTACCAATCAAATCTGCATGTATACAGAGGTTGAATTGGCAAAGAAGTACAGGTTGCCAACTAATTCATTATACAGGATCCTTGACTTACACGACTGCTTCTTCAGTCAGAAACTCATGGACACAAGGGGCAACATTTTAGTGGTCGGAGACGGGTATGGATACAGCTCCTTGCTAACCAAGTGCTTGAACCCAGACAGGAATGTAGTGAGCTGGACCTATATTGAACCATCTGAGGCACTTCCACATAGCCTGAGAATCTCTAAGCCTCCAATGCACTACAAAGCTGATGTGCAGATAGATTCTAGCCCTTCAATTGACAGAATCTCCGACATACATAACTCCTCATACCCGGATGAGTTCGCTAAAGTGGTGACCAAGAATGGGGTTACAGCACTGATCAGTGACATTGAAACTGTGTATGTCTCTGGAGAGAAGAGTGTGGCGTCTTTGATCAACTTGGCTTGGAATAACCAGATCCAATTAGGAGCACTCAAGTTGGAATTGATGACGGATCCCTTGGAGAGAGTCGTAGAATATGCCCACAATGCTTACCAAAGATGGGAATTATTCACTCTACCAGGCGCCAATCTCGGAGGAGGGGTACTATACATCGGTTTCTACGGTAGAAGAGAGAAGTTGACTGGGTACATCATTCCCCATTCAGGAGTTGAGACCTTGATGGATAGGTTGGCAACTGAGGTAGATGAGGATAGAGGAAGATTGAGAGCAGAGGACCTTGATCGGTGGGAGCAGTTGGATACAATGGAAAGCAAAATACATCTGCAGCAATACCATAAGATGAGGCTCGATCTATGGTTCGGATCTGCTTATCTGAGTGGTTATCTCTCAGAGGATATGACTGAGTTCTTTTACTCAGTAAAAACATCTTACAGACCACCTGCAATCTCATGGGAGAAAGGGAACCAGGCCAGATACCTCTATGGCAGCAGGGAAGAGATCTTATTCATGAGCATGATCACAGTGGCACTATCATCATATCTAGAGGACCAGGATGTCTTAAGAGAGTTCCTTTCTTCCTCAGGGTGGAAACTCCAGTGGAATAAGGATAAGAAGAATCCGAGGCATTGGTCCCCGTATTTGGAGCGGAGTGACTCTCAACTAGTCTGGAAAAAGAACAAAGCCATGATTTTCAAACTGGTATCAGTTCTGAGAGGATCAAAGCCGAGAGAAGAATATAAGATGGGAAAAGCATCTGACTCCATAGTTTTCAGATACATTCCGAGGAAGGTAAGGGAAGGAGAAGTTCCCCTGTGCTTCCCGATCTCTAAGGTGGCCTCAATGTCGGTATAAGAAAAACGAGACCTTCATCATGGATGATTTAATGGTCTATAGCCTTTGGGCTCTGCTGCAATGGGGTTTGCCTGCGGATACAGCCAGATACATAATCTCACTGAGGATGGAAGAAGACTACTGGGAAGAGATGAATGAACATGAGCAGTGGGACGGGTACGAGTCTTGGGATGATACTGATTGGCAGTATCCGAGTCCTTGAATAAGTCAGTATGTGACAATTTAGAAAAAACGATTACCTTACTGTTGACTTTAAAGAACACTGTTATGTTGTGTTCGCAACACACAACTTCCTT

>Seq10 Rice stripe mosaic virus HZ isolate 7, complete genome

AAGGAAGTTGCGTTGCGAACGCAACATTAAAATGTTGACATAACCCACCTTATCATAACAAAAGAAAAACGAGACCGCCAAGTCTTAAAATGGCAACCGACAAGTCTTTTGAGGAGAAGCTGAGCCTGGTTCCTGAGAACACTAAGCTATATTCAATATCTCCTGAGGCATATTCAGATGATAAGTTTGATAAGGCCAATTGCTACAAGCTAGAGAAGAGGTCTGAATATGAGCTAACTAGGCTCTACAAAGGACTAGTCCGGGACTTAGGGAATTCTTCCCCCTCCACTTATGCAGTCGAGAGATTGCTAGTCTTGGCAAGCCATCTGTATGAGACCAAGAAAGGGTCTTCTAACTTCTTCTTGACCGATTACCTGCCCAAGACAACATCAACAGCAAATCTTGATGCAGGCTTTTTGGCAAAGCTGAAAGAGACCCCTAAGGCCTCTGACCCCGACGTTTCCGATGTGACAGAAGTCAAGACAGCTAAGGCTACTCTAGACTCTGCTACAACTGATGCGGATACTAAAAAGGCCGCTTATGAGGCTATCGGAGATGAGGACTCGAAGAAGGCTGAGAAGGCTACAGCAAACACTGCCTGGATTGCTGCCCAAGAGGCCCAGAAGAAGGCTCAGAGTGCCTATGATAAGGCAGTTTCGAATGCGAAGAAGGCCTCAAGGAAAACTACCTCTGGAAAAAGCTTGTTCGGGGATGCAGGGGAGACAGTCACAGATGAGTCTAAGGTAGTGGAAGAGGTTGGAGAGGGGAAGAAGAAGTTTGGACCCTTCTTAGCTGCTTACTTGATGAGGCTGCTGACTAAAATAGCATCCAATGTCACAGAGTCATGGGAGCATATGAAAGGAATGTATAAGAACTTTTATGGTTATGATGCTCCTTCAGACCTGAACTGTCCCGAGGCAGGATTCCTTGAGCAACTGAAGTCTGAGCTTAACAAGGATAGAAGGGCGGCTACCTCATGGGTCAAAATAGTAGCAGAGGCAGATAATAAGTTGGATCAATCTACAGCCGAGGCTGGGATTCTCCGCTATGTGGCTGTCCTCCCTCTTGCATATTCTGGGATGCATGCGATGAAGTTGTTCATGGATGTCAAGATGCTGACCAAACTCACCAGCAACTACTTGATTGGTGCCATGAGGAGCCCTCTGACCAAGGATGCATTAGATGCAATCATGGATATCTTGATCTCGTTTGAGTCCACTACAAAGACTAAGAAGTCGGAGAAGTTCCGGTTTGCTAGGATTGTGAGTACTCAGTTCTTTCAATCCCTCCAGACCAAGAATTGCAAAGAGCTGGTCTATCTGATGGTTCAGATAATTGCTGAGTACAGGAAAGCAGAGGGGGTCAGGGATCCCATGAACATTGCTGGATTAGATGACATCTCTAGCAGGAACAAGAAGAAGTTGAATAAAGCAGTACGGATAATACTGGCAGAAGCTCCAAAGGCATCTGCAGGTGAGTATTCGTCAGCCATGAAGAAGGCTTTCCTGGATGACGAAGAGGATGATACAGCCAAGACCAGGTCCATCTTCCAGACCAAGGCTTAAGGAACATCATGTTTGGATAGAATATGACTCCTCTGAATAACTATGCAGCCTACTGGTGAGTCTTTGTGTGTTATATAAGAAAAACGAGACCGTCAGGTCAATAATGAGTGTGCCAGAGGATACTCCCTTCAGATCGTACTCCAGCATCTTTGACGACTCCGACTTTGTCCAACCACAGCCCATGTCCTTCAAAGCGACCAAGGAGTCAGAGAGCCTTCCTGAGACAGAGAAAGAAGATATGTCGACTGAATACCTCTCTGAACCACTGAGGACCAAGTCAGGTAAGAAGAACAGACGGAGGAAGGGAAAGGACCTAAAATCTCTCTTCACTCAAGAAGCTGGACTCCCGGCTCCAGAAGCAGACAGTGTCCTCCCCGAATCGTCTCCGTATGAGAATGATAACGCACAGTTGGAGCTACCCAAGCCCATTCTAAAGACATCAGACGCTCCGGTCTTCCTAAGAGAGAAGGATCTTAGTAAAGAATTTGCTGCAGCCTGCAAGACTAATGGGATTCTCCCAAGGGATGAATGGAAGTCATCTGTAGCAGCCAAGTATCATGCAGAGGAAGGGAAAATGACCAAACGAGACATCTCGTTAATCGTATTTGGAATGGAGCTTTACAAGAGATACAATGTGGAATCTGAGGTATCAACTTTGTTCACTTCATTGGTGACTGAGTTGCAGGGGATAAAGGTTGCTGCTAAGGAGTTGAATGATACCCGGGAGGTCCTCACTAAGATTCCAGGAGAAATTGTGTCTGCTGTCAAGGCAGGGGTAAAGGAGGGGACCGAGATGGGGATGGATTATATAGAGACTAGAACTAAAGTGGCCCCCAAGAGTGCTCCTAAAGTGGACATCTCCAAGCCGATGAGTAGTAAGATGATGGAGCAGCAGGATGAGAGTTCTGATGAGTCCTCAGATAATGAGAGTGAGGAGAGTGAGGAAGAATCCTTTGAGACAAAGGCTGCAATCTTCTTAGCTTTGATAAAGGTTCCGGAGGAAGAGAGAGACAACGCAATAGTCCTTATGGCCTTGAGGGCAGTGATATCTGACAGTGAGCTGAATCAGGCAATTAGGAATGACAGAATCTCCTCCTCAGTAGCAGATATGTACCATCAAAAGATATCTGACAAGGCTAGGGAATTGATGGGAAAGGGAAAGACCAACAAGAGGGCCAAGCAGCCTAAATCCTCTAAGTATGCATCGGATTACTATGATGATGCACTGTGATTGATCAGTTACTACCTGTGTGGCAATGTCGGAACTGTACTTACCTATATATTGATAATCGTCTGTGATCTTGTGCCTTTAATTGCTGCAGTATTATAGCTTAAAATAATCTAGTTACCAATGCTCTATTTTCGTACTTAAGTGCCATGTTGCCTGCCTTTACCAAAGTAGTCTAGTGTGCTCTATTTAAGAAAAACGAGACCTTCATCATGAAGATCATCTGCAGTACTGGGATCTTCAATGAAGAGAAAGGGTTCCCCCTCCCCAACCTCCTCAACAGTCCCTTGATGAAGCAGGAGATCATGACGGTCAAGTACTTGAGGTTTCAGTATATCCCTATTATGACCAGCAACCCCTCGAGTTCATTGACTATTGACATTAAAGATACTCGACTGGTCAATTGGGATAATAGGTCGATTTTCCAGGTCAAAATATTCGGGGATGTTCAAAGCTCATTTATTGTATCGGGTCTACAGCCTTATTCAGCTAGAGACCGTTGCCCTTATCTCCTCTCCCTTTCAGTAAACGCAGGGAAGGTTGTTCCAGGGACCAAATATGGCATCTTAAAATCTTATGCTGTGTACACATCTAAAGACTCAGGGATAATTTCGTCACAGATCTCTGTAAAACTTGAAAGGTCCCCACGTGACTACTTCTTGAAGAGGTCTAAGGAGCACGACAAAAAAGATCTTGACAGTGATGTCTCATTCAAGATGTGTCGCCATGTCAAGTTTGCTACTTGAAGCTTGGATAGGATGGGCAGCTGAATGTTGTCGGGGTGATGTTTTCCTTATGAATATCTTCAATCATTTCCCTGGAGGGAGAAACAGTTGATCTCTGGGAGGACGCAAGGGCTCACTACTAGCATGTGCTTGCCAGGTTGGGGTTTTATGCCTATCATGGTTTAGCCTAAATAAGAAAAACGAGACCATCATGGCCGTTCCGTGGACTGAGGTTAAAGACTCCAAGTACTTGGCAACTAAGATGTCTGTTACCCTGATCATGGAGATGAATGAAGATACACCTCTAAAGTATCCTTCTTACAACGCATTCGAGAGCATCTTTAAAAGGCTAGCAGAACCAGAATGCGCAGCACCCCAAGTGGCCGCATGGTTTACTTGGTTCCTAAGAGAGGCAAAGGACATCTACTATCTGGAAGTGAGTAACAAGGAAACTGCCCAATACGGACCAACTAAAGTCTATAAACTTCAGTGTCCAGCCTACCTCTTATCCCGAGTGACAGGAGGATCACAGCTTGATTATACCTCTCTTATAGGTAGCAAGGTGATGACTGAGAAAGATCGGGGGATCCCAGTCAGAACACTCTATATTACTGGAGGGGGTACCTCATTCAGAGTCATCAACGAGGAGACAGCCAACCAGTTCATCATAAATGATAATGCTGTCCGCCTACCAGGAGAGTGCAAGGTGGATGGAGGAAGTATAATCTGGAGTTAGAATTTCAAGAAAACTAAACAATAATGAACAAGTGGAACAATCTCGTGTAGTGTATTAGGTGTCCTCTTTAACTAAAGTTAAATAAGAAAAACGAGACCCGCAAGGATCTTAAGATAACAAGATGATGAGGATTTCGGTCTTTCTCTTGATGCTCTGCTGGCTCCCTGTCAGCCTGACCTTCTTCGACAAATCACACATTCCTATAACCACATGTGATAAGAATCTAATGAGTCCTATCCCTTGGAGAACTTACTGCATAGAGGAGTGTGGAATCCGGAATGTAATAGGTGATAAGCTGGATCTGTTTATCTACAATAGGTCTGACAGTGGGAAAGTACAGCTGGCTGACTGCAGGAAGTACAAGATCAGACAGACCTTCACCAAGACTTGGACATTCTCGACATTCAAAGGGGCGATAGAGACAGAGGAGCTAATGCCTAATTATGCAGAGTGTGAATCCACCTGGAGAGATCTGTGCAACTCTGGACCGTGTAGTACCACAACTCCGGTGATCCCTGAGGAATACCATTGGGCTTCTGACACCACGAAGGAGGTCATCTATGTATCTATAGATGCATACCAGAAAACTGTTGCATTCCAGGATCCTAGTGGTGATATCCAGCTCCTAGTCCATGGGGTGATCATAGATGGGAGCCAGTCTGGTTATGTTCAACCCAGCAAAGATCTCATCACTATGTGGGATAAGGTTGAGTTACAAGATGAATGCCCTTGGTCAACGGGAAATTCTCTTTCGTGTTCTACGTCTGATGAGGGAATTTCATACTACTGTGCTGGGAAAGGGCTAGTACTGACCAACATCAGTACGGTGACTGATACCAGATGTGACAACAACCCGCACCTAATGACATCAGGGCACCATGTGATTTTTAGGGTGAAGAAGGCATCAGACCCGAATGCGACTCTCAGCAGGACAGCTCAAATAGTGCTGGACAGGGGGTCAGAAGAGGCCGAGATTGTAGATAGTGTTAATAAGGCGTTGCTGGATAGAGATTCCATCAGGTGTGCAAGCTCATGTCTCGCCTTTGATTACACCATCTCCAAGCCTCAGATGTTTGGCAACCAATTGGCGCTACCTTATAAGGGGTCTTTTCTCCCTTGCAACATACTGCCTAATTGTCGGGTTGTCTTCCCAGTCAAGTATTGCAGCTCTCCTCCGATGATTCTGGTAGAATGTACCGGCACTATGACATGGTGGAATATTACTGGAGATTACACGATCAGACCCACCTATTGCCACATGAACCAGTCGGCGACCAAGATTAAGACATCTATATCCTTTATGACAACAAATGGGAGAGTCTTAGTGAATGAGTCTGGCGCTTATCCTGTCTCCCGTGAAATAGGAAATACGTTCCAGGTCGGACATGTCATAGAGCCTAGCTCCATGATAGAGGTGACTGATCCACTTAATGTTAGGATAGATGACACCTTAGTCACACCAGAGTCCCATACTATATCTAATATCACTTCAGTGGGGGACTCACTTTTGGATACAATGGTTGAGACCGTGAAAGGTATCGGTCGCTTCATATCCCATGAGGTCAGGATAGTGGTTTTTGGTGTTCTGACTCTTTTTATATTATATCTGTCGTTTAAATATTTGTTCGCTAAGAAGAAGAGCAGAGTGCCGCACCCTAAAGTTGTCTATACAAAACCCACATCTGAAGGACCAGTGATCTATGACACTGAATATACTATAGAAAGTGACTAATAAAAAACAGAGACCAACATGGAGTTCAATTGGCCTTGGGGACAGAACAGTGAAACGGAAATCACCAAGAATCTCCGCTTTGAGGACATTAAGGTGATGGCCATAATAATACTAGTCTGGGTGAAGTGTCTTCTCATCTACCATTTCAAGAGGAAAATAAGGCGACTAAGATCTCTATTGATAAAAGGATCCTCACAATGGGTACTGCATGATGCCTAACTCATTAGGAAGATCTGATATGAGTTTTTATCCTGCCTTATCCTTCTAATTAAGAAAAACGAGACCGCCATCATGGACCTTGATGACGGTGGTCTATGGAGACGTGCTAGGGGTCTGGGGGATTATCACCTGAGGTCTGCTCTGGTGACCCCCTCCTTAGAGCGTTTCCGCAGTCGAAAGGGAAGGCACCGTGAACAACTGTGCTTTGATAGGATGAAATCACTAGGCTGGATGTTGAGGTGGGTAGATCAGGGGAAACTGCTTGGATATTTAATGGTAGAAGCCAACAAATCTTTACCAAAATCCATTGCAAACCAGGAACTCCTAGTTGAGACTCTGAAGTTGGAATACGGATGTCTTAGACAGATAATCATGACGGATGGAGACCTCCATGATCAGGTGATCTCTTATCTAGACAGGAAGTCTATCTCTACCCATTACACCCATGGTAGGGAGGTCTTTCAGGAAGCCTTGATAGTTGTCATGGCACTTTCTTCAGGGAGAGAACCACCAGATCATGTCAATAACTTGGGTTATGAAATGCTGAATGAAGAACTTGAAGTGCCCGTGGTCAGAACCTATGGCGTAATCTTCTATCTCTTTGGGGACTTGATCTACGTAAAGTATCCGGAGGAAGAGGGCATGATCACACTGGACATGTTCAGAAACCTGACAGATAAATTCTCTGAGAGGGAGAATATTATGATTGCAACTCAACTAGGAACTGAGATCCTTCAAGAGATATACCCTTCTGAGACAGTGCTCAAAACAGTCTTTTCTCTTTGGGACAAAGGTCTATTGAAAGAGGGAAATGACTTTTACACAGTGGTAAAGACATTTGAAGCAATCATAAATGGGATGTTGATCAAGAACAATGACGGAACATACTATGATCCTTCAGCATACCTCCGAGAAACTATCATGGGGCTGCCAGTACGACTGAGGGATTATGCACAGACGTTAGTGAGTTACTTAGACTCTCTCCCCTTTAATCCACACCACCTATCTCAGATTGGGGGGTTGTTTCGGTTGTGGGGACACCCCATAGTAGATCCTAACGCTGGGGTCAGAAAGGTAAGGCTTCTGGGAACAGCTGATAAGATGAACCTGACACATATCCCCACCCTAGCAGAGCGGAAATTCAAGGAGATCTTCTACCTGTCGTATTATGAAAAGCATAGAGTCTATCCTAATCATAACTTGAATGGAGAAATTGAAGGAAGCTATTTGCTCTCTCAGCTTGCACAAATGGCTCTGGTTAATCCCAAGCATGCAAATTACTCACTAGTAGACTGGGATTCAGTCAACACACTAGAGACCTTTCCCTTTCCTAAATCATTCAACCTGTCTCTTATTATAGCGGATAAAGCTGTAAGTCCGAACAGAGAAGAATGGTTGGAATTGAGACGTAAGGGAGGAACTCAGATGGATCCGCACATTAGAAGAGGGCCTCTGAAGGCTATGAAAGACGGAGTAATCGATTGTGAGAAACTATTGAGGAAGATAGACCGTAACCCGTCCGGTTTAGCTAAGAAACATAGGATCATTGGACTGTACCCAAAGGAGAGAGAAGAAAATATGGTTCCCAGGATGTTTGCATTAATGTCCTTTGACATGAGAGCTTTCTCAGTGGTCTCTGAATCCATGATAGCAGATCACATCATTCCTCATATAGAAGGTGTCACAATGACGAAGAGCATGTTGGCCCTCCAGAAAGAGATGATCATTTCAACTAAAAGCCAGGCCTCCTCAACCCAGTCAGACAGTATAACCTTCTGTCTTAATATTGACTTTGAAAAGTGGAACCTTAACTTCCGAAGATGGATGACGGAGGGGGTATTCAGAGAAATGGGGCGATTGTTCGGACTTCCAGAGATATTCAATCGGACTTATGACATCTTCAAGAAATCCATCATTTATCTAGCAGATGGAAGCTTCGACCTCTTGCTGACAGATGAGTTGGAAATAGAACCAGGGACCAATCCAGATTGCGCATACACCGGTCATGTTGGGGGGTTTGAGGGATTGAGGCAGAAAGGATGGACAGTCTTCACAGCGGTCTTAATCTCTTCCATCTGCGATGAAATGGGGATCAAGACTCATCTAATGGGGCAAGGTGATAACCAGGTTTTGATGCTGACCATCTACTCAAGAGCTGCTAGAGAGACGGGGGATTTGAAATCAGCACCAGCAGTACTGGAGATCACAAATACATTGGAGACATTCAAGAGCAGACTAGTCTCACTCTTTGCTAATCTTGGATTGCCTATAAAACCACTAGAGACCTGGGTCTCAGAGGAATTGTTTGCCTATGGCAAGACTCCAATATACCGAGCAGTCCCCCTTGCAATGAGTCTCAAGCGAATCTCTAGAGTTTTTGCGTTTTCCAATGAGGATCTAATGACCTTGTCTAATGCGTTAGGTGCTATCTCTGCGAATGCACAAGCTGCATCCATGTGCGATGTCCATCCGATGGTGTCATATGCCATAGCAAAGTGGCAACACTTATGCTGTGCTATAATCTTCTCCAACTATCATCCGCTATGCGGGTGTGCTCCACATGTCTCAGGAGAGGAATGGGCAATAAAGCTACGACTTCCGTCAGGAAAGAAGATTCAAGAGACATCTGATGAGGAGATAGACGAAAGAGATCTGATGAAACTAATAGTGACCATACCTCGAAGCTTAGGAGGTTATAATACCCTGACTCTGTATGAGATGATCATGAGAGGATTTTCTGACCCAGTCTCTAGGGACATGTGCTGGCTATTCGCAATTGCCAGTGAATCAACAGGAAAACTTCGAGGTTATCTGATAAACTGGATTAAACCAATCGTGTCTCCGGAAGTAAATGCTCAGCATCTCATTCAGGATCCCACGGCACTCAATTTGCTAGTACCCCCTAATGCTACATCAGTTATTAAGAGGATGATAGACAAATCACTCGAAGCACTCCCAAAGAGATCTCGGTTCGCAGTATGGTTCTCTGAGATCCTAGAGATCTCAGGGGACAAGGAAATCTCAAAGTTAGCAGAGGCCTTGACCAGGACAGATTCCCTGAACCCCCGTTTCCTTCACGACATTCTGGGGGCCACTCTTTATGGATACTGCACGGCTATAACTAGTAAAGTTGACAAGACAGTGACCTTATCTCGGATGGCACTAGCGTCTAAGGATGTTGTTGGAGCTCTAATCAAGGGGGAGATGAGACTTTACTCCTATTTCGGATGGAGAACCTTGCAATCGAGAGGCTTACCTCTGACCACCAGATGTCCCAATAAGTGGGTGCGGATAATTAGGGATATCTCATGGCAAAAACAAATTAAGGCTGTTAGCGTTCCGTATCCTACTCATTTCTTATCAGAGGATATTTCAGAGACTGATAGGCCCGATTCCTGGATTGAGTGCTATATTGATGATGCCCCGACATCTGACAGAAGTTGCATGATCTATTCGACAGGAAAAGCTCTGCCGTACTTGGGTAGTGTCACTAGAGAAAAACTGACAACTCGAGGTGCAAAGGCGGCCTATGGAACTGAACCTCTTGTATTGAGGCCAATTAATCTAGTCAGAACTATAGGTTGGTTCATTGAAGAGGATAGCAATTTTGCTGAATTGATCAAGATGCTACTGGGAGCCGTCACAGATCTTCCCATAGAGGAGGTACTCTATATCCCAGAAATGGTGAGTGGCTCTATGGCACATAGATACCTAGATATGTCAACACAGCATGGATCTTTGTGGATGCCTCTTTATGGTCCTGCCACTTTTCTCCATATGAGCACTAATACTTTTGTGCAGTACCTGAAGGGGACAGAAAATGTCACTTTGCACTTTCAGTGTGTCATGGGCTTAATTCAGTATGCTATAGTCAACAAAGCTCTAGGGGAATGTCCCACAAAGAGAATGACGAGATTCTTCCGATCATGCCCTGACTGTATAGTTCCCATTGATGATACGTTGGAGGACTTGCCTGAGGTCCCTTCGCTAGATCTCATCCCTGAGAGGACCACAAACCCTTATCTCTACCTCAAGAAGGAGAAGATTGAACTGAATGTTAGGCATCGACTTGCAGTGATAAATGAGATCCGGGTAATAGCTAGGGATGAGATAGAAGAGACTCCCCTGTTGGCTAACAATACTCTGGAGGACGTGATGAGTTTGAGGGCAGCTCAGAGAATATTCTACACAGCCAAAGGAAAGGAGGCACAGTGGGACTTGCAGACTGCTGATCGAGAGGGGTACTTAAAACTGGATTTCGTGAGCGTAATCAGGAAGATTATAGGACACCTCTTTGTAATGGAATCAGAGACGTTAAAAGCCGGGTCAGACTACCCTACTTTCCAGTTACAGCAGAGAAGGATCATGCGGCGCGTTAGGCAGTCAGATACATCTAACTTTGTCCACTTAGGAGGGTTCTTCTGCTGGCAAGAGAGCATTGCGAGGATACAGAAACTGAAGTGGTCAGTGATGCCTGCCACATTCCCTATCACTGCAGAATCAGTGTCACTGGCAGCTAAGATGAGTCTAATAGGGGCAATGACTAGCGGGCTAACACCTAAGAGATGTGATGGTGTTCTGGCCGAAAACTTAATCCCGGATGTCACAAGGCAAGCAAAGAACATCATATGCCTGGACAAAGTGTTCCAAAGCAGATGTGATTACTGCTATACTGCAGCAATGACAAATAGGTGGTCAGACTCGATAAACTCCGACAGCATATTCAATATGAGATGTGAGAGAGGCCATACCATTCTGTCCCCGAGAATGTTGCATCAATTGAGGCGAACTATCTTGCCTGAAGGAGCCCTATACACTTTGGCTGTCAGAGTGGTTCACCAAAGCCCTCCTGAAGCATCACAACCTCTTCAGATCATCCCATGCAGGCGAGAGAGATATCAAATCTTGAGTGAAGCTGACATGCCTAGACAAACTGGCTACTCGGGGGTCTATAGAAGGCCTACCAATCAAATCTGCATGTATACAGAGGTTGAATTGGCAAAGAAGTACAGGTTGCCAACTAATTCATTATACAGGATCCTTGACTTACACGACTGCTTCTTCAGTCAGAAACTCATGGACACAAGGGGCAACATTTTAGTGGTCGGAGACGGGTATGGATACAGCTCCTTGCTAACCAAGTGCTTGAACCCAGACAGGAATGTAGTGAGCTGGACCTATATTGAGCCATCTGAGGCACTTCCACATAGCCTGAGAATCTCTAAGCCTCCAATGCACTACAAAGCTGATGTGCAGATAGATTCTAGCCCTTCAATTGACAGAATCTCCGACATACATAACTCCTCATACCCGGATGAGTTCGCTAAAGTGGTGACCAAGAATGGGGTTACAGCACTGATCAGTGACATTGAAACTGTGTATGTCTCTGGAGAGAAGAGTGTGGCGTCTTTGATCAACTTGGCTTGGAATAACCAGATCCAATTAGGAGCACTCAAGTTGGAATTGATGACGGATCCCTTGGAGAGAGTCGTAGAATATGCCCACAATGCTTACCAAAGATGGGAATTATTCACTCTACCAGGCGCCAATCTCGGAGGAGGGGTACTATACATCGGTTTCTACGGTAGAAGAGAGAAGTTGACTGGGTACATCATTCCCCATTCAGGAGTTGAGACCTTGATGGATAGGTTGGCAACTGAGGTAGATGAGGATAGAGGAAGATTGAGAGCAGAGGACCTTGATCGGTGGGAGCAGTTGGATACAATGGAAAGCAAAATACATCTGCAGCAATACCATAAGATGAGGCTCGATCTATGGTTCGGATCTGCTTATCTGAGTGGTTATCTCTCAGAGGATATGACTGAGTTCTTTTACTCAGTAAAAACATCTTACAGACCACCTGCAATCTCATGGGAGAAAGGGAACCAGGCCAGATACCTCTATGGCAGCAGGGAAGAGATCTTATTCATGAGCATGATCACAGTGGCACTATCATCATATCTAGAGGACCAGGATGTCTTAAGAGAGTTCCTTTCTTCCTCAGGGTGGAAACTCCAGTGGAATAAGGATAAGAAGAATCCGAGGCATTGGTCCCCGTATTTGGAGCGGAGTGACTCTCAACTAGTCTGGAAAAAGAACAAAGCCATGATTTTCAAACTGGTATCAGTTCTGAGAGGATCAAAGCCGAGAGAAGAATATAAGATGGGAAAAGCATCTGACTCCATAGTTTTCAGATACATTCCGAGGAAGGTAAGGGAAGGAGAAGTTCCCCTGTGCTTCCCGATCTCTAAGGTGGCCTCAATGTCGGTATAAGAAAAACGAGACCTTCATCATGGATGATTTAATGGTCTATAGCCTTTGGGCTCTGCTGCAATGGGGTTTGCCTGCGGATACAGCCAGATACATAATCTCACTGAGGATGGAAGAAGACTACTGGGAAGAGATGAATGAACATGAGCAGTGGGACGGGTACGAGTCTTGGGATGATACTGATTGGCAGTATCCGAGTCCTTGAATAAGTCAGTATGTGACAATTTAGAAAAAACGATTACCTTACTGTTGACTTTAAAGAACACTGTTATGTTGTGTTCGCAACACACAACTTCCTT

>Seq11 Rice stripe mosaic virus LS isolate, complete genome

AAGGAAGTTGCGTTGCGAACGCAACATTAAAATGTTGACATAACCCACCTTATCATAACAAAAGAAAAACGAGACCGCCAAGTCTTAAAATGGCAACCGACAAGTCTTTTGAGGAGAAGCTGAGCCTGGTTCCTGAGAACACTAAGCTATATTCAATATCTCCTGAGGCATATTCAGATGATAAGTTTGATAAGGCCAATTGCTACAAGCTAGAGAAGAGGTCTGAATATGAGCTAACTAGGCTCTACAAAGGACTAGTCCGGGACTTAGGGAATTCTTCCCCCTCCACTTATGCAGTCGAGAGATTGCTAGTCTTAGCAAGCCATCTGTATGAGACCAAGAAAGGGTCTTCTAACTTCTTCTTGACCGATTACCTGCCCAAGACAACATCAACAGCAAATCTTGATGCAGGCTTTTTGGCAAAGCTGAAAGAGACCCCTAAGGCCTCTGACCCAGACGTTTCCGATGTGACAGAAGTCAAGACAGCTAAGGCTACTCTAGACTCTGCTACAACTGATGCGGATACTAAAAAGGCCGCTTATGAGGCTATCGGAGATGAGGATTCAAAGAAGGCTGAGAAGGCTACAGCAAACACTGCCTGGATTGCTGCCCAAGAGGCCCAGAAGAAGGCTCAGAGTGCCTATGATAAGGCAGTTTCGAATGCGAAGAAGGCCTCAAGGAAAACTACCTCTGGAAAAAGCTTGTTCGGGGATGCAGGGGAGACAGTCACAGATGAGTCTAAGGTAGTGGAAGAGGTTGGAGAGGGGAAGAAGAAGTTTGGACCCTTCTTAGCTGCTTACTTGATGAGGCTACTGACCAAAATAGCATCCAATGTCACAGAGTCATGGGAGCATATGAAAGGAATGTATAAGAACTTTTATGGTTATGATGCTCCTTCAGACCTGAACTGTCCCGAGGCAGGATTCCTTGAGCAACTGAAGTCTGAGCTTAACAAGGATAGAAGGGCGGCTACCTCATGGGTCAAAATAGTAGCAGAGGCAGATAATAAGTTGGATCAATCTACAGCCGAGGCTGGGATTCTCCGCTATGTGGCTGTCCTCCCTCTTGCATATTCTGGGATGCATGCGATGAAGTTGTTCATGGACGTCAAGATGCTGACCAAACTCACCAGCAACTACTTGATTGGTGCCATGAGGAGCCCTCTGACCAAGGATGCATTAGATGCAATCATGGATATCTTGATCTCGTTTGAGTCCACTACAAAGACTAAGAAGTCGGAGAAGTTCCGGTTTGCTAGGATTGTGAGTACTCAATTCTTTCAATCCCTCCAGACCAAGAATTGCAAAGAGCTGGTCTATCTGATGGTTCAGATAATTGCTGAGTACAGAAAAGCAGAGGGGGTCAGGGATCCCATGAACATTGCTGGATTAGATGACATCTCTAGCAGGAACAAGAAGAAGTTGAATAAAGCAGTGCGGATAATACTGGCAGAAGCTCCAAAGGCATCCGCAGGTGAGTATTCGTCAGCCATGAAGAAGGCTTTCCTGGATGACGAAGAGGATGATACAGCCAAGACCAGGTCCATCTTCCAGACCAAGGCTTAAGGAGCATCATGTTTGGATAGAATGTGACTCCTCTAAATAACTATGCAGCCTACTGGCGAGTCTTTGTGTGTTATATAAGAAAAACGAGACCGTCAGGTCAATAATGAGTGTTCCAGAGGATACTCCCTTCAGATCGTACTCCAGCATCTTTGACGACTCAGACTTTGTCCAACCACAGCCCATGTCCTTCAAAGCGACCAAGGAGTCAGAGAGCCTTCCTGAGACAGAGAAAGAAGATATGTCGACTGAATACCTCTCTGAACCACTGAGGACCAAGTCAGGTAAGAAGAACAGACGGAGGAAGGGAAAGGACCTAAAATCTCTCTTCACTCAAGAAGCTGGACTCCCGGCTCCAGAAGCAGACAGTGTCCTCCCCGAATCGTCTCCGTATGAGAATGATAACGCACAGTTGGAGCTACCCAAACCCATTCTAAAGACATCAGACGCTCCGGTCTTCCTAAGAGAGAAGGATCTTAGTAAAGAATTTGCTGCAGCCTGCAAGACTAATGGGATTCTCCCAAGGGATGAATGGAAGTCATCTGTAGCAGCCAAGTATCATGCAGAGGAAGGGAAAATGACCAAACGAGACATCTCGTTAATCGTATTTGGAATGGAGCTTTACAAGAGATACAATGTGGAATCTGAGGTATCAACTTTGTTCACTTCATTGGTGACTGAGTTGCAGGGGATAAAGGTTGCTGCTAAGGAGTTGAATGATACCCGGGAGGTCCTCACTAAGATTCCAGGAGAAATTGTGTCTGCTGTCAAGGCAGGGGTAAAGGAGGGGACCGAGATGGGGATGGATTATATAGAGACTAGAACTAAAGTGGCCCCCAAGAGTGCTCCTAAAGTGGACATCTCAAAGCCGATGAGTAGTAAGATGATGGAGCAGCAGGATGAGAGTTCTGATGAGTCCTCAGATAATGAGAGTGAGGAGAGTGAGGAAGAATCCTTTGAGACAAAGGCTGCAATCTTCTTAGCTTTGATAAAGGTTCCAGAGGAAGAGAGAGACAACGCAATAGTCCTTATGGCCTTGAGGGCAGTGATATCTGACAGTGAGCTGAATCAGGCAATTAGGAATGACAGAATCTCCTCCTCAGTAGCAGATATGTACCATCAAAAGATATCTGACAAGGCTAGGGAATTGATGGGAAAGGGAAAGACCAACAAGAGGGCCAAGCAGCCTAAATCCTCTAAGTATGCATCGGATTACTATGATGATGCACTGTGAGTAATCAGTTACTACCTGTGTGGCAATGTCGGAACTGTACTTACCTATATATTGATAATCGTCTGTGATCTTGTGCCTTTAATTGCTGCAGTATTATAGCTTAAAATAATCTAGTTACCAATGCTCTATTTTCGTACTTAAGTGCCATGTTGCCTGCCTTTACCAAAGTAATCTAGTGTGCTCTATTTAAGAAAAACGAGACCTTCATCATGAAGATCATCTGCAGTACTGGGATCTTCAATGAAGAGAAAGGCTTCCCCCTCCCCAACCTCCTCAACAGTCCCTTGATGAAGCAGGAGATCATGACTGTCAAGTACTTGAGGTTTCAGTATATCCCTATTATGACCAGCAACCCCTCGAGTTCATTGACTATTGACATTAAAGACACTCGACTGGTCAATTGGGATAATAGGTCGATTTTCCAGGTCAAAATATTCGGGGATGTTCAAAGCTCATTTATTGTATCGGGTCTACAGCCTTATTCAGCTAGAGACCGTTGCCCTTATCTCCTCTCCCTTTCAGTAAACGCAGGGAAGGTTGTTCCAGGGACCAAATACGGCATCTTAAAATCTTATGCTGTGTACACATCTAAAGACTCAGGGATAATTTCGTCACAGATCTCTGTAAAACTTGAAAGGTCCCCACGTGACTACTTCTTGAAGAGGTCTAAGGAGCACGACAAAAAAGATCTTGACAGTGATGTCTCATTCAAGATGTGTCGCCATGTCAAGTTTGCTACTTGAAGCTCGGAGAGGATGGGCAGCTGAATGTTGTCGGGGTGATGTTTTCCTTATGAATATCTTCAATAATTTCCCTGGAGGGAGAAACAGTTGATCTCTGGGAGGACGCAAGGGCTCACTACTAGCATGTGCTTGCCAGGTTGAGGTTTTATGCCTATCATGGTTTAGCCTAAATAAGAAAAACGAGACCATCATGGCCGTTCCGTGGACTGAGGTTAAGGACTCCAAGTACTTGGCAACTAAGATGTCTGTTACCCTGATCATGGAGATGAATGAAGATACACCTCTAAAGTATCCTTCTTACAACGCATTCGAGAGCATCTTTAAAAGGCTAGCAGAACCAGAAAGCGCAGCACCCCAAGTGGCCGCATGGTTTACTTGGTTCCTAAGAGAGGCAAAGGACATCTACTATCTGGAAGTGAGTAACAAGGAAACTGCCCAATACGGACCAACTAAAGTCTATAAACTTCAGTGTCCAGCCTACCTCTTATCCCGAGTGACAGGAGGATCACAGCTTGATTATACCTCTCTTATAGGTAGCAAGGTGATGACTGAGAAAGATCGAGGGATCCCAGTCAGAACACTCTATATTACTGGAGGGGGTACATCATTCAGAGTCATCAACGAGGAGACAGCCAACCAGTTCATCATAAATGATAATGCTGTCCGCCTACCAGGAGAGTGCAAGGTGGATGGAGGAAGTATAATCTGGAGTTAGAATTTCAAGAAAACTAAACAATAATGAACAAGTGGAACAATCTCGTGTAGTGTATTAGGTGTCCTCTTTAACTAAAGTTAAATAAGAAAAACGAGACCCGCAAGGATCTTAAGATAACAAGATGATGAGGATTTCGGTCTTTCTCTTGATGCTCTGCTGGCTCCCTGTCAGCCTGACCTTCTTCGACAAATCACACATTCCTATAACCACATGTGATAAGAATCTAATGAGTCCTATCCCTTGGAGAACTTACTGCATAGAGGAGTGTGGAATCCGGAATGTAATAGGTGATAAGCTGGATCTGTTTATCTACAATAGGTCTGACAGTGGGAAAGTACAGCTGGCTGACTGCAGGAAGTACAAGATCAGACAGACCTTCACCAAGACTTGGACATTCTCGACATTCAAAGGGGCGATAGAGACAGAGGAGCTAATGCCTAATTATGCAGAGTGTGAATCCACCTGGAGAGATCTGTGCAACTCTGGACCGTGTAGTACCACAACTCCGGTGATCCCTGAGGAATACCATTGGGCGTCTGACACCACGAAGGAGGTCATCTATGTATCTATAGATGCATACCAGAAAACTGTTGCATTCCAGGATCCTAGTGGTGATATCCAGCTCCTGGTCCATGGGGTGATCATAGATGGGAGCCAGTCTGGTTATGTTCAACCCAGCAAAGATCTCATCACTATGTGGGATAAGGTTGAGTTACAAGATGAATGCCCTTGGTCAACGGGAAATTCTCTTTCGTGTTCTACGTCTGATGAGGGAATTTCATACTACTGTGCTGGGAAAGGTCTAGTACTGACCAACATCAGTACGGTGACTGATACCAGATGTGACAACAACCCGCACCTAATGACATCAGGGCACCATGTGATTTTTAGAGTGAAGAAGGCATCAGACCCGAATGCGACTCTCAGCAGGACAGCTCAAATAGTGCTGGACAGGGGGTCAGAAGAGGCCGAGATTGTAGATAGTGTTAATAAGGCGTTGCTGGATAGAGATTCCATCAGGTGTGCAAGCTCATGTCTCGCCTTTGATTACACCATCTCCAAGCCTCAGATGTTTGGCAACCAATTGGCGCTACCTTATAAGGGGTCTTTTCTCCCTTGCAACATACTGCCTAATTGTCGGGTTGTCTTCCCAGTCAAGTATTGCAGCTCTCCTCCGATGATTCTGGTAGAATGTACCGGCACTATGACATGGTGGAATATCACTGGAGATTACACGATCAGACCCACCTATTGCCACATGAACCAGTCGGCGACCAAGATTAAGACATCTATATCCTTTATGACAACAAATGGGAGAGTCTTAGTGAATGAGTCTGGCGCTTATCCTGTCTCCCGTGAAATAGGAAATACGTTCCAGGTCGGACATGTCATAGAGCCTAGCTCCATGATAGAGGTGACTGATCCACTTAATGTTAGGATAGATGACACCTTAGTCACACCAGAGTCCCATACTATATCTAATATCACTTCAGTCGGGGACTCACTTTTAGATACAATGGTTGAGACCGTGAAAGGTATCGGTCGCTTCATATCCCATGAGGTCAGGATAGTGGTTTTTGGTGTTCTGACTCTTTTTATATTATATCTGTCGTTTAAATATTTGTTCGCTAAGAAGAAGAGCAGAGTGCCGCACCCGAAAGTTGTCTATACAAAACCCACATCTGAAGGACCAGTGATCTATGACACCGAATATACTATAGAAAGTGACTAATAAAAAACAGAGACCAACATGGAGTTCAATTGGCCTTGGGGACAGAACAGTGAAACGGAAATCACCAAGAATCTCCGCTTTGAGGACATTAAGGTGATGGCCATAATAATACTAGTCTGGGTGAAGTGTCTTCTCATCTACCATTTCAAGAGGAAAATAAGGCGACTAAGATCTCTATTGATAAAAGGATCCTCACAATGGGTACTGCATGATGCCTAACTCATTAGGAAGATCTGAGATGATTTTTTATCCTGCCTTATCCTTCTAATTAAGAAAAACGAGACCGCCATCATGGACCTTGATGACGGTGGTCTATGGAGACGTGCTAGGGGTCTGGGGGATTATCACCTGAGGTCTGCTCTGGTGACCCCCTCCTTAGAGCGTTTCCGCAGTCGAAAGGGAAGGCACCGTGAACAACTGTGCTTTGATAGGATGAAATCACTAGGCTGGATGTTGAGGTGGGTAGATCAAGGGAAACTGCTTGGATATTTAATGGTAGAAGCCAACAAATCTTTACCAAAATCCATTGCAAACCAGGAACTCCTAGTTGAGACACTGAAGTTAGAATACGGATGTCTTAGACAGATAATCATGACAGATGGAGACCTCCATGATCAGGTGATCTCTTATCTAGACAGGAAGTCTATCTCTACCCATTACACCCATGGTAGGGAGGTCTTTCAGGAAGCCTTGATAGTTGTCATGGCACTTTCTTCAGGGAGAGAACCACCAGATCATGTCAATAACTTGGGTTATGAAATGCTGAATGAAGAACTTGAAGTGCCCGTGGTCAGAACCTATGGCGTAATCTTCTATCTCTTTGGGGACTTGATCTACGTAAAGTATCCGGAGGAAGAGGGCATGATCACACTGGACATGTTCAGAAACCTGACAGATAAATTCTCTGAGAGGGAGAATATTATGATTGCAACTCAACTAGGAACTGAGATCCTTCAAGAGATATACCCTTCTGAGACAGTGCTCAAAACAGTCTTTTCTCTTTGGGACAAAGGTCTATTGAAAGAGGGAAATGACTTTTACACAGTGGTAAAGACATTCGAAGCAATCATAAATGGGATGTTGATCAAGAACAATGACGGAACATACTATGATCCTTCAGCATACCTCCGAGAAACTATCATGGGGCTGCCAGTACGACTGAGGGATTATGCACAGACGTTAGTGAGTTACTTAGACTCTCTCCCCTTTAATCCACACCACCTATCTCAGATTGGGGGGTTGTTTCGGTTGTGGGGACACCCCATAGTAGATCCTAACGCTGGGGTCAGAAAGGTAAGGCTTCTGGGAACAGCTGATAAGATGAACCTGACACATATCCCCACCCTAGCAGAGCGGAAATTCAAGGAGATCTTCTACCTGTCGTATTATGAAAAGCATAGAGTCTATCCTAATCATAACTTGAATGGAGAAATTGAAGGAAGCTATTTGCTCTCTCAGCTTGCACAAATGGCTCTGGTTAATCCCAAGCATGCAAATTACTCACTAGTAGACTGGGATTCAGTCAACACACTAGAGACCTTTCCCTTTCCTAAATCATTCAACCTGTCTCTTATTATAGCGGATAAAGCTGTAAGTCCGAACAGAGAAGAATGGTTGGAATTGAGACGTAAGGGAGGAACTCAGATGGATCCGCACATTAGAAGAGGGCCTCTGAAGGCTATGAAAGACGGAGTAATCGATTGTGAGAAACTATTGAGGAAGATAAACCGTAACCCGTCCGGTTTAGCTAAGAAACATAGGATCATTGGACTGTACCCAAAGGAGAGAGAAGAAAATATGGTTCCCAGGATGTTTGCATTAATGTCCTTTGACATGAGAGCTTTCTCAGTGGTCTCTGAATCCATGATAGCAGATCACATCATTCCTCATATAGAAGGTGTCACAATGACGAAGAGCATGTTGGCCCTCCAGAAAGAGATGATAATTTCAACTAAAAGCCAGGCCTCCTCAACCCAGTCAGACAGTATAACCTTCTGTCTTAATATTGACTTTGAAAAGTGGAACCTTAACTTCCGAAGATGGATGACGGAGGGGGTATTCAGAGAAATGGGGCGATTGTTCGGACTTCCAGAGATATTCAATCGGACTTATGACATCTTCAAGAAATCCATCATTTATCTAGCAGATGGAAGCTTCGACCTCTTGCTGACAGATGAGTTGGAAATAGAACCAGGGACCAATCCAGATTGCGCATACACCGGTCATGTTGGGGGGTTTGAGGGATTGAGGCAGAAAGGATGGACAGTCTTCACAGCGGTCTTAATCTCTTCCATCTGCGATGAAATGGGGATCAAGACTCATCTAATGGGGCAAGGTGATAACCAGGTTTTGATGCTGACCATCTACTCAAGAGCTGCTAGAGAGACGGGGGATTTGAAATCAGCACCAGCAGTACTAGAGATCACAAATACATTGGAGACATTCAAGAGCAGACTAGTCTCACTCTTTGCTAATCTTGGATTGCCTATAAAACCACTAGAGACCTGGGTCTCAGAGGAATTGTTTGCCTATGGCAAGACTCCAATATACCGAGCAGTCCCCCTTGCAATGAGTCTCAAGCGAATCTCTAGAGTTTTTGCGTTTTCCAACGAGGATCTAATGACCTTGTCTAATGCGTTAGGTGCTATCTCTGCGAATGCACAAGCTGCATCCATGTGCGATGTCCATCCGATGGTGTCATATGCCATAGCAAAGTGGCAACACCTATGCTGTGCTATAATCTTCTCCAACTATCATCCGCTATGCGGGTGTGCTCCACATGTCTCAGGAGAGGAATGGGCAATAAAGCTACGACTTCCGTCAGGAAAGAAGATTCAAGAGACATCTGATGAGGAGATAGACGAAAGAGATCTGATGAAACTAATAGTGACCATACCTCGAAGCTTAGGAGGTTATAATACCCTGACTCTGTATGAGATGATCATGAGAGGATTTTCTGACCCAGTCTCTAGGGACATGTGCTGGCTATTCGCAATCGCCAGTGAATCAACAGGAAAACTTCGAGGTTATCTGATAAACTGGATTAAACCAATCGTGTCTCCGGAAGTAAATGCTCAGCATCTCATTCAGGATCCCACGGCACTCAATTTGCTAGTACCCCCTAATGCTACATCAGTTATTAAGAGGATGATAGACAAATCACTCGAAGCACTCCCAAAGAGATCTCAGTTCGCAGTATGGTTCTCTGAGATCCTAGAGATCTCAGGGGACAAGGAAATCTCAAAGTTAGCAGAGGCCTTGACCAGGACAGATTCCCTGAACCCCCGTTTCCTTCACGACATTCTGGGGGCCACTCTTTATGGATACTGCACGGCTATAACTAGTAAAGTTGACAAGACAGTGACCTTATCTCGGATGGCACTAGCGTCTAAGGATGTTGTTGGAGCTCTAATCAAGGGGGAGATGAGACTTTACTCCTATTTCGGATGGAGAACCTTGCAATCGAGAGGCTTACCTCTGACCACCAGATGTCCCAATAAGTGGGTGCGGATAATTAGGGATATCTCATGGCAAAAACAAATTAAGGCTGTTAGCGTTCCGTATCCTACTCATTTCTTATCAGAGGATATTTCAGAGACTGATAGGCCCGATTCCTGGATTGAGTGCTATATTGATGATGCCCCGACATCTGACAGAAGTTGCATGATCTATTCGACAGGAAAAGCTCTGCCGTACTTGGGTAGTGTCACTAGAGAAAAACTGACAACTCGAGGTGCAAAGGCGGCCTATGGAACTGAACCTCTCGTCTTGAGGCCAATTAATCTAGTTAGAACTATAGGTTGGTTCATTGAAGAGGATAGCAATTTTGCTGAATTGATCAAGATGCTACTGGGAGCCGTCACAGATCTTCCCATAGAGGAGGTACTCTATATCCCAGAAATGGTGAGTGGCTCTATGGCACATAGATACCTAGATATGTCAACACAGCATGGATCTTTGTGGATGCCTCTTTATGGTCCTGCCACTTTTCTCCATATGAGCACTAATACTTTTGTGCAGTACCTGAAGGGGACAGAAAATGTCACTTTGCACTTTCAGTGTGTCATGGGCTTAATTCAGTATGCTATAGTCAACAAAGCTCTAGGGGAATGTCCCACAAAGAGAATGACGAGATTCTTCCGATCATGCCCTGACTGTATAGTTCCCATTGATGATACGTTGGAGGACTTGCCTGAGGTCCCTTCGCTAGATCTCATCCCTGAGAGGACCACAAACCCTTATCTCTACCTCAAGAAGGAGAAGATTGAACTGAATGTTAGGCATCGACTTGCAGTGATAAATGAGATCCGGGTAATAGCTAGGGATGAGATAGAAGAGACTCCCCTGTTGGCTAACAATACTCTGGAGGACGTGATGAGTTTGAGGGCAGCTCAGAGAATATTCTACACAGCCAAAGGAAAGGAGGCACAGTGGGACTTGCAGACTGCTGATCGAGAGGGGTACTTAAAACTGGATTTCGTGAGCGTAATCAGGAAGATTATAGGACACCTCTTTGTAATGGAATCAGAGACGTTAAAAGCCGGGTCAGACTACCCTACTTTCCAGTTACAGCAGAGAAGGATCATGCGGCGCGTTAGGCAGTCAGATACATCTAACTTTGTCCACTTAGGAGGGTTCTTCTGCTGGCAAGAGAGCATTGCGAGGATACAGAAACTGAAGTGGTCAGTGATGCCTGCCACATTCCCTATCACTGCAGAATCAGTGTCACTGGCAGCTAAGATGAGTCTAATAGGGGCAATGGCTAGCGGGCTAACACCTAAGAGATGTGATGGTGTTCTGGCCGAAAACTTAATCCCGGATGTCACAAGGCAAGCAAAGAACATCATATGCCTGGACAAAGTGTTCCAAAGCAGATGTGATTACTGCTATACTGCAGCAATGACAAATAGGTGGTCAGACTCGATAAACTCCGACAGCATATTCAATATGAGATGTGAGAGAGGCCATACCATTCTGTCCCCGAGAATGTTGCATCAATTGAGGCGAACTATCTTGCCTGAAGGAGCCCTATACACTTTGGCTGTCAGGGTGGTTCACCAAAGCCCTCCTGAAGCATCACAACCTCTTCAGATCATCCCATGCAGGCGAGAGAGATATCAAATCTTGAGTGAAGCTGACATGCCTAGACAAACTGGCTACTCGGGGGTCTATAGAAGGCCTACCAATCAAATCTGCATGTATACAGAGGTTGAATTGGCAAAGAAGTACAGGTTGCCAACTAATTCATTATACAGGATCCTTGACTTACACGACTGCTTCTTCAGTCAGAAACTCATGGACACAAGGGGCAACATTTTAGTGGTCGGAGACGGGTATGGATACAGCTCCTTGCTAACCAAGTGCTTGAACCCAGACAGGAATGTAGTGAGCTGGACCTATATTGAACCATCTGAGGCACTTCCACATAGCCTGAGAATCTCTAAGCCTCCAATGCACTACAAAGCTGATGTGCAGATAGATTCTAGCCCTTCAATTGACAGAATCTCCGACATACATAACTCCTCATACCCGGATGAGTTCGCTAAAGTGGTAACCAAGAATGGGATTACAGCACTGATCAGTGACATTGAAACTGTGTATGTCTCTGGAGAGAAGAGTGTGGCGTCTTTGATCAACTTGGCTTGGAATAACCAGATCCAATTAGGAGCACTCAAGTTGGAATTGATGACGGATCCCTTGGAGAAAGTCGTAGAATATGCCCACAATGCTTACCAAAGATGGGAATTATTCACTCTACCAGGCGCCAATCTCGGAGGAGGGGTACTATACATCGGTTTCTACGGTAGAAGAGAGAAGTTGACTGGGTACATCATTCCCCATTCAGGAGTTGAGACCTTGATGGATAGGTTGGCAACTGAGGTAGATGAGGATAGAGGAAGATTGAGAGCAGAGGACCGTGATCGGTGGGAGCAGTTGGATACAATGGAAAGCAAAATACATCTGCAGCAATACCATAAGATGAGGCTCGATCTATGGTTCGGATCTGCTTATCTGAGTGGTTATCTCTCAGAGGATATGACTGAGTTCTTTTACTCAGTAAAAACATCTTACAGACCACCTGCAATCTCATGGGAGAAAGGGAACCAGGCCAGATACCTCTATGGCAGCAGGGAAGAGATCTTATTCATGAGCATGATCACAGTGGCACTATCATCCTATCTAGAGGACCAGGATGTCTTAAGAGAGTTCCTTTCTTCCTCAGGGTGGAAACTCCAGTGGAAGAAGGATAAGAAGAATCCGAGGCATTGGTCCCCGTATTTGGAGCGGAGTGACTCTCAACTAGTCTGGAAAAAGAACAAAGCCATGATTTTCAAACTGGTATCAGTTCTGAGAGGATCAAAGCCGAGAGAAGAATATAAGATGGGAAAAGCATCTGACTCCATAGTTTTCAGATACATTCCGAGGAAGGTAAGGGAAGGAGAAGTTCCCCTGTGCTTCCCGATCTCTAAGGTGGCCTCAATGTCGGTATAAGAAAAACGAGACCTTCATCATGGATGATTTAATGGTCTATAGCCTTTGGGCTCTGCTGCAATGGGGTTTGCCTGCGGATACAGCCAGATACATAATCTCACTGAGGATGGAAGAAGACTACTGGGAAGAGATGAATGAACATGAGCAGTGGGACGGGTACGAGTCTTGGGATGATACTGATTGGCAGTACCCGAGTCCTTGAATGAGTCAGTATGTGACAATTTAGAAAAAACGATTACCTTACTGTTGACTTTAAAGAACACTGTTATGTTGTGTTCGCAACACACAACTTCCTT

>Seq12 Rice stripe mosaic virus TM isolate, complete genome

AAGGAAGTTGCGTTGCGAACGCAACATTAAAATGTTGACATAACCCACCTTATCATAACAAAAGAAAAACGAGACCGCCAAGTCTTAAAATGGCAACCGACAAGTCTTTTGAGGAGAAGCTGAGCCTGGTTCCTGAGAACACTAAGCTATATTCAATATCTCCTGAGGCATATTCAGATGATAAGTTTGATAAGGCCAATTGCTACAAGCTAGAGAAGAGGTCTGAATATGAGCTAACTAGGCTCTACAAAGGACTAGTCCGGGACTTAGGGAATTCTTCCCCCTCCACTTATGCAGTCGAGAGATTGCTAGTCTTAGCAAGCCATCTGTATGAGACCAAGAAAGGGTCTTCTAACTTCTTCTTGACCGATTACCTGCCCAAGACAACATCAACAGCAAATCTTGATGCAGGCTTTTTGGCAAAGCTGAAAGAGACCCCTAAGGCCTCTGACCCAGACGTTTCCGATGTGACAGAAGTCAAGACAGCTAAGGCTACTCTAGACTCTGCTACAACTGATGCGGATACTAAAAAGGCCGCTTATGAGGCTATCGGAGATGAGGATTCAAAGAAGGCTGAGAAGGCTACAGCAAACACTGCCTGGATTGCTGCCCAAGAGGCCCAGAAGAAGGCTCAGAGTGCCTATGATAAGGCAGTTTCGAATGCGAAGAAGGCCTCAAGGAAAACTACCTCTGGAAAAAGCTTGTTCGGGGATGCAGGGGAGACAGTCACAGATGAGTCTAAGGTAGTGGAAGAGGTTGGAGAGGGGAAGAAGAAGTTTGGACCCTTCTTAGCTGCTTACTTGATGAGGCTACTGACCAAAATAGCATCCAATGTCACAGAGTCATGGGAGCATATGAAAGGAATGTATAAGAACTTTTATGGTTATGATGCTCCTTCAGACCTGAACTGTCCCGAGGCAGGATTCCTTGAGCAACTGAAGTCTGAGCTTAACAAGGATAGAAGGGCGGCTACCTCATGGGTCAAAATAGTAGCAGAGGCAGATAATAAGTTGGATCAATCTACAGCCGAGGCTGGGATTCTCCGCTATGTGGCTGTCCTCCCTCTTGCATATTCTGGGATGCATGCGATGAAGTTGTTCATGGACGTCAAGATGCTGACCAAACTCACCAGCAACTACTTGATTGGTGCCATGAGGAGCCCTCTGACCAAGGATGCATTAGATGCAATCATGGATATCTTGATCTCGTTTGAGTCCACTACAAAGACTAAGAAGTCGGAGAAGTTCCGGTTTGCTAGGATTGTGAGTACTCAATTCTTTCAATCCCTCCAGACCAAGAATTGCAAAGAGCTGGTCTATCTGATGGTTCAGATAATTGCTGAGTACAGAAAAGCAGAGGGGGTCAGGGATCCCATGAACATTGCTGGATTAGATGACATCTCTAGCAGGAACAAGAAGAAGTTGAATAAAGCAGTGCGGATAATACTGGCAGAAGCTCCAAAGGCATCCGCAGGTGAGTATTCGTCAGCCATGAAGAAGGCTTTCCTGGATGACGAAGAGGATGATACAGCCAAGACCAGGTCCATCTTCCAGACCAAGGCTTAAGGAGCATCATGTTTGGATAGAATGTGACTCCTCTAAATAACTATGCAGCCTACTGGCGAGTCTTTGTGTGTTATATAAGAAAAACGAGACCGTCAGGTCAATAATGAGTGTTCCAGAGGATACTCCCTTCAGATCGTACTCCAGCATCTTTGACGACTCAGACTTTGTCCAACCACAGCCCATGTCCTTCAAAGCGACCAAGGAGTCAGAGAGCCTTCCTGAGACAGAGAAAGAAGATATGTCGACTGAATACCTCTCTGAACCACTGAGGACCAAGTCAGGTAAGAAGAACAGACGGAGGAAGGGAAAGGACCTAAAATCTCTCTTCACTCAAGAAGCTGGACTCCCGGCTCCAGAAGCAGACAGTGTCCTCCCCGAATCGTCTCCGTATGAGAATGATAACGCACAGTTGGAGCTACCCAAACCCATTCTAAAGACATCAGACGCTCCGGTCTTCCTAAGAGAGAAGGATCTTAGTAAAGAATTTGCTGCAGCCTGCAAGACTAATGGGATTCTCCCAAGGGATGAATGGAAGTCATCTGTAGCAGCCAAGTATCATGCAGAGGAAGGGAAAATGACCAAACGAGACATCTCGTTAATCGTATTTGGAATGGAGCTTTACAAGAGATACAATGTGGAATCTGAGGTATCAACTTTGTTCACTTCATTGGTGACTGAGTTGCAGGGGATAAAGGTTGCTGCTAAGGAGTTGAATGATACCCGGGAGGTCCTCACTAAGATTCCAGGAGAAATTGTGTCTGCTGTCAAGGCAGGGGTAAAGGAGGGGACCGAGATGGGGATGGATTATATAGAGACTAGAACTAAAGTGGCCCCCAAGAGTGCTCCTAAAGTGGACATCTCAAAGCCGATGAGTAGTAAGATGATGGAGCAGCAGGATGAGAGTTCTGATGAGTCCTCAGATAATGAGAGTGAGGAGAGTGAGGAAGAATCCTTTGAGACAAAGGCTGCAATCTTCTTAGCTTTGATAAAGGTTCCAGAGGAAGAGAGAGACAACGCAATAGTCCTTATGGCCTTGAGGGCAGTGATATCTGACAGTGAGCTGAATCAGGCAATTAGGAATGACAGAATCTCCTCCTCAGTAGCAGATATGTACCATCAAAAGATATCTGACAAGGCTAGGGAATTGATGGGAAAGGGAAAGACCAACAAGAGGGCCAAGCAGCCTAAATCCTCTAAGTATGCATCGGATTACTATGATGATGCACTGTGAGTAATCAGTTACTACCTGTGTGGCAATGTCGGAACTGTACTTACCTATATATTGATAATCGTCTGTGATCTTGTGCCTTTAATTGCTGCAGTATTATAGCTTAAAATAATCTAGTTACCAATGCTCTATTTTCGTACTTAAGTGCCATGTTGCCTGCCTTTACCAAAGTAATCTAGTGTGCTCTATTTAAGAAAAACGAGACCTTCATCATGAAGATCATCTGCAGTACTGGGATCTTCAATGAAGAGAAAGGCTTCCCCCTCCCCAACCTCCTCAACAGTCCCTTGATGAAGCAGGAGATCATGACGGTCAAGTACTTGAGGTTTCAGTATATCCCTATTATGACCAGCAACCCCTCGAGTTCATTGACTATTGACATTAAAGACACTCGACTGGTCAATTGGGATAATAGGTCGATTTTCCAGGTCAAAATATTCGGGGATGTTCAAAGCTCATTTATTGTATCGGGTCTACAGCCTTATTCAGCTAGAGACCGTTGCCCTTATCTCCTCTCCCTTTCAGTAAACGCAGGGAAGGTTGTTCCAGGGACCAAATACGGCATCTTAAAATCTTATGCTGTGTACACATCTAAAGACTCAGGGATAATTTCGTCACAGATCTCTGTAAAACTTGAAAGGTCCCCACGTGACTACTTCTTGAAGAGGTCTAAGGAGCACGACAAAAAAGATCTTGACAGTGATGTCTCATTCAAGATGTGTCGCCATGTCAAGTTTGCTACTTGAAGCTCGGAGAGGATGGGCAGCTGAATGTTGTCGGGGTGATGTTTTCCTTATGAATATCTTCAATAATTTCCCTGGAGGGAGAAACAGTTGATCTCTGGGAGGACGCAAGGGCTCACTACTAGCATGTGCTTGCCAGGTTGAGGTTTTATGCCTATCATGGTTTAGCCTAAATAAGAAAAACGAGACCATCATGGCCGTTCCGTGGACTGAGGTTAAGGACTCCAAGTACTTGGCAACTAAGATGTCTGTTACCCTGATCATGGAGATGAATGAAGATACACCTCTAAAGTATCCTTCTTACAACGCATTCGAGAGCATCTTTAAAAGGCTAGCAGAACCAGAAAGCGCAGCACCCCAAGTGGCCGCATGGTTTACTTGGTTCCTAAGAGAGGCAAAGGACATCTACTATCTGGAAGTGAGTAACAAGGAAACTGCCCAATACGGACCAACTAAAGTCTATAAACTTCAGTGTCCAGCCTACCTCTTATCCCGAGTGACAGGAGGATCACAGCTTGATTATACCTCTCTTATAGGTAGCAAGGTGATGACTGAGAAAGATCGAGGGATCCCAGTCAGAACACTCTATATTACTGGAGGGGGTACATCATTCAGAGTCATCAACGAGGAGACAGCCAACCAGTTCATCATAAATGATAATGCTGTCCGCCTACCAGGAGAGTGCAAGGTGGATGGAGGAAGTATAATCTGGAGTTAGAATTTCAAGAAAACTAAACAATAATGAACAAGTGGAACAATCTCGTGTAGTGTATTAGGTGTCCTCTTTAACTAAAGTTAAATAAGAAAAACGAGACCCGCAAGGATCTTAAGATAACAAGATGATGAGGATTTCGGTCTTTCTCTTGATGCTCTGCTGGCTCCCTGTCAGCCTGACCTTCTTCGACAAATCACACATTCCTATAACCACATGTGATAAGAATCTAATGAGTCCTATCCCTTGGAGAACTTACTGCATAGAGGAGTGTGGAATCCGGAATGTAATAGGTGATAAGCTGGATCTGTTTATCTACAATAGGTCTGACAGTGGGAAAGTACAGCTGGCTGACTGCAGGAAGTACAAGATCAGACAGACCTTCACCAAGACTTGGACATTCTCGACATTCAAAGGGGCGATAGAGACAGAGGAGCTAATGCCTAATTATGCAGAGTGTGAATCCACCTGGAGAGATCTGTGCAACTCTGGACCGTGTAGTACCACAACTCCGGTGATCCCTGAGGAATACCATTGGGCGTCTGACACCACGAAGGAGGTCATCTATGTATCTATAGATGCATACCAGAAAACTGTTGCATTCCAGGATCCTAGTGGTGATATCCAGCTCCTGGTCCATGGGGTGATCATAGATGGGAGCCAGTCTGGTTATGTTCAACCCAGCAAAGATCTCATCACTATGTGGGATAAGGTTGAGTTACAAGATGAATGCCCTTGGTCAACGGGAAATTCTCTTTCGTGTTCTACGTCTGATGAGGGAATTTCATACTACTGTGCTGGGAAAGGTCTAGTACTGACCAACATCAGTACGGTGACTGATACCAGATGTGACAACAACCCGCACCTAATGACATCAGGGCACCATGTGATTTTTAGAGTGAAGAAGGCATCAGACCCGAATGCGACTCTCAGCAGGACAGCTCAAATAGTGCTGGACAGGGGGTCAGAAGAGGCCGAGATTGTAGATAGTGTTAATAAGGCGTTGCTGGATAGAGATTCCATCAGGTGTGCAAGCTCATGTCTCGCCTTTGATTACACCATCTCCAAGCCTCAGATGTTTGGCAACCAATTGGCGCTACCTTATAAGGGGTCTTTTCTCCCTTGCAACATACTGCCTAATTGTCGGGTTGTCTTCCCAGTCAAGTATTGCAGCTCTCCTCCGATGATTCTGGTAGAATGTACCGGCACTATGACATGGTGGAATATCACTGGAGATTACACGATCAGACCCACCTATTGCCACATGAACCAGTCGGCGACCAAGATTAAGACATCTATATCCTTTATGACAACAAATGGGAGAGTCTTAGTGAATGAGTCTGGCGCTTATCCTGTCTCCCGTGAAATAGGAAATACGTTCCAGGTCGGACATGTCATAGAGCCTAGCTCCATGATAGAGGTGACTGATCCACTTAATGTTAGGATAGATGACACCTTAGTCACACCAGAGTCCCATACTATATCTAATATCACTTCAGTCGGGGACTCACTTTTAGATACAATGGTTGAGACCGTGAAAGGTATCGGTCGCTTCATATCCCATGAGGTCAGGATAGTGGTTTTTGGTGTTCTGACTCTTTTTATATTATATCTGTCGTTTAAATATTTGTTCGCTAAGAAGAAGAGCAGAGTGCCGCACCCGAAAGTTGTCTATACAAAACCCACATCTGAAGGACCAGTGATCTATGACACCGAATATACTATAGAAAGTGACTAATAAAAAACAGAGACCAACATGGAGTTCAATTGGCCTTGGGGACAGAACAGTGAAACGGAAATCACCAAGAATCTCCGCTTTGAGGACATTAAGGTGATGGCCATAATAATACTAGTCTGGGTGAAGTGTCTTCTCATCTACCATTTCAAGAGGAAAATAAGGCGACTAAGATCTCTATTGATAAAAGGATCCTCACAATGGGTACTGCATGATGCCTAACTCATTAGGAAGATCTGAGATGATTTTTTATCCTGCCTTATCCTTCTAATTAAGAAAAACGAGACCGCCATCATGGACCTTGATGACGGTGGTCTATGGAGACGTGCTAGGGGTCTGGGGGATTATCACCTGAGGTCTGCTCTGGTGACCCCCTCCTTAGAGCGTTTCCGCAGTCGAAAGGGAAGGCACCGTGAACAACTGTGCTTTGATAGGATGAAATCACTAGGCTGGATGTTGAGGTGGGTAGATCAAGGGAAACTGCTTGGATATTTAATGGTAGAAGCCAACAAATCTTTACCAAAATCCATTGCAAACCAGGAACTCCTAGTTGAGACACTGAAGTTAGAATACGGATGTCTTAGACAGATAATCATGACAGATGGAGACCTCCATGATCAGGTGATCTCTTATCTAGACAGGAAGTCTATCTCTACCCATTACACCCATGGTAGGGAGGTCTTTCAGGAAGCCTTGATAGTTGTCATGGCACTTTCTTCAGGGAGAGAACCACCAGATCATGTCAATAACTTGGGTTATGAAATGCTGAATGAAGAACTTGAAGTGCCCGTGGTCAGAACCTATGGCGTAATCTTCTATCTCTTTGGGGACTTGATCTACGTAAAGTATCCGGAGGAAGAGGGCATGATCACACTGGACATGTTCAGAAACCTGACAGATAAATTCTCTGAGAGGGAGAATATTATGATTGCAACTCAACTAGGAACTGAGATCCTTCAAGAGATATACCCTTCTGAGACAGTGCTCAAAACAGTCTTTTCTCTTTGGGACAAAGGTCTATTGAAAGAGGGAAATGACTTTTACACAGTGGTAAAGACATTCGAAGCAATCATAAATGGGATGTTGATCAAGAACAATGACGGAACATACTATGATCCTTCAGCATACCTCCGAGAAACTATCATGGGGCTGCCAGTACGACTGAGGGATTATGCACAGACGTTAGTGAGTTACTTAGACTCTCTCCCCTTTAATCCACACCACCTATCTCAGATTGGGGGGTTGTTTCGGTTGTGGGGACACCCCATAGTAGATCCTAACGCTGGGGTCAGAAAGGTAAGGCTTCTGGGAACAGCTGATAAGATGAACCTGACACATATCCCCACCCTAGCAGAGCGGAAATTCAAGGAGATCTTCTACCTGTCGTATTATGAAAAGCATAGAGTCTATCCTAATCATAACTTGAATGGAGAAATTGAAGGAAGCTATTTGCTCTCTCAGCTTGCACAAATGGCTCTGGTTAATCCCAAGCATGCAAATTACTCACTAGTAGACTGGGATTCAGTCAACACACTAGAGACCTTTCCCTTTCCTAAATCATTCAACCTGTCTCTTATTATAGCGGATAAAGCTGTAAGTCCGAACAGAGAAGAATGGTTGGAATTGAGACGTAAGGGAGGAACTCAGATGGATCCGCACATTAGAAGAGGGCCTCTGAAGGCTATGAAAGACGGAGTAATCGATTGTGAGAAACTATTGAGGAAGATAAACCGTAACCCGTCCGGTTTAGCTAAGAAACATAGGATCATTGGACTGTACCCAAAGGAGAGAGAAGAAAATATGGTTCCCAGGATGTTTGCATTAATGTCCTTTGACATGAGAGCTTTCTCAGTGGTCTCTGAATCCATGATAGCAGATCACATCATTCCTCATATAGAAGGTGTCACAATGACGAAGAGCATGTTGGCCCTCCAGAAAGAGATGATAATTTCAACTAAAAGCCAGGCCTCCTCAACCCAGTCAGACAGTATAACCTTCTGTCTTAATATTGACTTTGAAAAGTGGAACCTTAACTTCCGAAGATGGATGACGGAGGGGGTATTCAGAGAAATGGGGCGATTGTTCGGACTTCCAGAGATATTCAATCGGACTTATGACATCTTCAAGAAATCCATCATTTATCTAGCAGATGGAAGCTTCGACCTCTTGCTGACAGATGAGTTGGAAATAGAACCAGGGACCAATCCAGATTGCGCATACACCGGTCATGTTGGGGGGTTTGAGGGATTGAGGCAGAAAGGATGGACAGTCTTCACAGCGGTCTTAATCTCTTCCATCTGCGATGAAATGGGGATCAAGACTCATCTAATGGGGCAAGGTGATAACCAGGTTTTGATGCTGACCATCTACTCAAGAGCTGCTAGAGAGACGGGGGATTTGAAATCAGCACCAGCAGTACTAGAGATCACAAATACATTGGAGACATTCAAGAGCAGACTAGTCTCACTCTTTGCTAATCTTGGATTGCCTATAAAACCACTAGAGACCTGGGTCTCAGAGGAATTGTTTGCCTATGGCAAGACTCCAATATACCGAGCAGTCCCCCTTGCAATGAGTCTCAAGCGAATCTCTAGAGTTTTTGCGTTTTCCAACGAGGATCTAATGACCTTGTCTAATGCGTTAGGTGCTATCTCTGCGAATGCACAAGCTGCATCCATGTGCGATGTCCATCCGATGGTGTCATATGCCATAGCAAAGTGGCAACACCTATGCTGTGCTATAATCTTCTCCAACTATCATCCGCTATGCGGGTGTGCTCCACATGTCTCAGGAGAGGAATGGGCAATAAAGCTACGACTTCCGTCAGGAAAGAAGATTCAAGAGACATCTGATGAGGAGATAGACGAAAGAGATCTGATGAAACTAATAGTGACCATACCTCGAAGCTTAGGAGGTTATAATACCCTGACTCTGTATGAGATGATCATGAGAGGATTTTCTGACCCAGTCTCTAGGGACATGTGCTGGCTATTCGCAATCGCCAGTGAATCAACAGGAAAACTTCGAGGTTATCTGATAAACTGGATTAAACCAATCGTGTCTCCGGAAGTAAATGCTCAGCATCTCATTCAGGATCCCACGGCACTCAATTTGCTAGTACCCCCTAATGCTACATCAGTTATTAAGAGGATGATAGACAAATCACTCGAAGCACTCCCAAAGAGATCTCAGTTCGCAGTATGGTTCTCTGAGATCCTAGAGATCTCAGGGGACAAGGAAATCTCAAAGTTAGCAGAGGCCTTGACCAGGACAGATTCCCTGAACCCCCGTTTCCTTCACGACATTCTGGGGGCCACTCTTTATGGATACTGCACGGCTATAACTAGTAAAGTTGACAAGACAGTGACCTTATCTCGGATGGCACTAGCGTCTAAGGATGTTGTTGGAGCTCTAATCAAGGGGGAGATGAGACTTTACTCCTATTTCGGATGGAGAACCTTGCAATCGAGAGGCTTACCTCTGACCACCAGATGTCCCAATAAGTGGGTGCGGATAATTAGGGATATCTCATGGCAAAAACAAATTAAGGCTGTTAGCGTTCCGTATCCTACTCATTTCTTATCAGAGGATATTTCAGAGACTGATAGGCCCGATTCCTGGATTGAGTGCTATATTGATGATGCCCCGACATCTGACAGAAGTTGCATGATCTATTCGACAGGAAAAGCTCTGCCGTACTTGGGTAGTGTCACTAGAGAAAAACTGACAACTCGAGGTGCAAAGGCGGCCTATGGAACTGAACCTCTCGTCTTGAGGCCAATTAATCTAGTTAGAACTATAGGTTGGTTCATTGAAGAGGATAGCAATTTTGCTGAATTGATCAAGATGCTACTGGGAGCCGTCACAGATCTTCCCATAGAGGAGGTACTCTATATCCCAGAAATGGTGAGTGGCTCTATGGCACATAGATACCTAGATATGTCAACACAGCATGGATCTTTGTGGATGCCTCTTTATGGTCCTGCCACTTTTCTCCATATGAGCACTAATACTTTTGTGCAGTACCTGAAGGGGACAGAAAATGTCACTTTGCACTTTCAGTGTGTCATGGGCTTAATTCAGTATGCTATAGTCAACAAAGCTCTAGGGGAATGTCCCACAAAGAGAATGACGAGATTCTTCCGATCATGCCCTGACTGTATAGTTCCCATTGATGATACGTTGGAGGACTTGCCTGAGGTCCCTTCGCTAGATCTCATCCCTGAGAGGACCACAAACCCTTATCTCTACCTCAAGAAGGAGAAGATTGAACTGAATGTTAGGCATCGACTTGCAGTGATAAATGAGATCCGGGTAATAGCTAGGGATGAGATAGAAGAGACTCCCCTGTTGGCTAACAATACTCTGGAGGACGTGATGAGTTTGAGGGCAGCTCAGAGAATATTCTACACAGCCAAAGGAAAGGAGGCACAGTGGGACTTGCAGACTGCTGATCGAGAGGGGTACTTAAAACTGGATTTCGTGAGCGTAATCAGGAAGATTATAGGACACCTCTTTGTAATGGAATCAGAGACGTTAAAAGCCGGGTCAGACTACCCTACTTTCCAGTTACAGCAGAGAAGGATCATGCGGCGCGTTAGGCAGTCAGATACATCTAACTTTGTCCACTTAGGAGGGTTCTTCTGCTGGCAAGAGAGCATTGCGAGGATACAGAAACTGAAGTGGTCAGTGATGCCTGCCACATTCCCTATCACTGCAGAATCAGTGTCACTGGCAGCTAAGATGAGTCTAATAGGGGCAATGGCTAGCGGGCTAACACCTAAGAGATGTGATGGTGTTCTGGCCGAAAACTTAATCCCGGATGTCACAAGGCAAGCAAAGAACATCATATGCCTGGACAAAGTGTTCCAAAGCAGATGTGATTACTGCTATACTGCAGCAATGACAAATAGGTGGTCAGACTCGATAAACTCCGACAGCATATTCAATATGAGATGTGAGAGAGGCCATACCATTCTGTCCCCGAGAATGTTGCATCAATTGAGGCGAACTATCTTGCCTGAAGGAGCCCTATACACTTTGGCTGTCAGGGTGGTTCACCAAAGCCCTCCTGAAGCATCACAACCTCTTCAGATCATCCCATGCAGGCGAGAGAGATATCAAATCTTGAGTGAAGCTGACATGCCTAGACAAACTGGCTACTCGGGGGTCTATAGAAGGCCTACCAATCAAATCTGCATGTATACAGAGGTTGAATTGGCAAAGAAGTACAGGTTGCCAACTAATTCATTATACAGGATCCTTGACTTACACGACTGCTTCTTCAGTCAGAAACTCATGGACACAAGGGGCAACATTTTAGTGGTCGGAGACGGGTATGGATACAGCTCCTTGCTAACCAAGTGCTTGAACCCAGACAGGAATGTAGTGAGCTGGACCTATATTGAACCATCTGAGGCACTTCCACATAGCCTGAGAATCTCTAAGCCTCCAATGCACTACAAAGCTGATGTGCAGATAGATTCTAGCCCTTCAATTGACAGAATCTCCGACATACATAACTCCTCATACCCGGATGAGTTCGCTAAAGTGGTAACCAAGAATGGGATTACAGCACTGATCAGTGACATTGAAACTGTGTATGTCTCTGGAGAGAAGAGTGTGGCGTCTTTGATCAACTTGGCTTGGAATAACCAGATCCAATTAGGAGCACTCAAGTTGGAATTGATGACGGATCCCTTGGAGAAAGTCGTAGAATATGCCCACAATGCTTACCAAAGATGGGAATTATTCACTCTACCAGGCGCCAATCTCGGAGGAGGGGTACTATACATCGGTTTCTACGGTAGAAGAGAGAAGTTGACTGGGTACATCATTCCCCATTCAGGAGTTGAGACCTTGATGGATAGGTTGGCAACTGAGGTAGATGAGGATAGAGGAAGATTGAGAGCAGAGGACCGTGATCGGTGGGAGCAGTTGGATACAATGGAAAGCAAAATACATCTGCAGCAATACCATAAGATGAGGCTCGATCTATGGTTCGGATCTGCTTATCTGAGTGGTTATCTCTCAGAGGATATGACTGAGTTCTTTTACTCAGTAAAAACATCTTACAGACCACCTGCAATCTCATGGGAGAAAGGGAACCAGGCCAGATACCTCTATGGCAGCAGGGAAGAGATCTTATTCATGAGCATGATCACAGTGGCACTATCATCCTATCTAGAGGACCAGGATGTCTTAAGAGAGTTCCTTTCTTCCTCAGGGTGGAAACTCCAGTGGAAGAAGGATAAGAAGAATCCGAGGCATTGGTCCCCGTATTTGGAGCGGAGTGACTCTCAACTAGTCTGGAAAAAGAACAAAGCCATGATTTTCAAACTGGTATCAGTTCTGAGAGGATCAAAGCCGAGAGAAGAATATAAGATGGGAAAAGCATCTGACTCCATAGTTTTCAGATACATTCCGAGGAAGGTAAGGGAAGGAGAAGTTCCCCTGTGCTTCCCGATCTCTAAGGTGGCCTCAATGTCGGTATAAGAAAAACGAGACCTTCATCATGGATGATTTAATGGTCTATAGCCTTTGGGCTCTGCTGCAATGGGGTTTGCCTGCGGATACAGCCAGATACATAATCTCACTGAGGATGGAAGAAGACTACTGGGAAGAGATGAATGAACATGAGCAGTGGGACGGGTACGAGTCTTGGGATGATACTGATTGGCAGTACCCGAGTCCTTGAATGAGTCAGTATGTGACAATTTAGAAAAAACGATTACCTTACTGTTGACTTTAAAGAACACTGTTATGTTGTGTTCGCAACACACAACTTCCTT
